# Reaction-diffusion modeling provides insights into biophysical carbon-concentrating mechanisms in land plants

**DOI:** 10.1093/plphys/kiae324

**Published:** 2024-06-10

**Authors:** Joshua A M Kaste, Berkley J Walker, Yair Shachar-Hill

**Affiliations:** Department of Biochemistry and Molecular Biology, Michigan State University, 603 Wilson Rd, East Lansing, MI 48823, USA; Department of Plant Biology, Michigan State University, 612 Wilson Rd, East Lansing, MI 48824, USA; Department of Plant Biology, Michigan State University, 612 Wilson Rd, East Lansing, MI 48824, USA; Department of Energy Plant Research Laboratory, Michigan State University, 612 Wilson Rd, East Lansing, MI 48824, USA; Department of Plant Biology, Michigan State University, 612 Wilson Rd, East Lansing, MI 48824, USA

## Abstract

Carbon-concentrating mechanisms (CCMs) have evolved numerous times in photosynthetic organisms. They elevate the concentration of CO_2_ around the carbon-fixing enzyme rubisco, thereby increasing CO_2_ assimilatory flux and reducing photorespiration. Biophysical CCMs, like the pyrenoid-based CCM (PCCM) of *Chlamydomonas reinhardtii* or carboxysome systems of cyanobacteria, are common in aquatic photosynthetic microbes, but in land plants appear only among the hornworts. To predict the likely efficiency of biophysical CCMs in C3 plants, we used spatially resolved reaction-diffusion models to predict rubisco saturation and light use efficiency. We found that the energy efficiency of adding individual CCM components to a C3 land plant is highly dependent on the permeability of lipid membranes to CO_2_, with values in the range reported in the literature that are higher than those used in previous modeling studies resulting in low light use efficiency. Adding a complete PCCM into the leaf cells of a C3 land plant was predicted to boost net CO_2_ fixation, but at higher energetic costs than those incurred by photorespiratory losses without a CCM. Two notable exceptions were when substomatal CO_2_ levels are as low as those found in land plants that already use biochemical CCMs and when gas exchange is limited, such as with hornworts, making the use of a biophysical CCM necessary to achieve net positive CO_2_ fixation under atmospheric CO_2_ levels. This provides an explanation for the uniqueness of hornworts' CCM among land plants and the evolution of pyrenoids multiple times.

## Teaser

Reaction-diffusion modeling of photosynthetic CO_2_ assimilation reveals the potential and limits of incorporating partial and complete biophysical CCMs into land plants.

## Introduction

Ribulose-1,5-bisphosphate carboxylase/oxygenase (rubisco) catalyzes the fixation of CO_2_ as part of the Calvin–Benson Cycle (CBC) but is also capable of fixing O_2_. The fixation of O_2_ results in the formation of 2-phosphoglycolate (2PG), with the photorespiratory pathway being necessary to detoxify and recover the carbon in 2PG and recycle it back into the CBC. Although rubisco shows selectivity for CO_2_ relative to O_2_, substantial photorespiratory flux still occurs in photosynthetic systems due to the much higher partial pressure of O_2_ in the earth's atmosphere relative to CO_2_. Photorespiratory flux lowers net carbon assimilation and incurs substantial energetic costs, in the form of ATP, redox equivalents, and ultimately photons. Although the costs associated with photorespiration vary between plant species and environmental conditions, it has been estimated that photorespiration accounts for crop yield decreases of 20% and 36% for soybean (*Glycine max*) and wheat (*Triticum aestivum*), respectively, under current climatic conditions (Walker et al. 2016).

Carbon-concentrating mechanisms (CCMs) increase the concentration of CO_2_ around rubisco, competitively inhibiting the oxygenation reaction, suppressing photorespiration, and increasing carboxylation flux (Raven et al. 2017). In biochemical CCMs, such as C4 and CAM photosynthesis, inorganic carbon is fixed into an intermediate form of organic carbon, before eventually being released around rubisco (Ludwig 2013; Bräutigam et al. 2017). Biophysical or “inorganic” CCMs, on the other hand, do not rely on any additional intermediate organic carbon species but instead use transport-driven pumps, diffusional barriers, carbonic anhydrases (CAs), and pH differences between cellular compartments to increase the CO_2_ concentration near rubisco (Raven et al. 2008). Such CCMs are common in cyanobacteria and algae (Raven et al. 2008) but are conspicuously absent in C3 plants, including almost all land plants. This has motivated researchers to look into the possibility of introducing a CCM, either in its entirety or individual components, into these plants to improve carbon fixation, reduce photorespiratory CO_2_ and energy losses, and ultimately boost yields (Ermakova et al. 2020; Hennacy and Jonikas 2020).

The seemingly substantial benefits of CCMs raise the question of why they are not already more widespread in land plants. Despite their lack of a CCM, C3 plants are still the most abundant group of land plants in terms of vegetation coverage and gross photosynthetic productivity (Still et al. 2003; Raven et al. 2017). In the case of C4 photosynthesis, the large number of anatomical and biochemical features required has been invoked as a reason why, rather than being universally adopted in land plants, it has instead repeatedly evolved only in lineages exposed to the kinds of hot, arid conditions that limit water availability and exacerbate the losses associated with photorespiration (Sage et al. 2018). However, such an explanation is less satisfactory in the case of biophysical CCMs because they are present in the hornworts. It also raises the question of why biophysical CCMs are uniformly absent in all land plant lineages except for the hornworts (Villarreal and Renner 2012).

Have inefficiencies associated with biophysical CCMs precluded their successful emergence in C3 plants and can we examine the presence and absence of these biophysical CCMs in different groups of organisms using these inefficiencies? The efficiency of intermediate photosynthetic configurations, featuring some but not all the essential parts of a CCM, may also represent a barrier to the emergence of CCMs in land plant lineages. Anatomical and life history details of hornworts may explain why, among the land plants, only hornworts have evolved pyrenoid-based biophysical CCMs (PCCMs) and have done so repeatedly (Villarreal and Renner 2012). The poikilohydric life history of hornworts makes it necessary for them to have highly desiccation-tolerant cell walls which, together with bryophytes' generally thicker cell walls (Flexas et al. 2021) and hornworts' simpler tissue architecture, may explain their extremely low gas conductance (Meyer et al. 2008; Carriquí et al. 2019). We hypothesized that the distinct morphologic characteristics and habitat of hornworts may explain why they, uniquely among the land plants, evolved biophysical CCMs. It is possible that the different paths that inorganic carbon has to take from the environment into a C3 land plant cell versus an algal cell can similarly explain why the former never uses pyrenoids to concentrate carbon and the latter frequently does.

A closer examination of the costs of a CCM may also inform the viability and strategy of biotechnological projects focused on introducing them to C3 crops. Prior quantitative modeling work argues that incorporating individual CCM components—in particular, bicarbonate transporters at the chloroplast membrane—and entire CCMs into land plant systems may boost net CO_2_ fixation as well as improve the efficiency of photosynthetic carbon assimilation by reducing the energetic costs associated with photorespiration (McGrath and Long 2014; Fei et al. 2022). Similar arguments have been made in favor of engineering biochemical—e.g. C4—photosynthesis into C3 plants (Walker et al. 2016). These models represent sophisticated, integrative descriptions of photosynthetic carbon assimilation. For the purposes of the questions we are interested in, however, we needed models of both land plant and algal systems that represent photo-assimilatory processes at the whole-cell level. We also needed models that allow us to explore substantial uncertainties in certain key parameters and that include energy costs associated with CA activity in the thylakoid lumen.

Here we developed spatially resolved reaction-diffusion models of land plants and green algae with and without PCCMs in the *Virtual Cell* platform (Schaff et al. 1997; Cowan et al. 2012). These models represent C3 land plants containing PCCMs, as well as algal systems containing biophysical CCMs going beyond the scale of the chloroplast and including the whole cell in an aqueous environment. We highlight the substantial uncertainty in reported or predicted values of the permeability of lipid membranes to CO_2_ and explore how this uncertainty can give rise to qualitatively different conclusions as to the efficiency and effectiveness of adding chloroplast envelope bicarbonate pumps in particular. Finally, we find that despite the near-ubiquity of biophysical CCMs in algae, modeling suggests that lower levels of external inorganic carbon (dissolved inorganic carbon, DIC) are needed to make CCMs energetically favorable for land plants.

## Results

### Validation of compensation point predictions and sensitivity analysis

**Figure 1.**
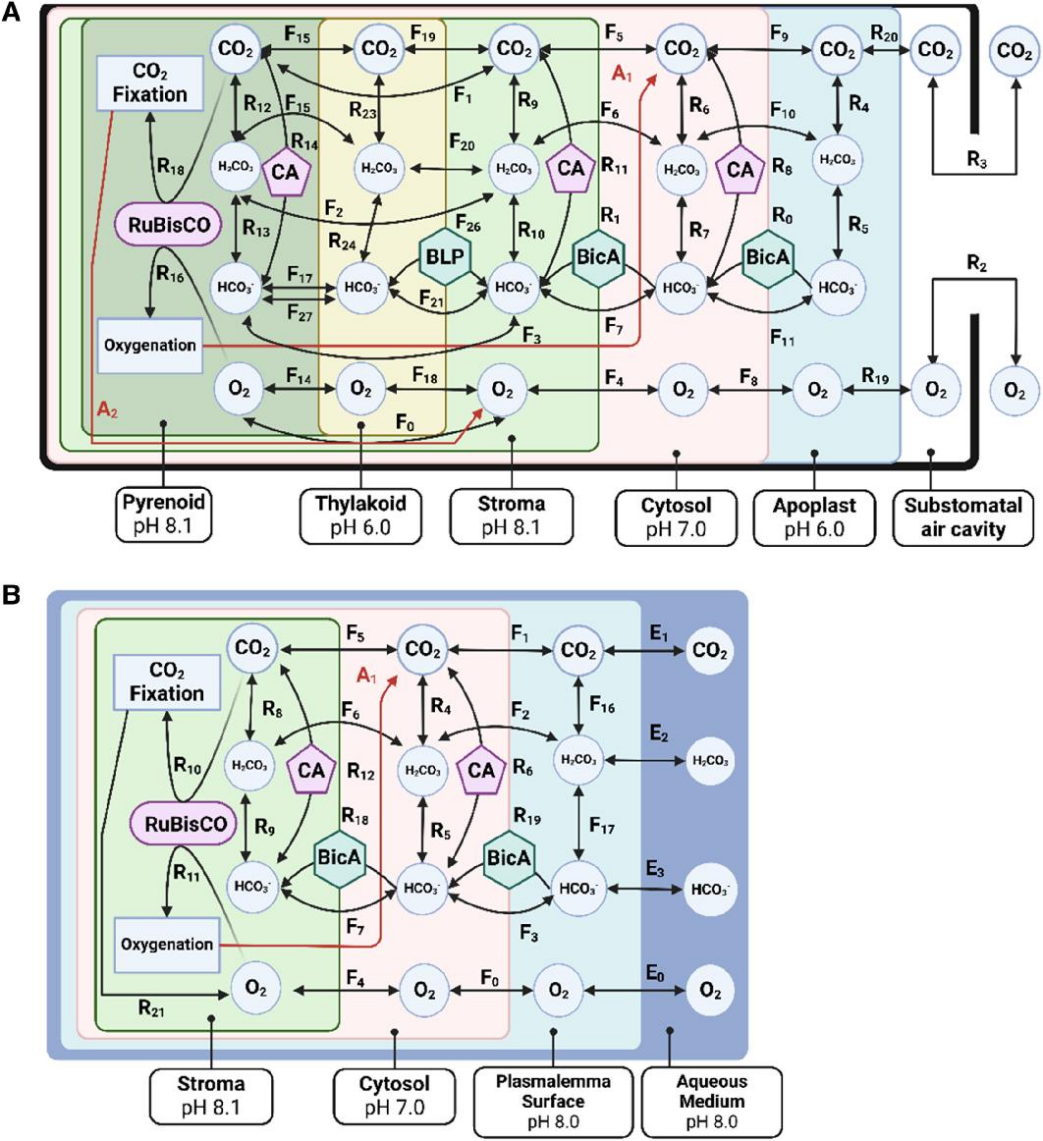
Diagrammatic representations of the models that were used in this study. **A)** a model of photosynthetic carbon assimilation in a land plant mesophyll cell containing a *C. reinhardtii* style PCCM and **B)** a model of an algal cell that does not contain a pyrenoid. CA refers to carbonic anhydrase, BLP refers to bestrophin-like proteins that serve as membrane channels for passive bicarbonate transport, and BicA is a cyanobacterial active bicarbonate transporter. In the *VCell* implementation of the model, some strongly linked steps are combined for the sake of numerical computability; some additional processes are not combined in the *VCell* model but grouped together for the sake of visual clarity (A1 to A2) and the individual reactions making them up can be found in the Supplementary Model Equations file. “F” is used to denote fluxes between compartments of the model. “R” is used to denote enzyme-catalyzed and spontaneous reactions as well as some processes (e.g. movement of gases into the substomatal space) where multiple processes have been aggregated into a single expression. Exact specifications for all flux equations used can be found in the publicly shared model implementations in *VCell* (see code and data availability statement). Note that BicA is only active in modeling scenarios where its activity is explicitly explored and only ever at the plasmalemma or the chloroplast membrane, not both. Also note that for the sake of numerical tractability, the carbonic-anhydrase-catalyzed interconversion of CO_2_ and HCO_3_ in the thylakoid in models featuring a CCM (R14) is localized to the pyrenoid but uses the pH value of the thylakoid; in the real biological system, the CA is inside the thylakoid tubules that penetrate into the pyrenoid. All DIC concentrations are still simulated independently for the pyrenoid and thylakoid. Additional model diagrams can be found in Supplementary Fig. S1. Equations for all fluxes can be found in the Supplementary Model Equations file.

The land plant and algal carbon assimilation models (see Fig. 1 and Supplementary Fig. S1 for model diagrams and Table 1 for model parameters) were validated by comparing a key estimated result (CO_2_ compensation point) with experimentally measured values from the literature. The CO_2_ compensation point is the external CO_2_ level at which net CO_2_ assimilation by a photosynthesizing organism is zero (i.e. carbon assimilation by rubisco is balanced out by CO_2_ losses to photorespiration and respiration in the light, denoted as *R*_L_). Low compensation points are also a defining feature of organisms with CCMs since they maintain net positive carbon assimilation at lower CO_2_ concentrations, making this a useful indicator of whether land plant and algal models with and without CCMs reasonably recreate the carbon assimilation dynamics of real systems.

**Table 1.** Model parameter definitions with source references and where applicable with notes on derivation

Name (symbol)	Value(s)	Units	Notes	Ref.
Diffusion coefficient of CO_2_ in water ($D_{CO_{2}}$)	1.88 × 10^3^	*μ*m^2^ s^−1^		(1, 2)
Diffusion coefficient of H_2_CO_3_ in water ($D_{H_{2}CO_{3}}$)	1.2 × 10^3^	*μ*m^2^ s^−1^	Assumed in Fei et al. (2022) to be identical to diffusion coefficient of acetic acid	(2, 3)
Diffusion coefficient of ${\mathrm{HCO}}_{3}^{-}$ in water ($D_{\mathrm{HC}O_{3}}$)	1.15 × 10^3^	*μ*m^2^ s^−1^		(2, 4)
Diffusion coefficient of O_2_ in water ($D_{O_{2}}$)	2.42 × 10^3^	*μ*m^2^ s^−1^		(5)
Membrane permeability to CO_2_ ($P_{CO_{2}}$)	3.50e−03; 3.20e−02	m s^−1^	Parameter scanned between reported values	(6, 7)
Membrane permeability to H_2_CO_3_ ($P_{H_{2}CO_{3}}$)	30	*μ*m s^−1^		(2)
Membrane permeability to ${\mathrm{HCO}}_{3}^{-}$ ($P_{\mathrm{HC}O_{3}}$)	0.05	0.05 *μ*m s^−1^		(8)
Membrane permeability to O_2_ ($P_{O_{2}}$)	75	cm s^−1^		(9)
Besotrophin-like channel-mediated permeability of thylakoid to ${\mathrm{HCO}}_{3}^{-}$ ($P_{\mathrm{HC}O_{3}/\mathrm{facilitated}}$)	1 × 10^−2^	m s^−1^		(2)
Chloroplast membrane permeability to ${\mathrm{HCO}}_{3}^{-}$ mediated by Low CO_2_-Inducible protein A	1 × 10^−8^	m s^−1^		(2)
Rate constant of spontaneous hydration of CO_2_ (Kf^hydration^)	6 × 10^−2^	s^−1^		(10)
Rate constant of spontaneous dehydration of H_2_CO_3_ (Kr^dehydration^)	2 × 10^1^	s^−1^		(10)
Rate constant of spontaneous deprotonation of H_2_CO_3_ (Kf^deprotonation^)	1 × 10^7^	s^−1^		(10)
Rate constant of spontaneous protonation of ${\mathrm{HCO}}_{3}^{-}$ (Kr^protonation^)	5 × 10^10^	M^−1^ s^−1^		(10)
Carbonic anhydrase ***k***_cat_ ($CA_{k_{\mathrm{cat}}}$)	0.3 × 10^6^	s^−1^		(11)
Carbonic anhydrase ***K***_m_ for CO_2_ (${\mathrm{CA}}_{CO_{2}}^{K_{m}}$)	1.5	mol m^−3^		(12)
Carbonic anhydrase ***K***_eq_ (***K***_eq_)	0.56 × 10^−6^	mol m^−3^		(13)
Carbonic anhydrase ***K***_m_ for HCO_3_ (${\mathrm{CA}}_{\mathrm{HC}O_{3}}^{K_{m}}$)	34	mol m^−3^		(13)
Carbonic anhydrase concentration in stroma ([CA]_str_)	270	*μ*M		(14)
Carbonic anhydrase concentration in cytosol ([CA]_cyt_)	135	*μ*M	Assumed to be half the stroma value	(14)
Carbonic anhydrase concentration in lumen ([CA]_lum_)	135	*μ*M	Assumed to be half the stroma value	(14)
Rubisco ***V***_max_ of carboxylation ($\mathrm{Rubisc}o^{V_{c}}$)	7600	*μ*mol L^−1^ s^−1^		(15)
Rubisco ***V***_max_ of oxygenation ($\mathrm{Rubisc}o^{V_{o}}$)	1596	*μ*mol L^−1^ s^−1^	Calculated from ratio of *k*_cat_ values of carboxylation and oxygenation.	(16, 17)
Rubisco ***K***_m_ O_2_ (${\mathrm{Rubisco}}_{O_{2}}^{K_{m}}$)	8.6	*μ*mol L^−1^		(18)
Rubisco ***K***_m_ CO_2_ (${\mathrm{Rubisco}}_{CO_{2}}^{K_{m}}$)	215	*μ*mol L^−1^		(18)
BicA ***V***_max_ ($V_{\mathrm{ma}x_{\mathrm{BicA}}}$)	1.85 × 10^−4^	mol m^−2^ s^−1^	Parameter scanned	(19)
BicA ***K***_m_${\mathrm{HCO}}_{3}^{-}$ ($K_{m_{\mathrm{BicA}}}$)	0.217	mol m^−3^		(19)
Stomatal conductance (StomatalConductance)	0.4375	mol m^−2^ s^−1^		(20)
Atmospheric concentration of CO_2_ ([CO_2_]_ext_)	412	ppm		Assumed
Atmospheric concentration of O_2_ ([O_2_]_ext_)	0.21	Partial pressure		(21)
Thickness of cell wall in angiosperms (Thickness_C3_)	0.32	*μ*m		(14, 22)
Thickness of cell wall in bryophytes (Thickness_hornwort_)	1.6	*μ*m		(23)
Effective porosity of C3 plant cell wall (EffectivePorosity_C3_)	0.2	Unitless		(14)
Effective porosity of algal cell wall (EffectivePorosity_algae_)	0.2	Unitless	Assumed to be same as C3 plant	Assumed
Effective porosity of hornwort cell wall (EffectivePorosity_hornwort_)	0.0001		Parameter scanned	Calculated (23)
**Ratio_mesophyll area_**	15	Unitless		Slaton and Smith (2002)
Thickness of unstirred boundary layer in algal model (Thickness_boundary_)	0.32	*μ*m	Assumed to be the same as cell wall thickness	Assumed
Thickness of unstirred apoplast water layer in land plant models (Thickness_apoplast_)	0.32	*μ*m	Assumed to be the same as cell wall thickness	Assumed
Permeability of pyrenoid starch sheath to DIC (Pyrenoid_multiplier_)	0.1 * $P_{CO_{2}}$	*μ*m s^−1^	From range of permeabilities that allow effective carbon concentration in modeling done by Hopkinson et al. (2011)	(2)
Permeability of pyrenoid starch sheath to oxygen (Pyrenoid_multiplier_)	0.1 * $P_{O_{2}}$	*μ*m s^−1^	Assumed to behave similarly to DIC	Assumed
Radius of pyrenoid (***r***_pyrenoid_)	1.0	*μ*m		(2)
Radius of thylakoid (***r***_thylakoid_)	0.5	*μ*m	Multiplied by 10× to account for simpler thylakoid architecture	(2)
Height of thylakoid (height_thylakoid_)	4	*μ*m		Assumed
Radius of chloroplast (***r***_chloroplast_)	4.63	*μ*m	Calculated from stroma volume fraction and assuming spherical geometry	(14)
Radius of cytosol (***r***_cytosol_)	8.77	*μ*m	Assuming spherical geometry	(24)
Radius of plasmalemma surface (***r***_plasmalemma surface_)	9.23	*μ*m	Calculated from the radius of cytosol, cell wall thickness, and assumed apoplast water thickness	Calculated
Radius of substomatal space in land plant model (***r***_substomatal **space**_**)**	11.63	*μ*m		(14)
Proportion of cell wall adjacent to intercellular airspace in land plant (IAS_adjacent_)	0.5	Unitless		(25)
pH of land plant apoplast (pH_apoplast_)	6.0	pH		(26)
pH of ocean water (pH_ocean water_)	8.0	pH		(27)
pH of cytosol (pH_cytosol_)	7.2	pH		Calculated (28)
pH of stroma (pH_stroma_)	8.0	pH		(29)
pH of lumen (pH_lumen_)	6.0	pH		(29)
Viscosity of cytosol (Visc_cytosol_)	2	Unitless		(14, 30, 31, 32)
Viscosity of stroma (Visc_stroma_)	10	Unitless		(14, 30, 32)
Viscosity of thylakoid (Visc_thylakoid_)	10	Unitless	Assumed to be the same as stroma	(14)
Viscosity of pyrenoid (Visc_pyrenoid_)	10	Unitless	Assumed to be the same as stroma	

When parameters were derived from the parameterization of a previous modeling study, both the modeling study and the original literature reference for the parameter are cited. References in “Ref.” column: (1) Mazarei and Sandall (1980); (2) Fei et al. (2022); (3) Xiang and Anderson (1994); (4) Walker et al. (1980); (5) Bentley and Pittman (1997); (6) Gutknecht et al. (1977) (7) Missner et al. (2008); (8) Hopkinson et al. (2011); (9) Widomska et al. (2007); (10) Mitchell et al. (2010); (11) Larsson et al. (1997); (12) Pocker and Ng (1973); (13) Pocker and Miksch (1978); (14) McGrath and Long (2014); (15) Bernacchi et al. (2005); (16) Badger and Andrews (1974); (17) Farquhar et al. (1980); (18) von Caemmerer (2000); (19) Price et al. (2004); (20) Bernacchi et al. (2006); (21) Kump (2008); (22) Pritchard et al. (1986); (23) Flexas et al. (2021); (24) Ouk et al. (2020); (25) Slaton and Smith (2002); (26) Yu et al. (2000); (27) Jiang et al. (2019); (28) Felle (2001); (29) Kramer et al. (1999); (30) Tholen and Zhu (2011); (31) Scalettar et al. (1991); and (32) Köhler et al. (2000).

**Figure 2.**
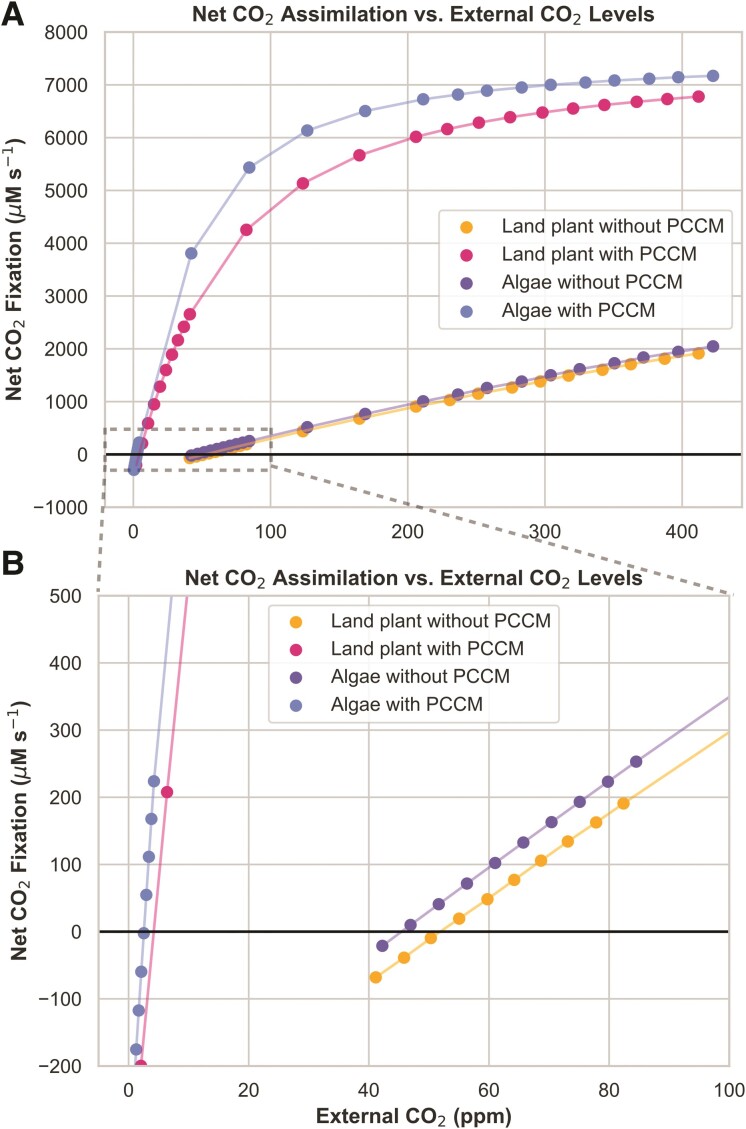
Net CO_2_ assimilation versus external CO_2_ concentrations in parts per million (ppm) in carbon assimilation models. The point at which net CO_2_ assimilation is zero defines the compensation point. **A)** The full range of net CO_2_ fixation and external CO_2_ concentrations and **B)** a zoomed-in panel showing the point at which each curve reaches a net CO_2_ fixation of 0 (i.e. the compensation point).

As shown in Fig. 2 and Table 2 (original data in Supplementary Tables S1 to S4), the models with CCMs have substantially lower compensation points than the models lacking CCMs. Moreover, as shown in Table 2, these estimated compensation point values fall within the ranges of values reported in the literature for angiosperm land plants and algae with and without CCMs (Table 2). Note that the reported compensation points of hornworts with pyrenoids (11 to 13 ppm) are lower than those of closely related C3 liverworts but higher than typical estimates for C4 plants and pyrenoid-containing algae (Villarreal and Renner 2012).

**Table 2.** Predicted compensation points for different models from the present study compared with reference values from the literature

Model	Compensation point (ppm CO_2_)	Reference values (ppm CO_2_)	Measurement references
Land plant with CCM	4.2	1.3; 4.3; 0.7 to 9.0	Fladung and Hesselbach (1987), Lee et al. (2022)
Land plant without CCM	51.9	48; 57; 48.2 to 53.4; 65 to 100	Fladung and Hesselbach (1987), Tolbert et al. (1995), Peixoto et al. (2021)
Algal model with CCM	2.7	0.75 to 2.5; 6.0	Coleman and Colman (1980), Raven et al. (1982)
Algal model without CCM	44.6	43.5 to 58; 64.5	Raven et al. (1982), Steensma et al. (2023)

Reference column numbers refer to their numbering in the bibliography.

Several other predicted quantities align well with values reported in the literature. Assuming the lipid permeability value reported in Gutknecht et al. (1977) and an external CO_2_ concentration of 412 ppm, the C3 land plant model lacking an added CCM predicts a quantum yield of 0.068. This is almost certainly an overestimate due to the omission of respiration in the light of this model. Quantum yields of ∼0.05 for both C3 and C4 plants, taken from Ehleringer and Björkman (1977), are commonly cited in the literature but were measured at much lower atmospheric CO_2_ concentrations than we see in the present day. For C3 plants, a study of tomatoes which measured quantum yields under different levels of nitrogen availability and light found quantum yields ranging from 0.0538 to 0.0758 (Moriwaki et al. 2019). Under full sunlight and nitrogen-replete conditions, which we believe most closely match our model setup, they measured a quantum yield of 0.0675 (Moriwaki et al. 2019). In contrast, the net photosynthetic rate and, when light levels are held constant, the quantum yield of photosynthesis of C4 plants does not vary strongly with atmospheric CO_2_ levels (Ehleringer and Björkman 1977). We do not vary stomatal conductance in our with-CCM land plant models, but at external CO_2_ levels that result in C_i_ similar to that reported for C4 plants, our model predicts a quantum yield of 0.055, compared to the value of 0.0534 measured in Ehleringer and Björkman (1977). From measurements in the C3 plant *Camelina sativa*, we can estimate a 95% confidence interval of [0.25, 0.40] for rubisco saturation under long-day conditions (Xu et al. 2023). For comparison, this study's land plant model lacking a CCM predicts a value of 0.252 at atmospheric CO_2_ and assuming the lipid membrane permeability reported in Gutknecht et al. (1977). To summarize, we believe the combination of compensation point, quantum yield, and rubisco saturation validations detailed above provide confidence that the models described in this study reproduce the behavior of the real systems they describe.

**Figure 3.**
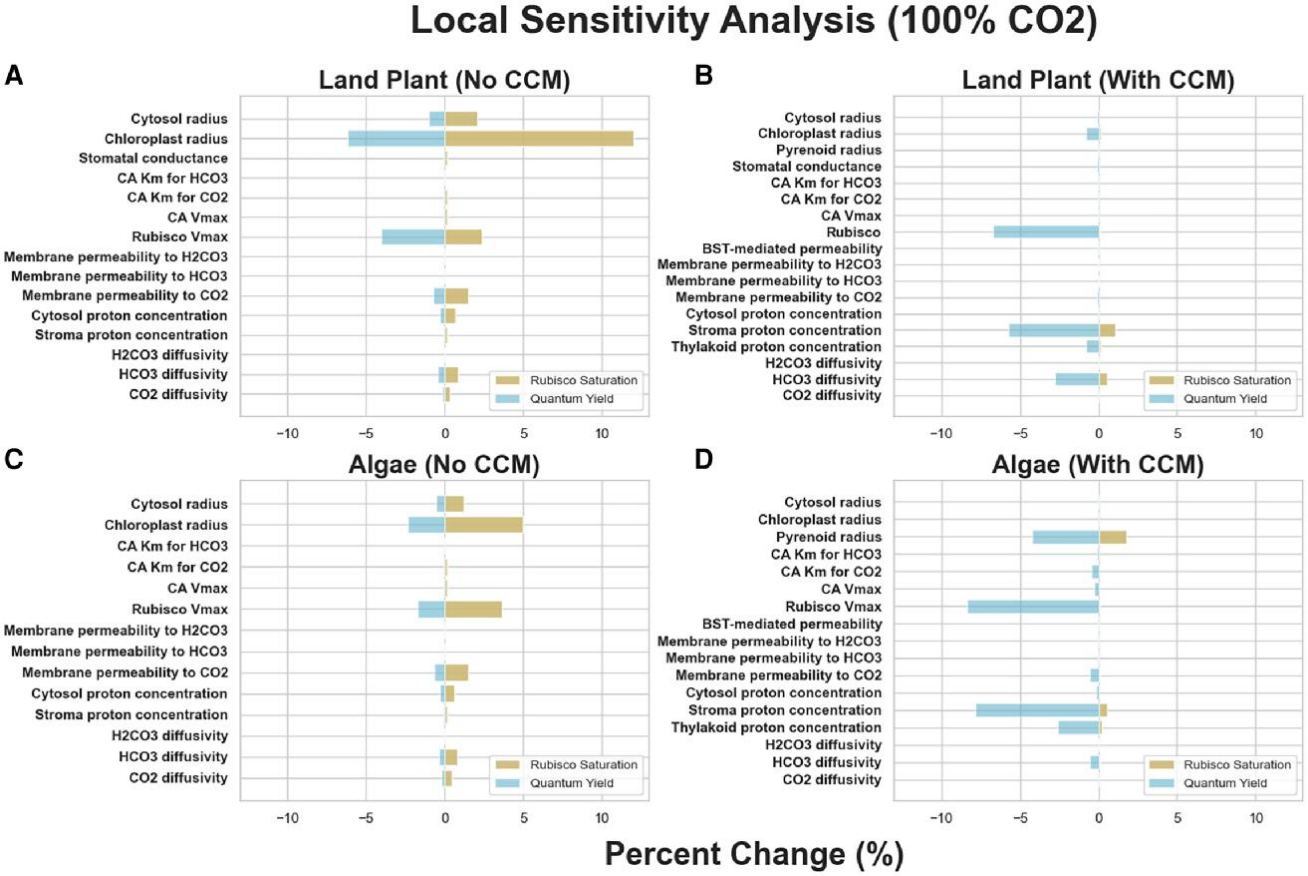
Sensitivity analysis results models presented in this study. **A)** The land plant model lacking a CCM, **B)** the algal model lacking a CCM, **C)** the land plant model with a CCM, and **D)** the algal model with a CCM. Gold bars indicate the absolute % change of quantum yield resulting from a 10% change in the indicated parameter, and blue bars represent the same for rubisco saturation. Values are averages of the absolute % change resulting from a 10% increase and decrease; non-averaged values are shown in **Supplementary Table S21**. Local sensitivities were calculated at an external CO_2_ concentration of 412 ppm. For both land plant models, increasing the cytosol radius by 10% resulted in problems with solving the systems numerically, so the cytosol radius was increased by 1% instead and, assuming a linear relationship between the size of radius increase and the change in rubisco saturation and quantum yield, multiplied by 10 to get the values shown in **A)** and **B)**.

The sensitivity analysis results given in Fig. 3 (original data and calculations in Supplementary Tables S5 to S9) show that simulated net CO_2_ assimilation and quantum yield values from the land plant models are relatively robust to local variations in all parameters, providing us with confidence that these results are not merely the result of a very particular selection of parameters. In both the land plant and algal models without PCCMs, rubisco *V*_max_, cell and chloroplast radii, and membrane permeability to CO_2_ are the most influential determinants of net CO_2_ assimilation and quantum yield. In the land plant model, stomatal conductance also stands out. The addition of a PCCM reduces the sensitivity of net CO_2_ assimilation to changes in any input parameter but increases the sensitivity of the predicted quantum yield to input parameter values. The local stability of our results to perturbations in key parameters is comparable with previous studies, being more variable than the models presented in Fei et al. (2022), which spatially modeled a smaller system (algal chloroplasts), and less variable than the models presented in McGrath and Long (2014), which modeled land plant CO_2_ assimilation at a similar scale. Looking at the local stability of our results at lower (50%) external CO_2_ levels, we see qualitatively similar results (Supplementary Fig. S2 and Supplementary Table S10).

We also characterized the sensitivity of our modeling results to the spatial resolution of the numerical simulations. Our results (Supplementary Figs. S3 and S4) show that rubisco saturation—the percentage of maximum rubisco activity achieved—and quantum yield in an algal model lacking a CCM are robust to the simulation resolution. From model predictions made at spatial resolutions of 0.91 and 0.61 *μ*m, we can estimate that the sensitivity of our rubisco saturation predictions to a 10% change in the number of simulated voxels is approximately 0.10% and the sensitivity of our quantum yield predictions is approximately 0.12%. These values are small compared with other sources of uncertainty in the model (Fig. 3). Increasing the resolution all the way down to 0.32 *μ*m in the algal model with a CCM, well beyond what could feasibly be done given the amount of parameter exploration done in this study, does result in noticeable changes in pyrenoid [CO_2_] and [${\mathrm{HCO}}_{3}^{+}$], resulting in small increases in rubisco saturation and small decreases in quantum yield (Supplementary Figs. S5 and S6). To test whether the simplification of tubule geometry made in this study, which features a smaller number of tubules of larger radius, with Fei et al. (2022), which features a larger number of tubules of smaller radius, we multiplied all permeabilities and conductivities acting across the stroma:thylakoid and thylakoid:pyrenoid matrix interfaces in the algal CCM model by 10 to more closely approximate the surface area:volume ratio in (Fei et al. 2022). This only results in a 6.1% change in the predicted quantum yield of photosynthesis and a 3.9% change in rubisco saturation, suggesting that this geometric simplification does not have a meaningful effect on the conclusions of this study.

### Efficiency of chloroplast membrane bicarbonate channel is strongly dependent on assumed permeability of chloroplast membrane to CO_2_

Previous studies (Price et al. 2010; McGrath and Long 2014) have suggested that the incorporation of bicarbonate transporters into the chloroplast membrane of a land plant could improve net fixation and/or the efficiency of carbon assimilation and that this could represent a reasonable intermediate stage in a broader biotechnological effort to implement a full CCM in a land plant. Modeling studies on CCM systems typically assume the lipid membrane permeability of 0.35 cm/s, which was experimentally measured and reported in Gutknecht et al. (1977). However, there is substantial uncertainty as to the value of the parameter, with experimental estimates ranging over many orders of magnitude (Evans et al. 2009). The permeability may be as much as an order of magnitude higher than the Gutknecht *et al.* value, as reported in Missner et al. (2008). We hypothesized that the apparent favorability of employing a chloroplast membrane bicarbonate pump may be highly sensitive to the assumed chloroplast membrane CO_2_ permeability.

**Figure 4.**
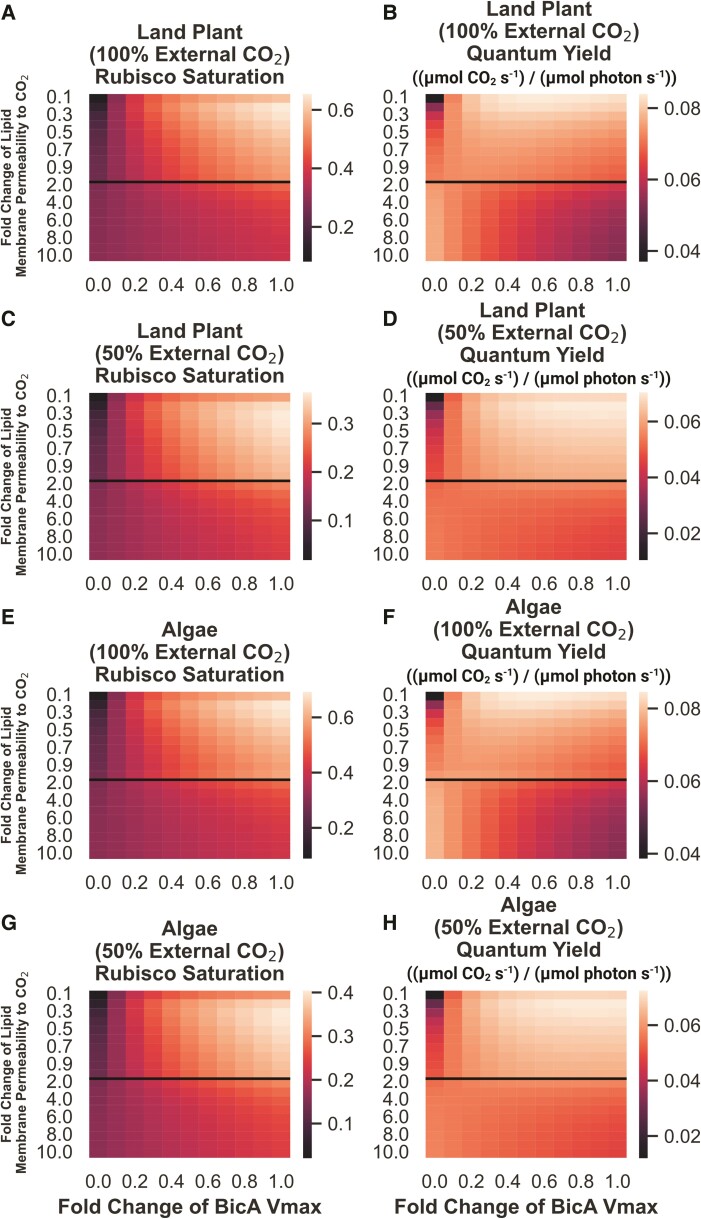
Rubisco saturation and quantum yield of land plant and algal models of CO_2_ assimilation under 100% and 50% external CO_2_ levels, as a function of lipid membrane permeability to CO_2_ and BicA bicarbonate transporter *V*_max_. Fold change of lipid membrane permeability is relative to the value reported in Gutknecht et al. (1977) and applies to all lipid bilayers. In the models investigated, BicA activity is only present at the chloroplast, and not the plasmalemma, surface. **A)** Predicted rubisco saturation of a land plant model under 100% external CO_2_. **B)** Predicted quantum yield of a land plant model under 100% external CO_2_. **C)** Predicted rubisco saturation of a land plant model under 50% external CO_2_. **D)** Predicted quantum yield of a land plant model under 50% external CO_2_. **E)** Predicted rubisco saturation of an algal model under 100% external CO_2_. **F)** Predicted quantum yield of an algal model under 100% external CO_2_. **G)** Predicted rubisco saturation of an algal model under 50% external CO_2_. **H)** Predicted quantum yield of an algal model under 50% external CO_2_. The black lines in each plot indicate the (Gutknecht et al. 1977) value for lipid bilayer permeability to CO_2_ as well as a transition in the *y*-axis from increments of 0.1× to 1× fold changes.

To test this hypothesis, we performed a parameter exploration from an order of magnitude lower than the widely cited (Gutknecht et al. 1977) value up to the (Missner et al. 2008) value in both land plant and algal systems, calculating net fixation as well as ATP/CO_2_ and light-use efficiency, as shown in Fig. 4 (original data in Supplementary Tables S11 and S12).

These results show that the light use efficiency of a chloroplast membrane bicarbonate transporter is highly sensitive to the value of the chloroplast envelope's permeability to CO_2_, with a large range of permeabilities resulting in 2× more ATP usage per unit of CO_2_ fixed. In the land plant model, we see increases in both rubisco saturation and quantum yield as BicA pumping activity increases when lipid membrane permeability values are equivalent to or below that reported in Gutknecht et al. (1977) (Fig. 4, A and B). At permeabilities higher than this, increased BicA activity actually decreases quantum yield, though net fixation still increases (Fig. 4, A and B). We see a similar picture in the algal model (Fig. 4, E and F), suggesting that the differences in DIC form, concentration, and diffusivity do not greatly impact the sensitivity of this strategy to the specific value of lipid membrane permeability to CO_2_. The decrease in quantum yield in models with high lipid membrane permeability to CO_2_ is driven by increased leakage of CO_2_ from the chloroplast back into the cytosol after it interconverts with the bicarbonate just pumped by BicA (shown as flux *V*_15_ in Fig. 1). As lipid membranes become more permeable to CO_2_, its tendency to escape the chloroplast before being fixed by rubisco increases. Lowering the external CO_2_ concentration does, however, change the energy efficiency penalty of increased BicA activity substantially (Fig. 4, C, D, G, and H). Even at higher lipid membrane permeability values, we see only minimal decreases in quantum yield with increased BicA bicarbonate pumping.

### Efficiency of a plasmalemma bicarbonate channel is strongly dependent on external DIC levels and limited by the rate of equilibration between CO_2_ and bicarbonate

**Figure 5.**
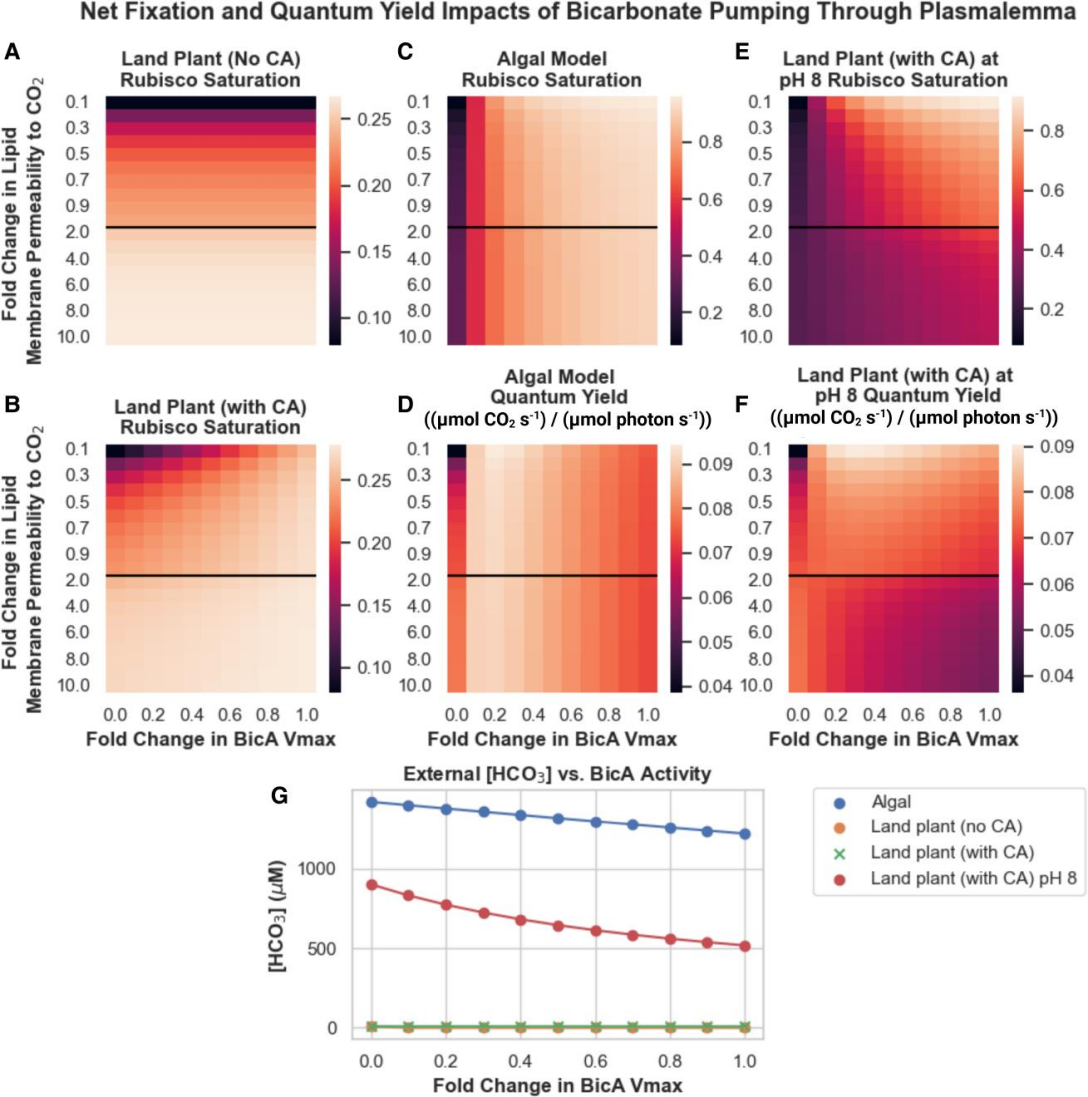
Predicted rubisco saturation and quantum yield in land plant and algal models with a BicA bicarbonate pump present in the plasmalemma membrane, as a function of assumed lipid membrane permeability to CO_2_ and BicA *V*_max_. Fold change of lipid membrane permeability is relative to the value reported in (Gutknecht et al. 1977) and applies to all lipid bilayers. In the models investigated, BicA activity is only present at the plasmalemma, and not the chloroplast, surface. **A)** Predicted rubisco saturation of a land plant model lacking an apoplastic CA. **B)** Predicted rubisco saturation of a land plant model with an apoplastic CA. **C)** Predicted rubisco saturation of an algal model. **D)** Predicted quantum yield of an algal model. **E)** Predicted rubisco saturation of a land plant model with an apoplastic CA and an apoplast pH of 8. **F)** Predicted quantum yield of a land plant model with an apoplastic CA and an apoplast pH of 8. **G)** Steady-state external [${\mathrm{HCO}}_{3}^{-}$] for different models as a function of plasmalemma BicA *V*_max_. The black lines in each plot indicate the (Gutknecht et al. 1977) value for lipid bilayer permeability to CO_2_ as well as a transition in the *y*-axis from increments of 0.1× to 1× fold changes. All values were calculated at an external environmental CO_2_ concentration of 412 ppm. CA, carbonic anhydrase.

We found that although the strategy of pumping bicarbonate from the cytosol to the chloroplast may incur substantial energy costs, implementing a bicarbonate pump at the plasmalemma may be more effective. This makes sense considering that in aqueous systems at near-neutral pH, most of the DIC in the system is in the form of bicarbonate. We incorporated a plasmalemma bicarbonate transporter and explored the efficiency of such a system across different external DIC concentrations and activities of the transporter in both algal and land plant systems (Fig. 5; original data in Supplementary Tables S13 to S16).

In the land plant model, the plasmalemma bicarbonate pump is not an effective means of increasing either net fixation or energy efficiency. As anticipated, the pump does work in the algal case (Fig. 5). The key difference appears to be that the external environment in the algal system, which is suffused with bicarbonate ions, can maintain reasonably high steady-state concentrations in the vicinity of the cell to support the bicarbonate pumping activity (Fig. 5, C and D). In contrast, in the land plant system, all dissolved bicarbonate available to the cell must first enter the system as CO_2_ in the intercellular airspace, dissolve into the water in the apoplast, and then spontaneously hydrate to H_2_CO_3_ and deprotonate into bicarbonate. Although the protonation/deprotonation between H_2_CO_3_ is extremely fast, the hydration/dehydration is not (first-order rate constant of hydration of CO_2_ to H_2_CO_3_ is 6 × 10^−2^ s^−1^; Mitchell et al. 2010). The result is an almost instantaneous depletion of the ${\mathrm{HCO}}_{3}^{-}$ concentration in the apoplast space, with insufficient spontaneous hydration flux to replenish it (Fig. 5G). Adding CA activity to the apoplast allows for much faster regeneration of the external ${\mathrm{HCO}}_{3}^{-}$ concentration, allowing BicA to impact rubisco saturation (Fig. 5, A and B). However, the pH of the apoplast, although variable, tends to be slight to moderately acidic (Yu et al. 2000), resulting in low ${\mathrm{HCO}}_{3}^{-}$ concentrations in the land plant model even with the apoplast CA included (Fig. 5G). It is only when the apoplast pH is made substantially more basic (pH of 8) and a CA is included that the land plant model can replicate the algal model's rubisco saturation and quantum yield gains by using a plasmalemma bicarbonate pump (Fig. 5, E and F).

### PCCM integration results in greater marginal cost of CO_2_ fixation improvements in land plants vs. algal systems and switches from decreasing to increasing light-use efficiency around a C_i_ typical of C4 plants

**Figure 6.**
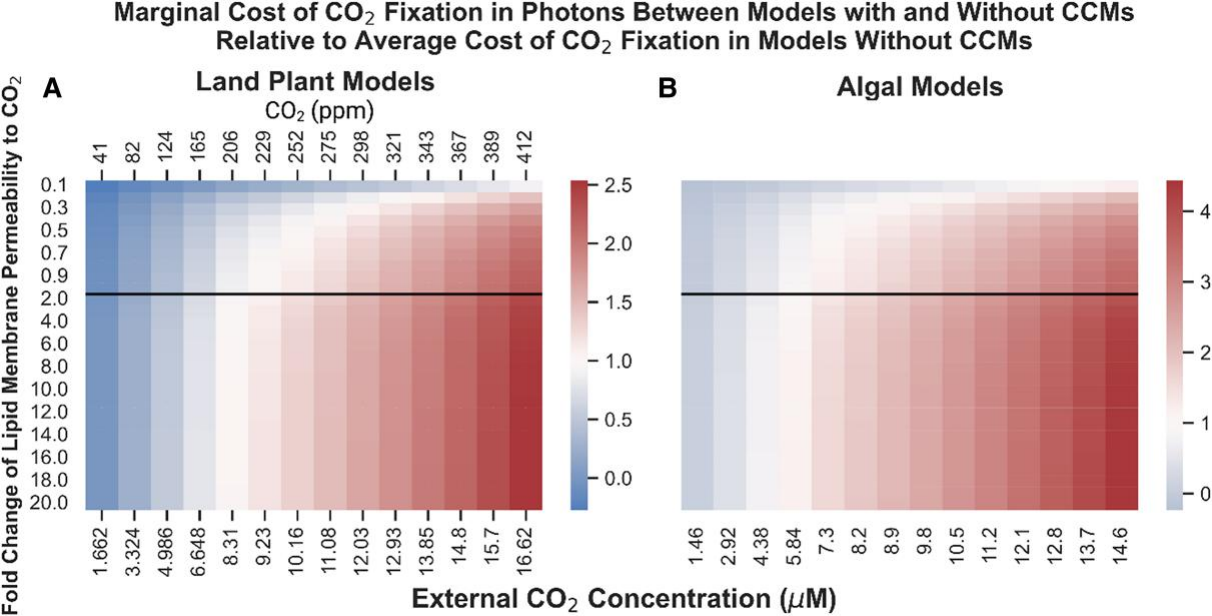
The ratio of the marginal cost in photons of one unit of net CO_2_ fixation resulting from adding a PCCM relative to the average cost of fixing one molecule of CO_2_ in those same models without CCMs, as a function of lipid membrane permeability and external CO_2_ concentrations. **A)** Results in land plant models with and without PCCM. **B)** Results in algal models with and without PCCM. Fold change of lipid membrane permeability is relative to the value reported in (Gutknecht et al. 1977). Blue indicates that for a given lipid membrane permeability/external CO_2_ concentration combination, the model containing a CCM has a lower marginal cost of CO_2_ fixation—i.e. is more light-efficient—than the average cost of CO_2_ fixation in the model lacking a CCM. Red indicates that for a given parameterization, the model containing a CCM has a higher marginal cost of CO_2_ fixation than the average cost of CO_2_ fixation in its CCM-lacking counterpart. The black lines in each plot indicate the (Gutknecht et al. 1977) value for lipid bilayer permeability to CO_2_ as well as a transition in the *y*-axis from increments of 0.1 to 1 in the *x*-fold changes. PPM, parts per million CO_2_.

We compared the energy-use efficiency of PCCM integration by comparing the predicted cost in photons of fixing CO_2_ molecule in 4 different models: (i) a land plant model with a PCCM, (ii) a land plant model without a PCCM, (iii) an algal model with a PCCM, and (iv) an algal model without a PCCM. By dividing the increase in net CO_2_ fixation in Models (i) and (iii) relative to Models (ii) and (iv), we estimated the marginal cost of in photons of fixing an additional CO_2_ molecule using a PCCM in our land plant and models (Fig. 6; original data in Supplementary Tables S17 to S20). As we observed when examining the efficiency of the plasmalemma and chloroplast envelope BicA bicarbonate pumps, the assumed permeability of lipid membranes can have an impact on efficiency; in this case, however, the relative marginal cost values do not change dramatically between an assumed permeability equivalent to that used in previous studies (1.0 in Fig. 6, A and B) and the higher value closer to that reported in Missner et al. (2008).

In the algal models, the use of the PCCM appears to only become marginally efficient with respect to light usage below an external [CO_2_] of 4.38 *µ*M. In contrast, the CCM is efficient in the land plant model at and below a substomatal [CO_2_] of ∼164 or 6.65 *µ*M ppm. At such substomatal [CO_2_] levels, the predicted lumen CA fluxes in the land plant model are on the order of ∼1.0 × 10^−16^ mol s^−1^, similar to values predicted for a functional PCCM in Fei et al. (2022).

### As cell wall thickness increases and cell wall effective porosity decreases, PCCMs become more favorable in land plant models

**Figure 7.**
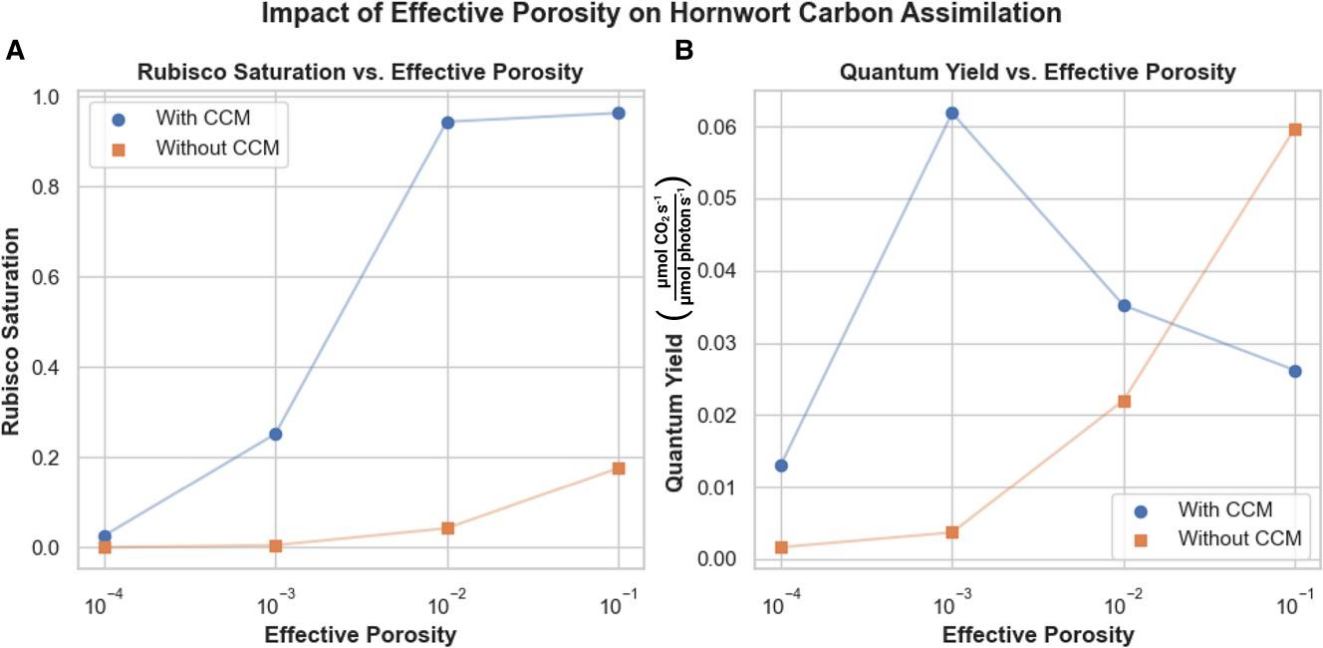
Effects of effective porosity on predicted photosynthetic efficiency in a simulated land plant model. Rubisco saturation **A)** and quantum yield **B)** with varying effective porosity values. All values were calculated at an external CO_2_ concentration of 412 ppm (parts per million). Blue points/lines represent predicted rubisco saturation or quantum yield in models including a PCCM; orange points/lines represent predicted saturation or quantum yield in models not including a PCCM.

Given the findings regarding PCCMs in land plants highlighted above, it is interesting that many species of hornworts have pyrenoids—are there any meaningful biophysical differences between hornworts and other land plants that could explain these differences? As highlighted in (Meyer et al. 2008; Flexas et al. 2021) hornworts and other bryophytes have cell walls that are both substantially thicker and less porous compared to other land plants. From the mesophyll conductance values reported for angiosperms and bryophytes reported in Meyer et al. (2008) and Flexas et al. (2021), and with the assumption that other internal resistances to CO_2_ diffusion are similar between bryophytes and embryophytes, we can estimate that the effective porosity of a bryophyte like a hornwort must be on the order of 4 orders of magnitude smaller than in a typical C3 angiosperm. We explore parameters within this range of possible porosity values and across multiple external CO_2_ concentrations (Fig. 7; original data in Supplementary Tables S21 and S22).

Below effective porosities on the order of 10^−1^, which fall in the range we would expect of angiosperms, our model shows that the plant struggles to fix CO_2_ without a CCM. With a PCCM, however, the model can achieve some level of net CO_2_ fixation all the way down to effective porosities of 10^−3^. Below porosities of 10^−3^, we do not observe net CO_2_ fixation in the model without a PCCM, and at a porosity of 10^−4^, both models with and without PCCMs struggle to fix carbon. In terms of light-use efficiency, the model with a PCCM achieves a greater quantum yield of photosynthesis than the model without a PCCM below effective porosities of 10^−2^.

## Discussion

We initially hypothesized that the conspicuous absence of biophysical CCMs in almost all land plant lineages, in contrast to algae where they are widespread (Raven et al. 2005), may be the result of the lower efficiency of such systems in land plants relative to algae, and that this results from their different biophysical contexts. To our surprise, we found that PCCMs appear to result in qualitatively similar improvements in quantum yield and net CO_2_ assimilation in land plant and algal models. In the algal model, the fact that the addition of a PCCM does not result in efficiency gains until relatively low external DIC levels are reached is surprising, given that *Chlamydomonas reinhardtii* cells appear to concentrate carbon even at recent “air-level”—approximately 330 ppm—CO_2_ concentrations (Badger et al. 1980). This implies that algae may routinely run their CCMs even when this incurs a quantum yield penalty. In contrast, the intercellular CO_2_ concentration at which the CCM improves quantum yield in the land plant model (∼164 ppm) is similar to, though slightly higher than, reported estimates of C_i_ in C4 plants under laboratory, greenhouse, and field conditions (Bunce 2005). Previous work has described the evolutionary history of C4 photosynthesis (Sage et al. 2018) and identified certain anatomical features—namely, Kranz anatomy—and environmental factors such as hot, arid conditions that lead to increased transpirational water loss and factors such as water-use efficiency as key predictors of C4 emergence. If the estimated quantum yield gains resulting from the introduction of a biophysical CCM to a land plant in this study apply to biochemical CCMs like C4 and CAM photosynthesis, this may represent an additional evolutionary driver toward such systems.

Hornworts are the only land plant lineage that has evolved a biophysical CCM and they have done so multiple times (Villarreal and Renner 2012). Hornworts, as well as some other bryophytes, are noteworthy for having substantially slower gas exchange between their surroundings and their photosynthetic tissues when compared with vascular land plants (Meyer et al. 2008). Our results show that in a land plant with the low effective cell wall porosities we might expect given their extremely poor gas exchange characteristics, the use of a CCM becomes necessary to achieve net CO_2_ fixation, which would impose a strong selective pressure for adopting one. The fact that hornworts represent the earliest-diverging extant branch of the land plants, and therefore may have maintained the genes and regulatory networks necessary to adopt a PCCM, may explain why this biophysical CCM strategy has been adopted by hornworts and not other land plants growing in conditions where biochemical CCMS have been selected for. We should note that in the models presented in this study, at effective porosities below 10^−3^, only single-digit values of rubisco saturation are achieved even with a biophysical CCM present and active, which may not be sufficient for viability, especially since we do not have or include estimates of respiration in the light in the models. This is despite the fact that mesophyll conductance to CO_2_ in hornworts, which we are using effective porosity as a proxy for in this study, has been measured to be 4-to-5 orders of magnitude lower than in angiosperms (Flexas et al. 2021). This suggests that our model underestimates the strength of the hornwort CCM or otherwise does not properly describe some aspects of hornwort CO_2_ assimilation. The ratio of chloroplast-to-thallus surface area has not been explored in our modeling but was found in a previous study to be a potentially important determinant of hornwort mesophyll conductance (Carriquí et al. 2019). Future work might aim to incorporate an exploration of chloroplast position and surface area to better account for this in the modeling.

These results shed light on potential challenges associated with improving crop productivity via the introduction of biophysical CCMs. The specific value chosen for the permeability of lipid bilayers to CO_2_ has a large effect on the predicted energy efficiency of our models, with values higher than those used in previous modeling studies (McGrath and Long 2014; Fei et al. 2022) but within the range of previously reported literature values (Gutknecht et al. 1977; Missner et al. 2008) resulting in qualitatively different conclusions. We see this in our consideration of BicA-mediated ${\mathrm{HCO}}_{3}^{-}$ pumping, which had been previously flagged as a promising intermediate step in introducing a biophysical CCM to a C3 plant (Price et al. 2010; McGrath and Long 2014). As noted in Fei et al. (2022), barriers to CO_2_ diffusion form a key component of known functional CCMs, so the finding that the chloroplast membrane may provide enough of a diffusion barrier for the transport of ${\mathrm{HCO}}_{3}^{-}$ into the stroma and subsequent conversion to CO_2_ to meaningfully improve net fixation and carbon assimilatory efficiency was surprising. Our results show that at or below the permeability reported in (Gutknecht et al. 1977), which is used in other modeling studies, increasing BicA pumping activity leads to improvements in quantum yield, indicating more efficient CO_2_ fixation with respect to light use. However, above this value, we see uniform decreases in quantum yield with increased BicA activity. Net CO_2_ fixation increases with BicA pumping in all cases; therefore, in situations where light is abundant relative to CO_2_, this decrease in efficiency may not impact plant fitness. However, recent modeling work suggests that *J*_max_, the maximum rate of ribulose-1,5-bisphosphate (RuBP) regeneration enabled by photosynthetic electron transport, is more limiting to crop yield than limits to the maximum rate of carboxylation (*V*_max_ of rubisco carboxylation) under the projected elevated atmospheric CO_2_ levels of 2,050 and 2,100 (He and Matthews 2023). In this study, improved quantum yields correspond to a combination of (i) lower expenditures of ATP for each CO_2_ molecule fixed and (ii) a more favorable ATP/NADPH ratio needed for fixation, resulting in less energy loss from the use of cyclic electron flow (CEF) during ATP/NADPH rebalancing (Walker et al. 2020). Under conditions of *J*_max_ limitations, differences in quantum yield may become a critical factor in determining yield, making the sensitivity of quantum yield in this and other studies to assumed lipid bilayer permeability to CO_2_ a matter of critical importance.

Interestingly, previous studies in this area (McGrath and Long 2014; Fei et al. 2022) have performed sensitivity analyses that include this permeability as a surveyed parameter and its modeled effect is small compared to other parameters. These small local sensitivity values are estimated by observing the change in an output value like light-saturated CO_2_ assimilation with a ±10% change in the permeability parameter. This ignores the fact that the uncertainty in this value is in the range of at least an order of magnitude (Evans et al. 2009), and so despite low local sensitivity, the overall change that can result from varying it within reasonable bounds is substantial. The substantial uncertainty in this critical parameter could be reined in by future experimental measurements, though this will still be complicated by the potentially large variation between different plant systems, dynamic remodeling of lipid bilayers in response to developmental and environmental cues, etc. In the absence of well-defined values for this parameter, we encourage future groups modeling such systems to explore a range of values and to characterize the robustness of their conclusions to its variation.

In the near-neutral or slightly basic conditions that most photosynthetic organisms in aqueous environments find themselves in, ${\mathrm{HCO}}_{3}^{-}$ represents the primary form of DIC in their surroundings. Due to the impermeability of lipid bilayers to passive diffusion of ${\mathrm{HCO}}_{3}^{-}$, the use of this pool of DIC requires organisms to employ an active transport mechanism (e.g. cyanobacterial ${\mathrm{HCO}}_{3}^{-}$ pumps like BicA; Price et al. 2004) to move it from the extracellular to the intracellular space, which may often make sense due to the sheer quantity of DIC that is present in the environment. Although land plants ultimately obtain CO_2_ from the atmosphere, this CO_2_ must dissolve into water prior to entering photosynthesizing cells, at which point this aqueous CO_2_ interconverts with other DIC species. This raises the possibility of a similar strategy—pumping ${\mathrm{HCO}}_{3}^{-}$ from a land plant's apoplast water into the intracellular environment to increase net CO_2_ fixation—potentially viable. However, our results indicate that the limited spontaneous rate of CO_2_ and ${\mathrm{HCO}}_{3}^{-}$ interconversion without the activity of CA means that this strategy does not work.

Of note here is the fact that a quantitatively very similar system arises in algae growing in acidic environments where external ${\mathrm{HCO}}_{3}^{-}$ levels are negligible, such as the red alga *Cyanidioschyzon merolae* (De Luca et al. 1978). In such systems, all DIC must first enter the cell passively as aqueous CO_2_, at which point it will interconvert primarily between CO_2_ and HCO_3_, with the ratio of CO_2_:HCO_3_ determined by the cytosolic pH. There is strong evidence that *C. merolae* has a non-pyrenoid-based CCM (Steensma et al. 2023). Such a system could use ${\mathrm{HCO}}_{3}^{-}$ pumping across the chloroplast envelope as a method of concentrating carbon, but our results suggest that this system would require maintenance of a near-neutral cytosolic pH along with the presence of CAs in the cytosol to be viable. The maintenance of this near-neutral pH in an acidic environment may, in turn, represent a substantial energetic cost to the organism.

Due to how computationally intensive the simulations performed in this study were, as well as how many parameter combinations were explored, the spatial resolution at which the simulations were performed was not as granular as in Fei et al. (2022). Consequently, although relative changes to predicted rubisco saturation and quantum yield were extremely small with changes in spatial resolution, the sensitivity of model predictions to these spatial resolution changes was still greater than that reported in (Fei et al. 2022), Additionally, a number of simplifications were made to the geometry of the land plant and algal models. However, the use of the Virtual Cell software (Schaff et al. 1997; Cowan et al. 2012) makes it easy for other researchers to re-use the models presented in this study. Given the good performance of the presented models when validated against empirical compensation points and quantum yield measurements, we believe that these models represent a valuable contribution to efforts to model and understand the dynamics of carbon concentrating mechanisms, both actual and hypothetical.

## Materials and methods

### Model details

Spatially resolved reaction-diffusion models of carbon assimilation were developed in the *Virtual Cell* platform, a software suite that allows for the creation and analysis of chemical reaction-diffusion dynamics in the context of 3D models (Schaff et al. 1997; Cowan et al. 2012). Baseline parameters for simulations can be found in Table 1 and diagrams of 2 of the models used in this study, showing the representative features of the land plant and algal models, as well as the differences between the with- and without-PCCM models, can be seen in Fig. 1 (additional models are shown in Supplementary Fig. S1). The models used to describe hornworts were identical to those used to describe general land plants, except with (i) thicker and less permeable cell walls and (ii) substomatal airspaces removed to represent the undifferentiated thallus architecture of a hornwort gametophyte (Duckett and Pressel 2018).

Systems were represented as spatially symmetrical, with spherical concentric compartments that were converted into volumetric pixels (voxels) according to the simulations' spatial resolution. All results presented are from simulations containing either 9,261 or 12,167 voxels. Due to the large parameter explorations done in this study, minor geometrical modifications were made to make efficient numerical simulation feasible. Specifically, the radius of the apoplast water layer in the land plant models was extended out from 9.41 *μ*m and it should be based on a cell wall thickness of 0.32 *μ*m plus an apoplast water layer of equivalent thickness to 10 *μ*m. We also modeled the thylakoid tubules of with-PCCM models as a set of 6 cylinders of radius 0.5 *μ*m extending into the pyrenoid, with exchange between the tubules and the pyrenoid occurring at the end of these cylinders, in contrast to the larger number of finer tubules used in Fei et al. (2022).

### Reaction equations

Carboxylation flux by rubisco is calculated as in Farquhar et al. (1980) (E1). The rate of carboxylation by rubisco is normally taken to be the minimum of *V*_c_ and *J*, where *J* describes the rate of RuBP regeneration enabled by photosynthetic electron transport and a function of *J*_max_, a maximum rate of RuBP regeneration, among other parameters (Farquhar et al. 1980). Estimates of the relevant parameters are available for land plants but, to our knowledge, not for algae. We are also specifically examining CO_2_-limiting conditions where rubisco reaction rate limitations dominate. For these reasons, we calculate the carboxylation and oxygenation rates assuming that the system is not limited by RuBP regeneration as in Fei et al. (2022).


(E1) $$\begin{array}{c} V_{c}=\frac{\left(V_{\max}^{\mathrm{carboxylation}}*[CO_{2}]\right)}{\left([CO_{2}]+K_{m}^{CO_{2}}\left(1+\frac{\left[O_{2}\right]}{K_{m}^{O_{2}}}\right)\right)}\; \end{array}$$

The ratio of oxygenation to carboxylation *V*_max_ is:


(E2) $$\begin{array}{c} \frac{V_{\max}^{\mathrm{oxygenation}}}{V_{\max}^{\mathrm{carboxylation}}}=\frac{k_{\mathrm{cat}}^{\mathrm{oxygenation}}}{k_{\mathrm{cat}}^{\mathrm{carboxylation}}}\; \end{array}$$

Using a $\frac{k_{\mathrm{cat}}^{\mathrm{oxygenation}}}{k_{\mathrm{cat}}^{\mathrm{carboxylation}}}$ value of 0.21 as in (Farquhar et al. 1980), we can thereby calculate the $V_{\max}^{\mathrm{oxygenation}}$ of our systems. The oxygenation flux by rubisco is then calculated as follows:


(E3) $$\begin{array}{c} V_{o}=\frac{\left(V_{\max}^{\mathrm{oxygenation}}*[O_{2}]\right)}{\left([O_{2}]+K_{m}^{O_{2}}\left(1+\frac{\left[CO_{2}\right]}{K_{m}^{CO_{2}}}\right)\right)}\; \end{array}$$

Interconversion of CO_2_ with bicarbonate via CA is described as in McGrath and Long (2014):


(E4) $$\begin{array}{c} \frac{[\mathrm{CA}]*CA_{\mathrm{kcat}}*\left([CO_{2}]-\frac{\left[{\mathrm{HCO}}_{3}^{-}][H^{+}\right]}{K_{\mathrm{eq}}}\right)}{K_{m}^{CO_{2}}+[{\mathrm{HCO}}_{3}^{-}]\left(\frac{K_{m}^{CO_{2}}}{K_{m}^{\mathrm{HC}O_{3}}}\right)+[CO_{2}]}\; \end{array}$$

In the land plant models, the flux density of dissolution of gaseous CO_2_ or O_2_ into the water layer is as in Hemond and Fechner (2022):


(E5) $$\begin{array}{c} \mathrm{FluxDensit}y_{\mathrm{WaterLayer}}=\;-\frac{D_{w}\left(C_{w}-\frac{C_{a}}{H}\right)}{\begin{array}{c} \delta_{w} \end{array}}\; \end{array}$$

where *D*_w_ is the diffusion rate of the dissolving species in water *C*_w_ and *C*_a_ are the concentrations of that species in the air and in the water layer, *H* is the dimensionless Henry's law constant, and $\delta_{w}$ is the length of the unstirred water layer into which the gas is dissolving. In our models, we assume the presence of a thin layer of water on top of the plant's cell wall that is the same thickness as the cell wall itself into which CO_2_ is dissolving.

Permeation of aqueous species through the cell wall is given by the following equation, as in McGrath and Long (2014):


(E6) $$\begin{array}{c} \mathrm{FluxDensit}y_{\mathrm{CellWall}}=\frac{D_{w}}{\delta_{\mathrm{CellWall}}}*\mathrm{EffectivePorosity}\; \end{array}$$

where EffectivePorosity is the porosity of the cell wall divided by the tortuosity of the cell wall.

For computational tractability, we combine the processes of gases dissolving into water and the aqueous species passing through the cell wall. Note that in the above equation, *D*_w_/$\delta_{w}$ and *D*_w_* EffectivePorosity/$\delta_{\mathrm{CellWall}}$ gives permeability (in units of um/s) of the water layer and the cell wall, respectively. Multiplying these values by surface area gives conductivities (in units of µm^3^/s). The inverses of these values are resistances, which can be summed to give the total resistance of the water layer plus the cell wall. The inverse of this, again, will be the conductivity of the overall system, which can be multiplied by the concentration gradient from the air to the surface of the plasmalemma to give the total flux.


(E7) $$\begin{array}{rl} & J={\left({\left(\frac{D_{w}*\mathrm{SurfaceArea}}{\delta_{w}}\right)}^{-1}+{\left(\frac{D_{w}*\mathrm{EffectivePorosity}*\mathrm{SurfaceArea}}{\delta_{\mathrm{CellWall}}}\right)}^{-1}\right)}^{-1}\\ & \;*\left(\frac{C_{a}}{H}-C_{w}\right) \end{array}$$

Permeation through lipid membranes and passive transport through channels is described by:


(E8) $$\begin{array}{c} P*(\left[\mathrm{Outside}]-[\mathrm{Inside}\right])\; \end{array}$$

Diffusion of a chemical species C through a compartment is given by Fick's 2nd Law of Diffusion:


(E9) $$\begin{array}{c} \frac{\partial [C]}{\partial t}=D\nabla^{2}[C] \end{array}$$

where [*C*] is the concentration of the chemical species and *D* is the diffusion rate.

Active transport by bicarbonate transporter *BicA* is described using Michaelis–Menten kinetics:


(E10) $$\begin{array}{c} \frac{V_{\max\mathrm{BicA}}*[{\mathrm{HCO}}_{3}^{-}\;]}{K_{m}*[{\mathrm{HCO}}_{3}^{-}]}\; \end{array}$$

The release of CO_2_ due to photorespiratory processes are treated as occurring in the cytosol as the mitochondria are not modeled. The *Virtual Cell* does not support the direct production of a species in one cellular compartment being driven by a reaction in another compartment, and so “dummy” species chemical species whose concentrations were treated as well-mixed and that move from one compartment to another at an arbitrary, extremely high rate were used to approximate the desired behavior (A1) in Fig. 1. The coupling of carboxylation and O_2_ evolved indirectly in the stroma (A2 in Fig. 1) was calculated as in (Fridlyand 1997) and similarly handled with well-mixed concentration calculations and high transport rates.

### Numerical solving

The changes in concentration of chemical species were represented with systems of partial differential equations (PDEs), of the general form:


(E11) $$\begin{array}{c} \frac{\partial {\left[C\right]}_{\mathrm{comp}}(x)}{\partial t}=D_{\mathrm{comp}}^{\left[C\right]}\nabla^{2}{\left[C\right]}_{\mathrm{comp}}+J_{\mathrm{rx}}-J_{\mathrm{ry}} \end{array}$$

where [*C*]_comp_(*x*) is the concentration of a chemical species in a given compartment and at a spatial coordinate defined by the vector *x*, *D* is the diffusion coefficient of that species in a compartment, and *J*_rx_ and *J*_ry_ are intracompartmental fluxes.

Fluxes between compartments were represented by Neumann boundary conditions:


(E12) $$\begin{array}{c} \frac{\partial {\left[C\right]}_{\mathrm{comp}}(x)}{\partial t}=J_{\mathrm{rx}}\;\forall x\in \partial \Omega \; \end{array}$$

where *J*_rx_ is an intercompartmental flux and ∂Ω is the boundary.

Simulations were performed using the Virtual Cell platform's Fully Implicit Finite Volume solver, which discretizes and then integrates the PDEs above (Schaff et al. 1997). A full set of PDEs and boundary conditions can be found in the Supplementary Model Equations file.

### Efficiency calculations

Net CO_2_ fixation is described as follows:


(E13) $$\begin{array}{c} \mathrm{NetFixation}=\mathrm{Flu}x_{\mathrm{carboxylation}}-\frac{\mathrm{Flu}x_{\mathrm{oxygenation}}}{2}\; \end{array}$$

Two NADPH equivalents are expended per carboxylation or oxygenation reaction based on the stoichiometry of the CBC cycle and photorespiration. A total of 3 ATP and 3.5 ATP are used for a single carboxylation or oxygenation event, respectively (Edwards and Walker 1983).

In models featuring a PCCM, there is a luminal CA that catalyzes the following reaction:


(E14) $$\begin{array}{c} CO_{2}+H_{2}O\leftarrow \to {\mathrm{HCO}}_{3}^{-}+H^{+} \end{array}$$

Due to the acidic pH of the lumen (Kramer et al. 1999), the net flux of this reaction is overwhelmingly in the direction of CO_2_ and H_2_O so that entry of bicarbonate depletes the proton motive force (pmf) that is maintained by the light reactions of photosynthesis, which imposes an indirect ATP cost on CCM activity by requiring additional proton pumping to maintain the pmf (Mukherjee et al. 2019). Based on a 14:3 ratio of pumped protons to ATP synthesis via the thylakoid membrane ATP synthase, inferred from the number of c-subunits in such ATP synthases (Seelert et al. 2000), we can calculate the indirect ATP cost of this lumen CA activity as:


(E15) $$\begin{array}{c} \mathrm{AT}P_{\mathrm{cost}}=J_{\mathrm{CA}\mathrm{lumen}}*\frac{3}{14} \end{array}$$

This is added to the other sources of ATP consumption in the model (due to the metabolic costs of carboxylation and oxygenation) to give total ATP use. This can be compared with NADPH use due to carboxylation and oxygenation to get an estimate of the total ATP, NADPH, and the ATP:NADPH ratio needed to support the activity in the model. From the values provided by Walker et al. (2020), we estimate the amount of either CEF or malate valve activity needed to rebalance the ATP/NADPH ratio needed for a particular model, which we can then convert into an additional demand for photons and, therefore, a the number of photons needed on a per reaction (carboxylation or oxygenation) basis (Supplementary Fig. S7). From this, we can calculate the number of photons needed to support model fluxes and then compare this to the net fixation achieved by a model to get an estimate of light use efficiency.


(E16) $$\begin{array}{c} \varphi_{\mathrm{CEF}}=\frac{V_{c}-\frac{1}{2}V_{o}}{(V_{c}+V_{o})*\left(\mathrm{Photon}s_{\mathrm{base}}+((\mathrm{Ratio}*\mathrm{NADP}H_{\mathrm{base}})-\mathrm{AT}P_{\mathrm{base}})*0.43\frac{\mathrm{Photons}}{\mathrm{ATP}}\right)} \end{array}$$

(E17) $$\begin{array}{c} \varphi_{\mathrm{malate}}=\frac{V_{c}-\frac{1}{2}V_{o}}{(V_{c}+V_{o})*\left(\mathrm{Photon}s_{\mathrm{base}}+\frac{(\mathrm{Ratio}*\mathrm{NADP}H_{\mathrm{base}})-\mathrm{AT}P_{\mathrm{base}}}{\frac{5.45\mathrm{ATP}}{2\mathrm{NADPH}}}*4\frac{\mathrm{Photons}}{\mathrm{NADPH}}\right)} \end{array}$$

where *V*_c_ and *V*_o_ are the modeled rates of carboxylation and oxygenation, Ratio refers to the modeled ATP/NADPH ratio necessary to support the fluxes in the model, and Photons_base_, ATP_base_, and NADPH_base_ refer to the photons used and the ATP and NADPH generated in the process of making 2 NADPH molecules via linear electron flow (Walker et al. 2020).

We find that the depletion of protons from the stroma required to support the indirect ATP cost calculated in (E12), together with the generation of protons by the stromal CA, results in an extremely small flux of protons into the stroma being needed to maintain pH balance. These protons could be supplied by the cytosol via passive transport, due to its lower pH; in turn, the cytosol could use passive transport to get these protons from the extracellular environment, in cases where cells are in slightly acidic environments or active transport could be used in slightly basic environments. Assuming similar proton pumping stoichiometry as at the thylakoid membrane, ATP costs associated with such active transport would be on the order of 0.01% of the overall ATP costs associated with running the PCCM. Because of the uncertainty and contextual sensitivity of the energetic costs and biochemical details of how land plant and algal systems might achieve pH balancing, as well as the extremely small cost associated with this activity relative to other costs in the model, we have ignored these balancing fluxes in our energy cost calculations.

### Sensitivity analysis

Local sensitivity analysis was performed by varying input parameter values up or down by 10% and then calculating % change in rubisco saturation and quantum yield relative to those same values when using baseline parameter values. The average of the percent change values from increasing and decreasing parameter values is reported in Fig. 3 and Supplementary Fig. S3 and calculations can be found in Supplementary Tables S9 and S10. The effect of model geometry was explored by varying compartment radii and recalculating associated surface areas and volumes (e.g. when varying the radius of the pyrenoid, the surface area and volume of the pyrenoid were altered, but all other radii, surface areas, and volumes were kept constant). The density of membrane-bound and water-soluble proteins, and thus enzyme activity on a per µm^2^ or µm^3^ basis, was kept constant when varying radii.

### Concentration calculations

All concentrations in the models used in this study are in units of µM. To calculate the µM concentrations of CO_2_ and O_2_ in the atmosphere, we used the following conversions:


$$\frac{412\;\mu \mathrm{mol}\;CO_{2}}{\mathrm{mol}\;\mathrm{air}}*\frac{1\;\mathrm{mol}\;\mathrm{air}}{24.79\;L\;\mathrm{air}}=∼\frac{16.62\;\mu mol\;CO_{2}}{L\;\mathrm{air}}$$

$$\frac{0.2095\;\mathrm{mol}\;O_{2}}{\mathrm{mol}\;\mathrm{air}}*\frac{1\;\mathrm{mol}\;\mathrm{air}}{24.79\;L\;\mathrm{air}}=∼\frac{8450.98\;\mu mol\;O_{2}}{L\;\mathrm{air}}$$

## Supplementary Material

kiae324_Supplementary_Data

## Data Availability

All results generated as part of this study can be found in the Supplementary Material (Supplementary Tables S1 and S22). Supplementary Models S1 to S26 used for generating the results can all be found under the account *kastejos* in the Virtual Cell interface. Specific model names can be found for each dataset in the corresponding Supplementary Material tables. *Virtual Cell* model files (.vcml) can also be found in the Supplementary Material.

## Supplementary data

The following materials are available in the online version of this article.

**Supplementary Figure S1.** Diagrammatic representations of additional models presented in this study.

**Supplementary Figure S2.** Sensitivity analysis for models presented in this study at 50% external CO_2_ levels.

**Supplementary Figure S3.** Effect of spatial resolution on rubisco saturation in the model of an algal cell without a CCM.

**Supplementary Figure S4.** Effect of simulation spatial resolution on quantum yield in the model of an algal cell without a CCM.

**Supplementary Figure S5.** Effect of simulation spatial resolution on rubisco saturation in the model of an algal cell with a CCM.

**Supplementary Figure S6.** Effect of simulation spatial resolution on quantum yield in the model of an algal cell with a CCM.

**Supplementary Figure S7.** Carboxylation/oxygenation events per photon as a function of varying ATP:NADPH ratios.

**Supplementary Table S1.** C3 plant compensation point estimation, without PCCM.

**Supplementary Table S2.** C3 plant compensation point estimation, with PCCM.

**Supplementary Table S3.** Algae compensation point estimation, without PCCM.

**Supplementary Table S4.** Algae compensation point estimation, with PCCM.

**Supplementary Table S5.** C3 plant parameter variation results, with PCCM.

**Supplementary Table S6.** C3 plant parameter variation results, without PCCM.

**Supplementary Table S7.** Algae parameter variation results, with PCCM.

**Supplementary Table S8.** Algae parameter variation results, without PCCM.

**Supplementary Table S9.** Sensitivity estimation (100% CO_2_).

**Supplementary Table S10.** Sensitivity estimation (50% CO_2_).

**Supplementary Table S11.** C3 plant chloroplast ${\mathrm{HCO}}_{3}^{+}$ pumping activity vs. lipid membrane permeability to CO_2_.

**Supplementary Table S12.** Algal chloroplast ${\mathrm{HCO}}_{3}^{+}$ pumping activity v. lipid membrane permeability to CO_2_.

**Supplementary Table S13.** C3 plant plasmalemma ${\mathrm{HCO}}_{3}^{+}$ pumping activity vs. lipid membrane permeability to CO_2_.

**Supplementary Table S14.** Algal plasmalemma ${\mathrm{HCO}}_{3}^{+}$ pumping activity vs. lipid membrane permeability to CO_2_.

**Supplementary Table S15.** C3 plant plasmalemma ${\mathrm{HCO}}_{3}^{+}$ pumping activity vs. lipid membrane permeability to CO_2_ with CA activity.

**Supplementary Table S16.** C3 plant plasmalemma ${\mathrm{HCO}}_{3}^{+}$ pumping activity vs. lipid membrane permeability to CO_2_ with CA activity and apoplast pH of 8.

**Supplementary Table S17.** C3 plant external [CO_2_] vs. lipid membrane permeability to CO_2_, without PCCM.

**Supplementary Table S18.** C3 plant external [CO_2_] vs. lipid membrane permeability to CO_2_, with PCCM.

**Supplementary Table S19.** Algae external [CO_2_] vs. lipid membrane permeability to CO_2_, without PCCM.

**Supplementary Table S20.** Algae external [CO_2_] vs. lipid membrane permeability to CO_2_, with PCCM.

**Supplementary Table S21.** Hornwort external [CO_2_] vs. lipid membrane permeability to CO_2_, without PCCM.

**Supplementary Table S22.** Hornwort external [CO_2_] vs. lipid membrane permeability to CO_2_, with PCCM.

**Supplementary Model S1:** Virtual Cell model file (.vcml) for the land plant model without a PCCM.

**Supplementary Model S2:** Virtual Cell model file (.vcml) for the land plant model without a PCCM and with a 10% larger cytosol radius for sensitivity estimation.

**Supplementary Model S3:** Virtual Cell model file (.vcml) for the land plant model without a PCCM and with a 10% smaller cytosol radius for sensitivity estimation.

**Supplementary Model S4:** Virtual Cell model file (.vcml) for the land plant model without a PCCM and with a 10% larger chloroplast radius for sensitivity estimation.

**Supplementary Model S5:** Virtual Cell model file (.vcml) for the land plant model without a PCCM and with a 10% smaller chloroplast radius for sensitivity estimation.

**Supplementary Model S6:** Virtual Cell model file (.vcml) for the land plant model with a PCCM.

**Supplementary Model S7:** Virtual Cell model file (.vcml) for the land plant model with a PCCM and with a 10% larger cytosol radius for sensitivity estimation.

**Supplementary Model S8:** Virtual Cell model file (.vcml) for the land plant model with a PCCM and with a 10% smaller cytosol radius for sensitivity estimation.

**Supplementary Model S9:** Virtual Cell model file (.vcml) for the land plant model with a PCCM and with a 10% larger chloroplast radius for sensitivity estimation.

**Supplementary Model S10:** Virtual Cell model file (.vcml) for the land plant model with a PCCM and with a 10% smaller chloroplast radius for sensitivity estimation.

**Supplementary Model S11:** Virtual Cell model file (.vcml) for the land plant model with a PCCM and with a 10% larger pyrenoid radius for sensitivity estimation.

**Supplementary Model S12:** Virtual Cell model file (.vcml) for the land plant model with a PCCM and with a 10% smaller pyrenoid radius for sensitivity estimation.

**Supplementary Model S13:** Virtual Cell model file (.vcml) for the algal model without a PCCM.

**Supplementary Model S14:** Virtual Cell model file (.vcml) for the algal model without a PCCM and with a 10% larger cytosol radius for sensitivity estimation.

**Supplementary Model S15:** Virtual Cell model file (.vcml) for the algal model without a PCCM and with a 10% smaller cytosol radius for sensitivity estimation.

**Supplementary Model S16:** Virtual Cell model file (.vcml) for the algal model without a PCCM and with a 10% larger chloroplast radius for sensitivity estimation.

**Supplementary Model S17:** Virtual Cell model file (.vcml) for the algal model without a PCCM and with a 10% smaller chloroplast radius for sensitivity estimation.

**Supplementary Model S18:** Virtual Cell model file (.vcml) for the algal model with a PCCM.

**Supplementary Model S19:** Virtual Cell model file (.vcml) for the algal model with a PCCM and with a 10% larger cytosol radius for sensitivity estimation.

**Supplementary Model S20:** Virtual Cell model file (.vcml) for the algal model with a PCCM and with a 10% smaller cytosol radius for sensitivity estimation.

**Supplementary Model S21:** Virtual Cell model file (.vcml) for the algal model with a PCCM and with a 10% larger chloroplast radius for sensitivity estimation.

**Supplementary Model S22:** Virtual Cell model file (.vcml) for the algal model with a PCCM and with a 10% smaller cytosol radius for sensitivity estimation.

**Supplementary Model S23:** Virtual Cell model file (.vcml) for the algal model with a PCCM and with a 10% larger pyrenoid radius for sensitivity estimation.

**Supplementary Model S24:** Virtual Cell model file (.vcml) for the algal model with a PCCM and with a 10% smaller pyrenoid radius for sensitivity estimation.

**Supplementary Model S25:** Virtual Cell model file (.vcml) for the hornwort model without a PCCM.

**Supplementary Model S26:** Virtual Cell model file (.vcml) for the hornwort model with a PCCM.

**Supplementary Model Equations**: Full equation lists for models presented in this study.

## Dive Curated Terms

The following phenotypic, genotypic, and functional terms are of significance to the work described in this paper:

ATP CHEBI: CHEBI:15422

NADPH CHEBI: CHEBI:16474

carbon CHEBI: CHEBI:27594

quantum yield Planteome: TO:0012004
