# Supplementary material for: Reaction-diffusion modeling provides insights into biophysical carbon-concentrating mechanisms in land plants
Source: Plant Physiol. 2024 Jun 10;196(2):1374–90. doi: 10.1093/plphys/kiae324 (PMC11444298; doi:10.1093/plphys/kiae324)
Supplement: kiae324_Supplementary_Data [file kiae324_supplementary_data.zip › PP2024RA00046R2_Supplementary_Model_Equations.pdf]

Supplementary Model Equations

1 Land plant with PCCM Model

1.1 Parameter Derivations

$$Res^{O_2}_{Air} = (\frac{D_{O_2} * SA_{apoplast}}{Thickness_{apoplast}})^{-1} \tag{1}$$

$$Res^{O_2}_{CW} = (EffectivePorosity_{C3}(\frac{D_{O_2} * SA_{apoplast}}{Thickness_{apoplast}}))^{-1} \tag{2}$$

$$Res^{CO_2}_{Air} = (\frac{D_{CO_2} * SA_{apoplast}}{Thickness_{apoplast}})^{-1} \tag{3}$$

$$Res^{CO_2}_{CW} = (EffectivePorosity_{C3}(\frac{D_{CO_2} * SA_{apoplast}}{Thickness_{apoplast}}))^{-1} \tag{4}$$

$$SA_{pyrenoid} = 4\pi r^2_{pyrenoid} \tag{5}$$

$$SA_{thylakoid} = \pi r^2_{thylakoid} height_{thylakoid} \tag{6}$$

$$SA_{chloroplast} = 4\pi r^2_{chloroplast} \tag{7}$$

$$SA_{cytosol} = 4\pi r^2_{cytosol} \tag{8}$$

$$SA_{plasmalemmasurface} = 2\pi r^2_{thylakoid} + 2\pi r_{thylakoid} height_{thylakoid} \tag{9}$$

$$Vol_{pyrenoid} = \frac{4}{3}\pi r^3_{pyrenoid} \tag{10}$$

$$Vol_{thylakoid} = \pi r^2_{thylakoid} height_{thylakoid} \tag{11}$$

$$Vol_{chloroplast} = \frac{4}{3}\pi r^3_{chloroplast} \tag{12}$$

$$Vol_{cytosol} = \frac{4}{3}\pi r^3_{cytosol} \tag{13}$$

$$Vol_{plasmalemmasurface} = \frac{4}{3}\pi r^3_{plasmalemmasurface} \tag{14}$$

$$Vol_{substomatalspace} = \frac{4}{3}\pi r^3_{substomatalspace} \tag{15}$$

$$[H^+]_{cyt} = 10^{-pH_{cyt}} \tag{16}$$

$$[H^+]_{str} = 10^{-pH_{str}} \tag{17}$$

$$[H^+]_{lum} = 10^{-pH_{lum}} \tag{18}$$

$$D^{O_2}_{apo} = D_{O_2} \tag{19}$$

$$D^{O_2}_{cyt} = \frac{D^{O_2}}{Visc_{cytosol}} \tag{20}$$

$$D_{thy}^{O_2} = \frac{D^{O_2}}{Visc_{thylakoid}} \tag{21}$$

$$D_{str}^{O_2} = \frac{D^{O_2}}{Visc_{stroma}} \tag{22}$$

$$D_{pyr}^{O_2} = \frac{D^{O_2}}{Visc_{pyrenoid}} \tag{23}$$

$$D_{apo}^{CO_2} = D_{CO_2} \tag{24}$$

$$D_{cyt}^{CO_2} = \frac{D^{CO_2}}{Visc_{cytosol}} \tag{25}$$

$$D_{thy}^{CO_2} = \frac{D^{CO_2}}{Visc_{thylakoid}} \tag{26}$$

$$D_{str}^{CO_2} = \frac{D^{CO_2}}{Visc_{stroma}} \tag{27}$$

$$D_{pyr}^{CO_2} = \frac{D^{CO_2}}{Visc_{pyrenoid}} \tag{28}$$

$$D_{apo}^{H_2CO_3} = D_{H_2CO_3} \tag{29}$$

$$D_{cyt}^{H_2CO_3} = \frac{D^{H_2CO_3}}{Visc_{cytosol}} \tag{30}$$

$$D_{thy}^{H_2CO_3} = \frac{D^{H_2CO_3}}{Visc_{thylakoid}} \tag{31}$$

$$D_{str}^{H_2CO_3} = \frac{D^{H_2CO_3}}{Visc_{stroma}} \tag{32}$$

$$D_{pyr}^{H_2CO_3} = \frac{D^{H_2CO_3}}{Visc_{pyrenoid}} \tag{33}$$

$$D_{apo}^{HCO_3} = D_{HCO_3} \tag{34}$$

$$D_{cyt}^{HCO_3} = \frac{D^{HCO_3}}{Visc_{cytosol}} \tag{35}$$

$$D_{thy}^{HCO_3} = \frac{D^{HCO_3}}{Visc_{thylakoid}} \tag{36}$$

$$D_{str}^{HCO_3} = \frac{D^{HCO_3}}{Visc_{stroma}} \tag{37}$$

$$D_{pyr}^{HCO_3} = \frac{D^{HCO_3}}{Visc_{pyrenoid}} \tag{38}$$

1.2 Partial Differential Equations for Volume Concentrations

1.2.1 Apoplast

$$\frac{\partial[O_2]_{apo}(x)}{\partial t} = D_{apo}^{O_2}\nabla^2[O_2]_{apo} \tag{39}$$

$$\frac{\partial[CO_2]_{apo}(x)}{\partial t} = D_{apo}^{CO_2}\nabla^2[CO_2]_{apo} - J_{R4} \tag{40}$$

$$\frac{\partial[H_2CO_3]_{apo}(x)}{\partial t} = D_{apo}^{H_2CO_3}\nabla^2[H_2CO_3]_{apo} + J_{R4} - J_{R5} \tag{41}$$

$$\frac{\partial[HCO_3^+]_{apo}(x)}{\partial t} = D_{apo}^{HCO_3^+}\nabla^2[HCO_3^+]_{apo} - J_{R5} \tag{42}$$

1.2.2 Cytosol

$$\frac{\partial[O_2]_{cyt}(x)}{\partial t} = D_{cyt}^{O_2}\nabla^2[O_2]_{cyt} \tag{43}$$

$$\frac{\partial[CO_2]_{cyt}(x)}{\partial t} = D_{cyt}^{CO_2}\nabla^2[CO_2]_{cyt} - J_{R4} - J_{R8} + J_{R17} \tag{44}$$

$$\frac{\partial[H_2CO_3]_{cyt}(x)}{\partial t} = D_{cyt}^{H_2CO_3}\nabla^2[H_2CO_3]_{cyt} + J_{R6} - J_{R7} \tag{45}$$

$$\frac{\partial[HCO_3^+]_{cyt}(x)}{\partial t} = D_{cyt}^{HCO_3^+}\nabla^2[HCO_3^+]_{cyt} + J_{R7} + J_{R8} \tag{46}$$

1.2.3 Stroma

$$\frac{\partial[O_2]_{str}(x)}{\partial t} = D_{str}^{O_2}\nabla^2[O_2]_{str} \tag{47}$$

$$\frac{\partial[CO_2]_{str}(x)}{\partial t} = D_{str}^{CO_2}\nabla^2[CO_2]_{str} - J_{R9} - J_{R11} \tag{48}$$

$$\frac{\partial[H_2CO_3]_{str}(x)}{\partial t} = D_{str}^{H_2CO_3}\nabla^2[H_2CO_3]_{str} + J_{R9} - J_{R10} \tag{49}$$

$$\frac{\partial[HCO_3^+]str(x)}{\partial t} = D_{str}^{HCO_3^+}\nabla^2[HCO_3^+]_{str} + J_{R10} + J_{R11} \tag{50}$$

1.2.4 Lumen

$$\frac{\partial[O_2]_{lum}(x)}{\partial t} = D_{lum}^{O_2}\nabla^2[O_2]_{lum} \tag{51}$$

$$\frac{\partial[CO_2]_{lum}(x)}{\partial t} = D_{lum}^{CO_2}\nabla^2[CO_2]_{lum} - J_{R23} \tag{52}$$

$$\frac{\partial[H_2CO_3]_{lum}(x)}{\partial t} = D_{lum}^{H_2CO_3}\nabla^2[H_2CO_3]_{lum} + J_{R23} - J_{R24} \tag{53}$$

$$\frac{\partial[HCO_3^+]_{lum}(x)}{\partial t} = D_{lum}^{HCO_3^+}\nabla^2[HCO_3^+]_{lum} + J_{R24} \tag{54}$$

1.2.5 Pyrenoid

$$\frac{\partial[O_2]_{pyr}(x)}{\partial t} = D_{pyr}^{O_2}\nabla^2[O_2]_{pyr} - J_{R16} \tag{55}$$

$$\frac{\partial[CO_2]_{pyr}(x)}{\partial t} = D_{pyr}^{CO_2}\nabla^2[CO_2]_{pyr} - J_{R12} - J_{R14} - J_{R18} \tag{56}$$

$$\frac{\partial[H_2CO_3]_{pyr}(x)}{\partial t} = D_{pyr}^{H_2CO_3}\nabla^2[H_2CO_3]_{pyr} + J_{R12} - J_{R13} \tag{57}$$

$$\frac{\partial[HCO_3^+]_{pyr}(x)}{\partial t} = D_{pyr}^{HCO_3^+}\nabla^2[HCO_3^+]_{pyr} + J_{R24} + J_{R14} \tag{58}$$

1.3 Boundary Conditions

1.3.1 Pyrenoid to Lumen

$$\frac{\partial[O_2]_{pyr}(x)}{\partial t} = J_{F14}\forall x \in \delta\Omega \tag{59}$$

$$\frac{\partial[CO_2]_{pyr}(x)}{\partial t} = J_{F15}\forall x \in \delta\Omega \tag{60}$$

$$\frac{\partial[H_2CO_3]_{pyr}(x)}{\partial t} = J_{F16}\forall x \in \delta\Omega \tag{61}$$

$$\frac{\partial[HCO_3^+]_{pyr}(x)}{\partial t} = J_{F17} + J_{F27}\forall x \in \delta\Omega \tag{62}$$

$$-\frac{\partial[O_2]_{lum}(x)}{\partial t} = J_{F14}\forall x \in \delta\Omega \tag{63}$$

$$-\frac{\partial[CO_2]_{lum}(x)}{\partial t} = J_{F15}\forall x \in \delta\Omega \tag{64}$$

$$-\frac{\partial[H_2CO_3]_{lum}(x)}{\partial t} = J_{F16}\forall x \in \delta\Omega \tag{65}$$

$$-\frac{\partial[HCO_3^+]_{lum}(x)}{\partial t} = J_{F17} + J_{F27}\forall x \in \delta\Omega \tag{66}$$

1.3.2 Pyrenoid to Stroma

$$\frac{\partial [O_2]_{pyr}(x)}{\partial t} = J_{F0} \forall x \in \delta\Omega \tag{67}$$

$$\frac{\partial [CO_2]_{pyr}(x)}{\partial t} = J_{F1} \forall x \in \delta\Omega \tag{68}$$

$$\frac{\partial [H_2CO_3]_{pyr}(x)}{\partial t} = J_{F2} \forall x \in \delta\Omega \tag{69}$$

$$\frac{\partial [HCO_3^+]_{pyr}(x)}{\partial t} = J_{F3} \forall x \in \delta\Omega \tag{70}$$

$$-\frac{\partial [O_2]_{lum}(x)}{\partial t} = J_{F0} + J_{F12} \forall x \in \delta\Omega \tag{71}$$

$$-\frac{\partial [CO_2]_{lum}(x)}{\partial t} = J_{F1} \forall x \in \delta\Omega \tag{72}$$

$$-\frac{\partial [H_2CO_3]_{lum}(x)}{\partial t} = J_{F2} \forall x \in \delta\Omega \tag{73}$$

$$-\frac{\partial [HCO_3^+]_{lum}(x)}{\partial t} = J_{F3} \forall x \in \delta\Omega \tag{74}$$

1.3.3 Lumen to Stroma

$$\frac{\partial [O_2]_{lum}(x)}{\partial t} = J_{F18} \forall x \in \delta\Omega \tag{75}$$

$$\frac{\partial [CO_2]_{lum}(x)}{\partial t} = J_{F19} \forall x \in \delta\Omega \tag{76}$$

$$\frac{\partial [H_2CO_3]_{lum}(x)}{\partial t} = J_{F20} \forall x \in \delta\Omega \tag{77}$$

$$\frac{\partial [HCO_3^+]_{lum}(x)}{\partial t} = J_{F21} + J_{F26} \forall x \in \delta\Omega \tag{78}$$

$$-\frac{\partial [O_2]_{str}(x)}{\partial t} = J_{F18} \forall x \in \delta\Omega \tag{79}$$

$$-\frac{\partial [CO_2]_{str}(x)}{\partial t} = J_{F19} \forall x \in \delta\Omega \tag{80}$$

$$-\frac{\partial [H_2CO_3]_{str}(x)}{\partial t} = J_{F20} \forall x \in \delta\Omega \tag{81}$$

$$-\frac{\partial [HCO_3^+]_{str}(x)}{\partial t} = J_{F21} + J_{F26} \forall x \in \delta\Omega \tag{82}$$

1.3.4 Stroma to Cytosol

$$\frac{\partial[O_2]_{str}(x)}{\partial t} = J_{F4} \forall x \in \delta\Omega \tag{83}$$

$$\frac{\partial[CO_2]_{str}(x)}{\partial t} = J_{F5} \forall x \in \delta\Omega \tag{84}$$

$$\frac{\partial[H_2CO_3]_{str}(x)}{\partial t} = J_{F6} \forall x \in \delta\Omega \tag{85}$$

$$\frac{\partial[HCO_3^+]_{str}(x)}{\partial t} = J_{F7} + J_{F22} + J_{F1} \forall x \in \delta\Omega \tag{86}$$

$$-\frac{\partial[O_2]_{cyt}(x)}{\partial t} = J_{F4} \forall x \in \delta\Omega \tag{87}$$

$$-\frac{\partial[CO_2]_{cyt}(x)}{\partial t} = J_{F5} \forall x \in \delta\Omega \tag{88}$$

$$-\frac{\partial[H_2CO_3]_{cyt}(x)}{\partial t} = J_{F6} \forall x \in \delta\Omega \tag{89}$$

$$-\frac{\partial[HCO_3^+]_{cyt}(x)}{\partial t} = J_{F7} + J_{F22} + J_{F1} \forall x \in \delta\Omega \tag{90}$$

1.3.5 Cytosol to Apoplast

$$\frac{\partial[O_2]_{cyt}(x)}{\partial t} = J_{F8} \forall x \in \delta\Omega \tag{91}$$

$$\frac{\partial[CO_2]_{cyt}(x)}{\partial t} = J_{F9} \forall x \in \delta\Omega \tag{92}$$

$$\frac{\partial[H_2CO_3]_{cyt}(x)}{\partial t} = J_{F10} \forall x \in \delta\Omega \tag{93}$$

$$\frac{\partial[HCO_3^+]_{cyt}(x)}{\partial t} = J_{F11} + J_{r0} \forall x \in \delta\Omega \tag{94}$$

$$-\frac{\partial[O_2]_{apo}(x)}{\partial t} = J_{F8} \forall x \in \delta\Omega \tag{95}$$

$$-\frac{\partial[CO_2]_{apo}(x)}{\partial t} = J_{F9} \forall x \in \delta\Omega \tag{96}$$

$$-\frac{\partial[H_2CO_3]_{apo}(x)}{\partial t} = J_{F10} \forall x \in \delta\Omega \tag{97}$$

$$-\frac{\partial[HCO_3^+]_{apo}(x)}{\partial t} = J_{F11} + J_{r0} \forall x \in \delta\Omega \tag{98}$$

1.3.6 Apoplast to Substomatal Space

$$\frac{\partial [O_2]_{apo}(x)}{\partial t} = J_{R19} \forall x \in \delta\Omega \tag{99}$$

$$\frac{\partial [CO_2]_{apo}(x)}{\partial t} = J_{R20} \forall x \in \delta\Omega \tag{100}$$

$$\frac{\partial [H_2CO_3]_{apo}(x)}{\partial t} = 0 \tag{101}$$

$$\frac{\partial [HCO_3^+]_{apo}(x)}{\partial t} = 0 \tag{102}$$

$$-\frac{\partial [O_2]_{cyt}(x)}{\partial t} = J_{R19} \forall x \in \delta\Omega \tag{103}$$

$$-\frac{\partial [CO_2]_{cyt}(x)}{\partial t} = J_{R20} \forall x \in \delta\Omega \tag{104}$$

1.3.7 Substomatal Space to Exterior

$$\frac{\partial [O_2]_{apo}(x)}{\partial t} = J_{R2} \forall x \in \delta\Omega \tag{105}$$

$$\frac{\partial [CO_2]_{apo}(x)}{\partial t} = J_{R3} \forall x \in \delta\Omega \tag{106}$$

1.4 Fluxes

$$J_{F0} = P_{O_2}^{str/pyr} * ([O_2]_{str} - [O_2]_{pyr}) \tag{107}$$

$$J_{F1} = P_{CO_2}^{str/pyr} * ([CO_2]_{str} - [O_2]_{pyr}) \tag{108}$$

$$J_{F10} = P_{H_2CO_3}^{apo/cyt} * ([H_2CO_3]_{apo} - [H_2CO_3]_{cyt}) \tag{109}$$

$$J_{F11} = P_{HCO_3^+}^{apo/cyt} * ([HCO_3^+]_{apo} - [HCO_3^+]_{cyt}) \tag{110}$$

$$J_{F12} = P_{O_2}^{intermediate} * [O_2]_{pyr}^{intermediate} \tag{111}$$

$$J_{F13} = P_{PhotorespiratoryIntermediate} * [PhotorespiratoryIntermediate]_{pyr} \tag{112}$$

$$J_{F14} = P_{O_2}^{pyr/thy} * ([O_2]_{lum} * [O_2]_{pyr}) \tag{113}$$

$$J_{F15} = P_{CO_2}^{pyr/thy} * ([CO_2]_{lum} * [CO_2]_{pyr}) \tag{114}$$

$$J_{F16} = P_{H_2CO_3}^{pyr/thy} * ([H_2CO_3]_{lum} * [H_2CO_3]_{pyr}) \tag{115}$$

$$J_{F17} = P_{HCO_3^+}^{pyr/thy} * ([HCO_3^+]_{lum} * [HCO_3^+]_{pyr}) \tag{116}$$

$$J_{F18} = P_{O_2}^{str/thy} * ([O_2]_{str} * [O_2]_{lum}) \tag{117}$$

$$J_{F19} = P_{CO_2}^{str/thy} * ([CO_2]_{str} * [CO_2]_{lum}) \tag{118}$$

$$J_{F2} = P_{H_2CO_3}^{pyr/str} * ([H_2CO_3]_{str} * [H_2CO_3]_{pyr}) \tag{119}$$

$$J_{F20} = P_{H_2CO_3}^{str/thy} * ([H_2CO_3]_{str} * [H_2CO_3]_{lum}) \tag{120}$$

$$J_{F21} = P_{HCO_3^+}^{str/thy} * ([HCO_3^+]_{str} * [HCO_3^+]_{lum}) \tag{121}$$

$$J_{F22} = P_{HCO_3^+}^{cyt/str} * ([HCO_3^+]_{cyt} * [HCO_3^+]_{str}) \tag{122}$$

$$J_{F23} = P_{PhotorespiratoryIntermediate} * [PhotorespiratoryIntermediate]_{str} \tag{123}$$

$$J_{F26} = Pfacilitated_{HCO_3^+}^{str/thy} * ([HCO_3^+]_{str} * [HCO_3^+]_{lum}) \tag{124}$$

$$J_{F27} = P_{HCO_3^+}^{pyr/thy} * ([HCO_3^+]_{lum} * [HCO_3^+]_{pyr}) \tag{125}$$

$$J_{F3} = P_{HCO_3^+}^{pyr/str} * ([HCO_3^+]_{str} * [HCO_3^+]_{pyr}) \tag{126}$$

$$J_{F4} = P_{O_2}^{cyt/str} * ([O_2]_{cyt} * [O_3]_{str}) \tag{127}$$

$$J_{F5} = P_{CO_2}^{cyt/str} * ([CO_2]_{cyt} * [CO_2]_{str}) \tag{128}$$

$$J_{F6} = P_{H_2CO_3}^{cyt/str} * ([H_2CO_3]_{cyt} * [H_2CO_3]_{str}) \tag{129}$$

$$J_{F7} = P_{HCO_3^+}^{cyt/str} * ([HCO_3^+]_{cyt} * [HCO_3^+]_{str}) \tag{130}$$

$$J_{F8} = P_{O_2}^{apo/cyt} * ([O_2]_{apo} * [O_2]_{cyt}) \tag{131}$$

$$J_{F9} = P_{CO_2}^{apo/cyt} * ([CO_2]_{apo} * [CO_2]_{cyt} \tag{132}$$

$$J_{R1} = \frac{[HCO_3^+]_{cyt} * V_{max}^{BicA}}{[HCO_3^+]_{cyt} + Km^{BicA}} \tag{133}$$

$$J_{R10} = K_f^{deprotonation}[H_2CO_3]_{str} - K_r^{protonation}[HCO_3^+]_{str}[H^+]_{str} \tag{134}$$

$$J_{R11} = \frac{CA^{kcat}[CA]_{str}([CO_2]_{str} - \frac{([HCO_3^+]_{str}[H^+]_{str})}{Keq_{str}}}{CA^{km} + \frac{[HCO_3^+]_{str}CA_{CO_2}^{Km}}{CA_{HCO_3^+}^{Km}} + [CO_2]_{str}} \tag{135}$$

$$J_{R12} = K_f^{hydration}[CO_2]_{pyr} - K_r^{dehydration}[H_2CO_3]_{pyr} \tag{136}$$

$$J_{R13} = K_f^{deprotonation}[H_2CO_3]_{pyr} - K_r^{protonation}[HCO_3^+]_{pyr}[H^+]_{pyr} \tag{137}$$

$$J_{R14} = \frac{CA^{kcat}[CA]_{pyr}([CO_2]_{pyr} - \frac{([HCO_3^+]_{pyr}[H^+]_{lum})}{Keq_{lum}}}{CA^{km} + \frac{[HCO_3^+]_{pyr}CA_{CO_2}^{Km}}{CA_{HCO_3^+}^{Km}} + [CO_2]_{pyr}} \tag{138}$$

$$J_{R16} = \frac{Rubisco^{Vo}[O_2]_{pyr}}{[O_2]_{pyr} + (Rubisco^{Km}_{O_2}(1 + \frac{[CO_2]_{pyr}}{Rubisco^{Km}_{CO_2}}} \tag{139}$$

$$J_{R17} = P_{PhotorespiratoryIntermediate} * [PhotorespiratoryIntermediate]_{cyt} \tag{140}$$

$$J_{R18} = \frac{Rubisco^{Vc}[CO_2]_{pyr}}{[CO_2]_{pyr} + (Rubisco^{Km}_{CO_2}(1 + \frac{[O_2]_{pyr}}{Rubisco^{Km}_{O_2}}} \tag{141}$$

$$J_{R19} = \frac{6.022e23 * IAS * ([O_2]_{apo} - ([O_2]_{sub}\frac{HenryConstant^{O_2}}{1e21})}{SA_{apo}(Res_{air} + Res_{CW})} \tag{142}$$

$$J_{R2} = \frac{StomatalConductance_{multiplier}StomatalConductance([O_2]_{ext} - [O_2]_{sub}}{Molarity_{air}} \tag{143}$$

$$J_{R20} = \frac{6.022e23 * IAS * ([CO_2]_{apo} - ([CO_2]_{sub}\frac{HenryConstant^{CO_2}}{1e21})}{SA_{apo}(Res_{air} + Res_{CW})} \tag{144}$$

$$J_{R21} = 0.5J_{R16} \tag{145}$$

$$J_{R23} = K^{hydration}_{f}[CO_2]_{lum} - K^{dehydration}_{r}[H_2CO_3]_{lum} \tag{146}$$

$$J_{R24} = K^{deprotonation}_{f}[H_2CO_3]_{lum} - K^{protonation}_{r}[HCO_3^+]_{lum}[H^+]_{lum} \tag{147}$$

$$J_{R3} = \frac{StomatalConductance_{multiplier}StomatalConductance([CO_2]_{ext} - [CO_2]_{sub}}{Molarity_{air}} \tag{148}$$

$$J_{R4} = K^{hydration}_{f}[CO_2]_{apo} - K^{dehydration}_{r}[H_2CO_3]_{apo} \tag{149}$$

$$J_{R5} = K^{deprotonation}_{f}[H_2CO_3]_{apo} - K^{protonation}_{r}[HCO_3^+]_{apo}[H^+]_{apo} \tag{150}$$

$$J_{R6} = K^{hydration}_{f}[CO_2]_{cyt} - K^{dehydration}_{r}[H_2CO_3]_{cyt} \tag{151}$$

$$J_{R7} = K^{deprotonation}_{f}[H_2CO_3]_{cyt} - K^{protonation}_{r}[HCO_3^+]_{cyt}[H^+]_{cyt} \tag{152}$$

$$J_{R8} = \frac{CA^{kcat}[CA]_{cyt}([CO_2]_{cyt} - \frac{([HCO_3^+]_{cyt}[H^+]_{cyt})}{Keq_{cyt}}}{CA^{km} + \frac{[HCO_3^+]_{cyt}CA^{Km}_{CO_2}}{CA^{Km}_{HCO_3^+}} + [CO_2]_{cyt}} \tag{153}$$

$$J_{R9} = K^{hydration}_{f}[CO_2]_{str} - K^{dehydration}_{r}[H_2CO_3]_{str} \tag{154}$$

1.5 Aggregated Processes in Figure 1

$$A_1 = [R17, R21, F23, F13] \tag{155}$$

$$A_2 = [F12, R18] \tag{156}$$

2 Land plant without PCCM Model

2.1 Parameter Derivations

$$Res_{Air}^{O_2} = (\frac{D_{O_2} * SA_{apoplast}}{Thickness_{apoplast}})^{-1} \quad (157)$$

$$Res_{CW}^{O_2} = (EffectivePorosity_{C3}(\frac{D_{O_2} * SA_{apoplast}}{Thickness_{apoplast}}))^{-1} \quad (158)$$

$$Res_{Air}^{CO_2} = (\frac{D_{CO_2} * SA_{apoplast}}{Thickness_{apoplast}})^{-1} \quad (159)$$

$$Res_{CW}^{CO_2} = (EffectivePorosity_{C3}(\frac{D_{CO_2} * SA_{apoplast}}{Thickness_{apoplast}}))^{-1} \quad (160)$$

$$SA_{chloroplast} = 4\pi r_{chloroplast}^2 \quad (161)$$

$$SA_{cytosol} = 4\pi r_{cytosol}^2 \quad (162)$$

$$SA_{plasmalemmasurface} = 2\pi r_{thylakoid}^2 + 2\pi r_{thylakoid} height_{thylakoid} \quad (163)$$

$$Vol_{chloroplast} = \frac{4}{3}\pi r_{chloroplast}^3 \quad (164)$$

$$Vol_{cytosol} = \frac{4}{3}\pi r_{cytosol}^3 \quad (165)$$

$$Vol_{plasmalemmasurface} = \frac{4}{3}\pi r_{plasmalemmasurface}^3 \quad (166)$$

$$Vol_{substomatalspace} = \frac{4}{3}\pi r_{substomatalspace}^3 \quad (167)$$

$$[H^+]_{cyt} = 10^{-pH_{cyt}} \quad (168)$$

$$[H^+]_{str} = 10^{-pH_{str}} \quad (169)$$

$$D_{apo}^{O_2} = D_{O_2} \quad (170)$$

$$D_{cyt}^{O_2} = \frac{D^{O_2}}{Visc_{cytosol}} \quad (171)$$

$$D_{str}^{O_2} = \frac{D^{O_2}}{Visc_{stroma}} \quad (172)$$

$$D_{apo}^{CO_2} = D_{CO_2} \quad (173)$$

$$D_{cyt}^{CO_2} = \frac{D^{CO_2}}{Visc_{cytosol}} \quad (174)$$

$$D_{str}^{CO_2} = \frac{D^{CO_2}}{Visc_{stroma}} \quad (175)$$

$$D_{apo}^{H_2CO_3} = D_{H_2CO_3} \tag{176}$$

$$D_{cyt}^{H_2CO_3} = \frac{D^{H_2CO_3}}{Visc_{cytosol}} \tag{177}$$

$$D_{str}^{H_2CO_3} = \frac{D^{H_2CO_3}}{Visc_{stroma}} \tag{178}$$

$$D_{apo}^{HCO_3} = D_{HCO_3} \tag{179}$$

$$D_{cyt}^{HCO_3} = \frac{D^{HCO_3}}{Visc_{cytosol}} \tag{180}$$

$$D_{str}^{HCO_3} = \frac{D^{HCO_3}}{Visc_{stroma}} \tag{181}$$

2.2 Partial Differential Equations for Volume Concentrations

2.2.1 Apoplast

$$\frac{\partial[O_2]_{apo}(x)}{\partial t} = D_{apo}^{O_2}\nabla^2[O_2]_{apo} \tag{182}$$

$$\frac{\partial[CO_2]_{apo}(x)}{\partial t} = D_{apo}^{CO_2}\nabla^2[CO_2]_{apo} - J_{r2} \tag{183}$$

$$\frac{\partial[H_2CO_3]_{apo}(x)}{\partial t} = D_{apo}^{H_2CO_3}\nabla^2[H_2CO_3]_{apo} + J_{r2} - J_{r3} \tag{184}$$

$$\frac{\partial[HCO_3^+]_{apo}(x)}{\partial t} = D_{apo}^{HCO_3^+}\nabla^2[HCO_3^+]_{apo} + J_{r3} \tag{185}$$

2.2.2 Cytosol

$$\frac{\partial[O_2]_{cyt}(x)}{\partial t} = D_{cyt}^{O_2}\nabla^2[O_2]_{cyt} \tag{186}$$

$$\frac{\partial[CO_2]_{cyt}(x)}{\partial t} = D_{cyt}^{CO_2}\nabla^2[CO_2]_{cyt} - J_{r4} - J_{r9} + J_{r12} \tag{187}$$

$$\frac{\partial[H_2CO_3]_{cyt}(x)}{\partial t} = D_{cyt}^{H_2CO_3}\nabla^2[H_2CO_3]_{cyt} + J_{r4} - J_{r5} \tag{188}$$

$$\frac{\partial[HCO_3^+]_{cyt}(x)}{\partial t} = D_{cyt}^{HCO_3^+}\nabla^2[HCO_3^+]_{cyt} + J_{r7} + J_{r9} \tag{189}$$

2.2.3 Stroma

$$\frac{\partial[O_2]_{str}(x)}{\partial t} = D_{str}^{O_2}\nabla^2[O_2]_{str} - J_{r10} + J_{r13} \tag{190}$$

$$\frac{\partial[CO_2]_{str}(x)}{\partial t} = D_{str}^{CO_2}\nabla^2[CO_2]_{str} - J_{r6} - J_{r8} - J_{r11} \tag{191}$$

$$\frac{\partial[H_2CO_3]_{str}(x)}{\partial t} = D_{str}^{H_2CO_3}\nabla^2[H_2CO_3]_{str} + J_{r6} - J_{r7} \tag{192}$$

$$\frac{\partial[HCO_3^+]_{str}(x)}{\partial t} = D_{str}^{HCO_3^+}\nabla^2[HCO_3^+]_{str} + J_{r7} + J_{r8} \tag{193}$$

2.3 Boundary Conditions

2.3.1 Stroma to Cytosol

$$\frac{\partial[O_2]_{str}(x)}{\partial t} = J_{r5}\forall x \in \delta\Omega \tag{194}$$

$$\frac{\partial[CO_2]_{str}(x)}{\partial t} = J_{r6}\forall x \in \delta\Omega \tag{195}$$

$$\frac{\partial[H_2CO_3]_{str}(x)}{\partial t} = J_{r7}\forall x \in \delta\Omega \tag{196}$$

$$\frac{\partial[HCO_3^+]_{str}(x)}{\partial t} = J_{r8}\forall x \in \delta\Omega \tag{197}$$

$$-\frac{\partial[O_2]_{cyt}(x)}{\partial t} = J_{r5}\forall x \in \delta\Omega \tag{198}$$

$$-\frac{\partial[CO_2]_{cyt}(x)}{\partial t} = J_{r6}\forall x \in \delta\Omega \tag{199}$$

$$-\frac{\partial[H_2CO_3]_{cyt}(x)}{\partial t} = J_{r7}\forall x \in \delta\Omega \tag{200}$$

$$-\frac{\partial[HCO_3^+]_{cyt}(x)}{\partial t} = J_{r8}\forall x \in \delta\Omega \tag{201}$$

2.3.2 Cytosol to Apoplast

$$\frac{\partial[O_2]_{cyt}(x)}{\partial t} = J_{flux0}\forall x \in \delta\Omega \tag{202}$$

$$\frac{\partial[CO_2]_{cyt}(x)}{\partial t} = J_{flux1}\forall x \in \delta\Omega \tag{203}$$

$$\frac{\partial[H_2CO_3]_{cyt}(x)}{\partial t} = J_{flux2}\forall x \in \delta\Omega \tag{204}$$

$$\frac{\partial[HCO_3^+]_{cyt}(x)}{\partial t} = J_{flux3} + J_{r0} \forall x \in \delta\Omega \tag{205}$$

$$-\frac{\partial[O_2]_{apo}(x)}{\partial t} = J_{flux0} \forall x \in \delta\Omega \tag{206}$$

$$-\frac{\partial[CO_2]_{apo}(x)}{\partial t} = J_{flux1} \forall x \in \delta\Omega \tag{207}$$

$$-\frac{\partial[H_2CO_3]_{apo}(x)}{\partial t} = J_{flux2} \forall x \in \delta\Omega \tag{208}$$

$$-\frac{\partial[HCO_3^+]_{apo}(x)}{\partial t} = J_{flux3} + J_{r0} \forall x \in \delta\Omega \tag{209}$$

2.3.3 Apoplast to Substomatal Space

$$\frac{\partial[O_2]_{apo}(x)}{\partial t} = J_{r14} \forall x \in \delta\Omega \tag{210}$$

$$\frac{\partial[CO_2]_{apo}(x)}{\partial t} = J_{r15} \forall x \in \delta\Omega \tag{211}$$

$$\frac{\partial[H_2CO_3]_{apo}(x)}{\partial t} = 0 \tag{212}$$

$$\frac{\partial[HCO_3^+]_{apo}(x)}{\partial t} = 0 \tag{213}$$

$$-\frac{\partial[O_2]_{cyt}(x)}{\partial t} = J_{r14} \forall x \in \delta\Omega \tag{214}$$

$$-\frac{\partial[CO_2]_{cyt}(x)}{\partial t} = J_{r15} \forall x \in \delta\Omega \tag{215}$$

2.3.4 Substomatal Space to Exterior

$$\frac{\partial[O_2]_{apo}(x)}{\partial t} = J_{r18} \forall x \in \delta\Omega \tag{216}$$

$$\frac{\partial[CO_2]_{apo}(x)}{\partial t} = J_{r19} \forall x \in \delta\Omega \tag{217}$$

2.4 Fluxes

$$J_{F0} = P_{O_2}^{apo/cyt} * ([O_2]_{apo} - [O_2]_{cyt}) * SA_{multiplier} \tag{218}$$

$$J_{F1} = P_{CO_2}^{apo/cyt} * ([CO_2]_{apo} - [CO_2]_{cyt}) * SA_{multiplier} \tag{219}$$

$$J_{F2} = P_{H_2CO_3}^{apo/cyt} * ([H_2CO_3]_{apo} - [H_2CO_3]_{cyt}) * SA_{multiplier} \tag{220}$$

$$J_{F3} = P_{HCO_3^+} * ([HCO_3^+]_{apo} - [HCO_3^+]_{cyt}) * SA_{multiplier} \tag{221}$$

$$J_{F5} = P_{O_2}^{cyt/str} * ([O_2]_{cyt} - [O_2]_{str}) \tag{222}$$

$$J_{F6} = P_{CO_2}^{cyt/str} * ([CO_2]_{cyt} - [CO_2]_{str}) \tag{223}$$

$$J_{F7} = P_{H_2CO_3}^{cyt/str} * ([H_2CO_3]_{cyt} * [H_2CO_3]_{str}) \tag{224}$$

$$J_{F8} = P_{HCO_3^+}^{cyt/str} * ([HCO_3^+]_{cyt} * [HCO_3^+]_{str}) \tag{225}$$

$$J_{F9} = P_{PhotorespiratoryIntermediate} * [PhotorespiratoryIntermediate] \tag{226}$$

$$J_{R0} = \frac{[HCO_3^+]_{apo} * V_{max}^{BicA}}{[HCO_3^+]_{apo} + Km^{BicA}} * SA_{multiplier} \tag{227}$$

$$J_{R1} = \frac{[HCO_3^+]_{cyt} * V_{max}^{BicA}}{[HCO_3^+]_{cyt} + Km^{BicA}} \tag{228}$$

$$J_{R10} = \frac{Rubisco^{Vo}[O_2]_{str}}{[O_2]_{str} + (Rubisco_{O_2}^{Km}(1 + \frac{[CO_2]_{str}}{Rubisco_{CO_2}^{Km}}} \tag{229}$$

$$J_{R11} = \frac{Rubisco^{Vc}[CO_2]_{str}}{[CO_2]_{str} + (Rubisco_{CO_2}^{Km}(1 + \frac{[O_2]_{str}}{Rubisco_{O_2}^{Km}}} \tag{230}$$

$$J_{F9} = P_{PhotorespiratoryIntermediate} * [PhotorespiratoryIntermediate]_{cyt} \tag{231}$$

$$J_{R13} = J_{R11} \tag{232}$$

$$J_{R14} = \frac{6.022e23 * IAS * ([O_2]_{apo} - ([O_2]_{sub} \frac{HenryConstant^{O_2}}{1e21}}{SA_{apo}(Resistance_{air} + Resistance_{CW})} \tag{233}$$

$$J_{R15} = \frac{6.022e23 * IAS * ([CO_2]_{apo} - ([CO_2]_{sub} \frac{HenryConstant^{CO_2}}{1e21}}{SA_{apo}(Res_{air} + Res_{CW})} \tag{234}$$

$$J_{R16} = 0.5 J_{R10} \tag{235}$$

$$J_{R18} = \frac{StomatalConductance_{multiplier} StomatalConductance([O_2]_{ext} - [O_2]_{sub}}{Molarity_{air}} \tag{236}$$

$$J_{R19} = \frac{StomatalConductance_{multiplier} StomatalConductance([CO_2]_{ext} - [CO_2]_{sub}}{Molarity_{air}} \quad (237)$$

$$J_{R2} = K_f^{hydration}[CO_2]_{apo} - K_r^{dehydration}[H_2CO_3]_{apo} \quad (238)$$

$$J_{R3} = K_f^{deprotonation}[H_2CO_3]_{apo} - K_r^{protonation}[HCO_3^+]_{apo}[H^+]_{apo} \quad (239)$$

$$J_{R4} = K_f^{hydration}[CO_2]_{cyt} - K_r^{dehydration}[H_2CO_3]_{cyt} \quad (240)$$

$$J_{R5} = K_f^{deprotonation}[H_2CO_3]_{cyt} - K_r^{protonation}[HCO_3^+]_{cyt}[H^+]_{cyt} \quad (241)$$

$$J_{R6} = K_f^{hydration}[CO_2]_{str} - K_r^{dehydration}[H_2CO_3]_{str} \quad (242)$$

$$J_{R7} = K_f deprotonation[H_2CO_3]_{str} - K_r^{protonation}[HCO_3^+]_{str}[H^+]_{str} \quad (243)$$

$$J_{R8} = \frac{CA^{kcat}[CA]_{str}([CO_2]_{str} - \frac{([HCO_3^+]_{str}[H^+]_{str})}{Keq_{str}}}{CA^{km} + \frac{[HCO_3^+]_{str} CA^{Km}_{CO_2}}{CA^{Km}_{HCO_3^+}} + [CO_2]_{str}} \quad (244)$$

$$J_{R9} = \frac{CA^{kcat}[CA]_{cyt}([CO_2]_{cyt} - \frac{([HCO_3^+]_{cyt}[H^+]_{cyt})}{Keq_{cyt}}}{CA^{km} + \frac{[HCO_3^+]_{cyt} CA^{Km}_{CO_2}}{CA^{Km}_{HCO_3^+}} + [CO_2]_{cyt}} \quad (245)$$

2.5 Aggregated Processes in Figure S1

$$A_1 = [R12, R16, F9] \quad (246)$$

3 Algae with PCCM Model

3.1 Parameter Derivations

$$Res^{O_2}_{boundary} = (\frac{D_{O_2} * SA_{boundary}}{Thickness_{boundary}})^{-1} \quad (247)$$

$$Res^{O_2}_{apoplast} = (EffectivePorosity_{algae}(\frac{D_{O_2} * SA_{boundary}}{Thickness_{apoplast}}))^{-1} \quad (248)$$

$$Res^{CO_2}_{boundary} = (\frac{D_{CO_2} * SA_{boundary}}{Thickness_{boundary}})^{-1} \quad (249)$$

$$Res^{CO_2}_{apoplast} = (EffectivePorosity_{algae}(\frac{D_{CO_2} * SA_{boundary}}{Thickness_{apoplast}}))^{-1} \quad (250)$$

$$Res^{HCO_3^+}_{boundary} = (\frac{D_{HCO_3^+} * SA_{boundary}}{Thickness_{boundary}})^{-1} \quad (251)$$

$$Res^{HCO_3^+}_{apoplast} = (EffectivePorosity_{algae}(\frac{D_{HCO_3^+} * SA_{boundary}}{Thickness_{apoplast}}))^{-1} \tag{252}$$

$$Res^{H_2CO_3}_{boundary} = (\frac{D_{H_2CO_3} * SA_{boundary}}{Thickness_{boundary}})^{-1} \tag{253}$$

$$Res^{H_2CO_3}_{apoplast} = (EffectivePorosity_{algae}(\frac{D_{H_2CO_3} * SA_{boundary}}{Thickness_{apoplast}}))^{-1} \tag{254}$$

$$SA_{pyrenoid} = 4\pi r^2_{pyrenoid} \tag{255}$$

$$SA_{thylakoid} = \pi r^2_{thylakoid} height_{thylakoid} \tag{256}$$

$$SA_{chloroplast} = 4\pi r^2_{chloroplast} \tag{257}$$

$$SA_{cytosol} = 4\pi r^2_{cytosol} \tag{258}$$

$$SA_{plasmalemmasurface} = 2\pi r^2_{thylakoid} + 2\pi r_{thylakoid} height_{thylakoid} \tag{259}$$

$$Vol_{pyrenoid} = \frac{4}{3}\pi r^3_{pyrenoid} \tag{260}$$

$$Vol_{thylakoid} = \pi r^2_{thylakoid} height_{thylakoid} \tag{261}$$

$$Vol_{chloroplast} = \frac{4}{3}\pi r^3_{chloroplast} \tag{262}$$

$$Vol_{cytosol} = \frac{4}{3}\pi r^3_{cytosol} \tag{263}$$

$$Vol_{plasmalemmasurface} = \frac{4}{3}\pi r^3_{plasmalemmasurface} \tag{264}$$

$$[H^+]_{cyt} = 10^{-pH_{cyt}} \tag{265}$$

$$[H^+]_{str} = 10^{-pH_{str}} \tag{266}$$

$$[H^+]_{lum} = 10^{-pH_{lum}} \tag{267}$$

$$D^{O_2}_{plasmalemmasurface} = D_{O_2} \tag{268}$$

$$D^{O_2}_{cyt} = \frac{D^{O_2}}{Visc_{cytosol}} \tag{269}$$

$$D^{O_2}_{thy} = \frac{D^{O_2}}{Visc_{thylakoid}} \tag{270}$$

$$D^{O_2}_{str} = \frac{D^{O_2}}{Visc_{stroma}} \tag{271}$$

$$D^{O_2}_{pyr} = \frac{D^{O_2}}{Visc_{pyrenoid}} \tag{272}$$

$$D^{CO_2}_{plasmalemmasurface} = D_{CO_2} \tag{273}$$

$$D^{CO_2}_{cyt} = \frac{D^{CO_2}}{Visc_{cytosol}} \tag{274}$$

$$D^{CO_2}_{thy} = \frac{D^{CO_2}}{Visc_{thylakoid}} \tag{275}$$

$$D^{CO_2}_{str} = \frac{D^{CO_2}}{Visc_{stroma}} \tag{276}$$

$$D^{CO_2}_{pyr} = \frac{D^{CO_2}}{Visc_{pyrenoid}} \tag{277}$$

$$D^{H_2CO_3}_{plasmalemmasurface} = D_{H_2CO_3} \tag{278}$$

$$D^{H_2CO_3}_{cyt} = \frac{D^{H_2CO_3}}{Visc_{cytosol}} \tag{279}$$

$$D^{H_2CO_3}_{thy} = \frac{D^{H_2CO_3}}{Visc_{thylakoid}} \tag{280}$$

$$D^{H_2CO_3}_{str} = \frac{D^{H_2CO_3}}{Visc_{stroma}} \tag{281}$$

$$D^{H_2CO_3}_{pyr} = \frac{D^{H_2CO_3}}{Visc_{pyrenoid}} \tag{282}$$

$$D^{HCO_3}_{plasmalemmasurface} = D_{HCO_3} \tag{283}$$

$$D^{HCO_3}_{cyt} = \frac{D^{HCO_3}}{Visc_{cytosol}} \tag{284}$$

$$D^{HCO_3}_{thy} = \frac{D^{HCO_3}}{Visc_{thylakoid}} \tag{285}$$

$$D^{HCO_3}_{str} = \frac{D^{HCO_3}}{Visc_{stroma}} \tag{286}$$

$$D^{HCO_3}_{pyr} = \frac{D^{HCO_3}}{Visc_{pyrenoid}} \tag{287}$$

3.2 Partial Differential Equations for Volume Concentrations

3.2.1 Plasmalemma Surface

$$\frac{\partial [O_2]_{pla}(x)}{\partial t} = D_{apo}^{O_2} \nabla^2 [O_2]_{apo} + Lumped J_{r0} \tag{288}$$

$$\frac{\partial [CO_2]_{pla}(x)}{\partial t} = D_{apo}^{CO_2} \nabla^2 [CO_2]_{apo} + Lumped J_{r1} - J_{r16} \tag{289}$$

$$\frac{\partial [H_2CO_3]_{pla}(x)}{\partial t} = D_{apo}^{H_2CO_3} \nabla^2 [H_2CO_3]_{apo} + Lumped J_{r2} + J_{r16} - J_{r17} \tag{290}$$

$$\frac{\partial [HCO_3^+]_{pla}(x)}{\partial t} = D_{apo}^{HCO_3^+} \nabla^2 [HCO_3^+]_{apo} + Lumped J_{r3} + J_{r17} \tag{291}$$

3.2.2 Cytosol

$$\frac{\partial [O_2]_{cyt}(x)}{\partial t} = D_{cyt}^{O_2} \nabla^2 [O_2]_{cyt} \tag{292}$$

$$\frac{\partial [CO_2]_{cyt}(x)}{\partial t} = D_{cyt}^{CO_2} \nabla^2 [CO_2]_{cyt} - J_{r4} - J_{r13} \tag{293}$$

$$\frac{\partial [H_2CO_3]_{cyt}(x)}{\partial t} = D_{cyt}^{H_2CO_3} \nabla^2 [H_2CO_3]_{cyt} + J_{r4} - J_{r13} \tag{294}$$

$$\frac{\partial [HCO_3^+]_{cyt}(x)}{\partial t} = D_{cyt}^{HCO_3^+} \nabla^2 [HCO_3^+]_{cyt} + J_{r5} + J_{r13} \tag{295}$$

3.2.3 Stroma

$$\frac{\partial [O_2]_{str}(x)}{\partial t} = D_{str}^{O_2} \nabla^2 [O_2]_{str} \tag{296}$$

$$\frac{\partial [CO_2]_{str}(x)}{\partial t} = D_{str}^{CO_2} \nabla^2 [CO_2]_{str} - J_{r8} - J_{r22} \tag{297}$$

$$\frac{\partial [H_2CO_3]_{str}(x)}{\partial t} = D_{str}^{H_2CO_3} \nabla^2 [H_2CO_3]_{str} + J_{r8} - J_{r9} \tag{298}$$

$$\frac{\partial [HCO_3^+]_{str}(x)}{\partial t} = D_{str}^{HCO_3^+} \nabla^2 [HCO_3^+]_{str} + J_{r9} + J_{r22} \tag{299}$$

3.2.4 Lumen

$$\frac{\partial[O_2]_{lum}(x)}{\partial t} = D_{lum}^{O_2}\nabla^2[O_2]_{lum} \tag{300}$$

$$\frac{\partial[CO_2]_{lum}(x)}{\partial t} = D_{lum}^{CO_2}\nabla^2[CO_2]_{lum} - J_{r20} \tag{301}$$

$$\frac{\partial[H_2CO_3]_{lum}(x)}{\partial t} = D_{lum}^{H_2CO_3}\nabla^2[H_2CO_3]_{lum} + J_{r20} - J_{r21} \tag{302}$$

$$\frac{\partial[HCO_3^+]_{lum}(x)}{\partial t} = D_{lum}^{HCO_3^+}\nabla^2[HCO_3^+]_{lum} + J_{r21} \tag{303}$$

3.2.5 Pyrenoid

$$\frac{\partial[O_2]_{pyr}(x)}{\partial t} = D_{pyr}^{O_2}\nabla^2[O_2]_{pyr} - J_{r17} \tag{304}$$

$$\frac{\partial[CO_2]_{pyr}(x)}{\partial t} = D_{pyr}^{CO_2}\nabla^2[CO_2]_{pyr} - J_{r10} - J_{r12} - J_{r15} \tag{305}$$

$$\frac{\partial[H_2CO_3]_{pyr}(x)}{\partial t} = D_{pyr}^{H_2CO_3}\nabla^2[H_2CO_3]_{pyr} + J_{r10} - J_{r11} \tag{306}$$

$$\frac{\partial[HCO_3^+]_{pyr}(x)}{\partial t} = D_{pyr}^{HCO_3^+}\nabla^2[HCO_3^+]_{pyr} + J_{r11} + J_{r15} \tag{307}$$

3.3 Boundary Conditions

3.3.1 Pyrenoid to Lumen

$$\frac{\partial[O_2]_{pyr}(x)}{\partial t} = J_{r20}\forall x \in \delta\Omega \tag{308}$$

$$\frac{\partial[CO_2]_{pyr}(x)}{\partial t} = J_{r21}\forall x \in \delta\Omega \tag{309}$$

$$\frac{\partial[H_2CO_3]_{pyr}(x)}{\partial t} = J_{r22}\forall x \in \delta\Omega \tag{310}$$

$$\frac{\partial[HCO_3^+]_{pyr}(x)}{\partial t} = J_{r23} + J_r25\forall x \in \delta\Omega \tag{311}$$

$$-\frac{\partial[O_2]_{lum}(x)}{\partial t} = J_{r20}\forall x \in \delta\Omega \tag{312}$$

$$-\frac{\partial[CO_2]_{lum}(x)}{\partial t} = J_{r21}\forall x \in \delta\Omega \tag{313}$$

$$-\frac{\partial[H_2CO_3]_{lum}(x)}{\partial t} = J_{r22}\forall x \in \delta\Omega \tag{314}$$

$$-\frac{\partial[HCO_3^+]_{lum}(x)}{\partial t} = J_{r23} + J_r25\forall x \in \delta\Omega \tag{315}$$

3.3.2 Pyrenoid to Stroma

$$\frac{\partial [O_2]_{pyr}(x)}{\partial t} = J_{r8} + J_{r17} \forall x \in \delta\Omega \tag{316}$$

$$\frac{\partial [CO_2]_{pyr}(x)}{\partial t} = J_{r9} \forall x \in \delta\Omega \tag{317}$$

$$\frac{\partial [H_2CO_3]_{pyr}(x)}{\partial t} = J_{r10} \forall x \in \delta\Omega \tag{318}$$

$$\frac{\partial [HCO_3^+]_{pyr}(x)}{\partial t} = J_{r11} \forall x \in \delta\Omega \tag{319}$$

$$-\frac{\partial [O_2]_{lum}(x)}{\partial t} = J_{r8} + J_{r17} \forall x \in \delta\Omega \tag{320}$$

$$-\frac{\partial [CO_2]_{lum}(x)}{\partial t} = J_{r9} \forall x \in \delta\Omega \tag{321}$$

$$-\frac{\partial [H_2CO_3]_{lum}(x)}{\partial t} = J_{r10} \forall x \in \delta\Omega \tag{322}$$

$$-\frac{\partial [HCO_3^+]_{lum}(x)}{\partial t} = J_{r11} \forall x \in \delta\Omega \tag{323}$$

3.3.3 Lumen to Stroma

$$\frac{\partial [O_2]_{lum}(x)}{\partial t} = J_{F12} \forall x \in \delta\Omega \tag{324}$$

$$\frac{\partial [CO_2]_{lum}(x)}{\partial t} = J_{F13} \forall x \in \delta\Omega \tag{325}$$

$$\frac{\partial [H_2CO_3]_{lum}(x)}{\partial t} = J_{F14} \forall x \in \delta\Omega \tag{326}$$

$$\frac{\partial [HCO_3^+]_{lum}(x)}{\partial t} = -J_{F15} - J_{F24} \forall x \in \delta\Omega \tag{327}$$

$$-\frac{\partial [O_2]_{str}(x)}{\partial t} = J_{F12} \forall x \in \delta\Omega \tag{328}$$

$$-\frac{\partial [CO_2]_{str}(x)}{\partial t} = J_{F13} \forall x \in \delta\Omega \tag{329}$$

$$-\frac{\partial [H_2CO_3]_{str}(x)}{\partial t} = J_{F14} \forall x \in \delta\Omega \tag{330}$$

$$-\frac{\partial [HCO_3^+]_{str}(x)}{\partial t} = J_{F15} + J_{F24} \forall x \in \delta\Omega \tag{331}$$

3.3.4 Stroma to Cytosol

$$\frac{\partial[O_2]_{str}(x)}{\partial t} = J_{F4} \forall x \in \delta\Omega \tag{332}$$

$$\frac{\partial[CO_2]_{str}(x)}{\partial t} = J_{F5} \forall x \in \delta\Omega \tag{333}$$

$$\frac{\partial[H_2CO_3]_{str}(x)}{\partial t} = J_{F6} \forall x \in \delta\Omega \tag{334}$$

$$\frac{\partial[HCO_3^+]_{str}(x)}{\partial t} = J_{F7} + J_{F16} + J_{r18} \forall x \in \delta\Omega \tag{335}$$

$$-\frac{\partial[O_2]_{cyt}(x)}{\partial t} = J_{F4} \forall x \in \delta\Omega \tag{336}$$

$$-\frac{\partial[CO_2]_{cyt}(x)}{\partial t} = J_{F5} \forall x \in \delta\Omega \tag{337}$$

$$-\frac{\partial[H_2CO_3]_{cyt}(x)}{\partial t} = J_{F6} \forall x \in \delta\Omega \tag{338}$$

$$-\frac{\partial[HCO_3^+]_{cyt}(x)}{\partial t} = J_{F7} + J_{F16} + J_{r18} \forall x \in \delta\Omega \tag{339}$$

3.3.5 Cytosol to Plasmalemma Surface

$$\frac{\partial[O_2]_{cyt}(x)}{\partial t} = J_{F0} \forall x \in \delta\Omega \tag{340}$$

$$\frac{\partial[CO_2]_{cyt}(x)}{\partial t} = J_{F1} \forall x \in \delta\Omega \tag{341}$$

$$\frac{\partial[H_2CO_3]_{cyt}(x)}{\partial t} = J_{F2} \forall x \in \delta\Omega \tag{342}$$

$$\frac{\partial[HCO_3^+]_{cyt}(x)}{\partial t} = -J_{F3} - J_{F19} \forall x \in \delta\Omega \tag{343}$$

$$-\frac{\partial[O_2]_{apo}(x)}{\partial t} = J_{F0} \forall x \in \delta\Omega \tag{344}$$

$$-\frac{\partial[CO_2]_{apo}(x)}{\partial t} = J_{F1} \forall x \in \delta\Omega \tag{345}$$

$$-\frac{\partial[H_2CO_3]_{apo}(x)}{\partial t} = J_{F2} \forall x \in \delta\Omega \tag{346}$$

$$-\frac{\partial[HCO_3^+]_{apo}(x)}{\partial t} = -J_{F3} - J_{F19} \forall x \in \delta\Omega \tag{347}$$

3.4 Fluxes

$$J_{F0} = P_{O_2}^{pla/cyt} * ([O_2]_{pla} - [O_2]_{cyt}) \tag{348}$$

$$J_{F1} = P_{CO_2}^{pla/cyt} * ([CO_2]_{pla} - [CO_2]_{cyt}) \tag{349}$$

$$J_{F10} = P_{H_2CO_3}^{str/pyr} * ([H_2CO_3]_{str} - [H_2CO_3]_{pyr}) \tag{350}$$

$$J_{F11} = P_{HCO_3^+}^{str/pyr} * ([HCO_3^+]_{str} - [HCO_3^+]_{pyr}) \tag{351}$$

$$J_{F12} = P_{O_2}^{str/thy} * ([O_2]_{str} - [O_2]_{lum}) \tag{352}$$

$$J_{F13} = P_{CO_2}^{str/thy} * ([CO_2]_{str} - [CO_2]_{lum}) \tag{353}$$

$$J_{F14} = P_{H_2CO_3}^{str/thy} * ([H_2CO_3]_{str} - [H_2CO_3]_{lum}) \tag{354}$$

$$J_{F15} = P_{HCO_3^+}^{str/thy} * ([HCO_3^+]_{str} * [HCO_3^+]_{lum}) \tag{355}$$

$$J_{F16} = P_{HCO_3^+/facilitated}^{str/thy} * ([HCO_3^+]_{str} * [HCO_3^+]_{lum}) \tag{356}$$

$$J_{F17} = P_{O_2}^{intermediate} * [O_2]^{intermediate} \tag{357}$$

$$J_{F18} = P_{PhotorespiratoryIntermediate} * [PhotorespiratoryIntermediate] \tag{358}$$

$$J_{F19} = P_{PhotorespiratoryIntermediate} * [PhotorespiratoryIntermediate]_{stroma} \tag{359}$$

$$J_{F2} = P_{H_2CO_3}^{pla/cyt} * ([H_2CO_3]_{pla} - [H_2CO_3]_{cyt}) \tag{360}$$

$$J_{F20} = P_{O_2}^{thy/pyr} * ([O_2]_{lum} - [O_2]_{pyr}) \tag{361}$$

$$J_{F21} = P_{CO_2}^{thy/pyr} * ([CO_2]_{lum} - [CO_2]_{pyr}) \tag{362}$$

$$J_{F22} = P_{H_2CO_3}^{thy/pyr} * ([H_2CO_3]_{lum} - [H_2CO_3]_{pyr}) \tag{363}$$

$$J_{F23} = P_{HCO_3^+}^{thy/pyr} * ([HCO_3^+]_{lum} * [HCO_3^+]_{pyr}) \tag{364}$$

$$J_{F24} = P_{HCO_3^+/facilitated}^{str/thy} * ([HCO_3^+]_{str} * [HCO_3^+]_{lum}) \tag{365}$$

$$J_{F25} = P_{HCO_3^+/facilitated}^{thy/pyr} * ([HCO_3^+]_{lum} * [HCO_3^+]_{pyr}) \tag{366}$$

$$J_{F3} = P_{HCO_3^+}^{pla/cyt} * ([HCO_3^+]_{pla} * [HCO_3^+]_{cyt}) \tag{367}$$

$$J_{F4} = \frac{P_{O_2}}{2}^{cyt/str} * ([O_2]_{cyt} * [O_2]_{str}) \tag{368}$$

$$J_{F5} = \frac{P_{CO_2}}{2}^{cyt/str} * ([O_2]_{cyt} * [O_2]_{str}) \tag{369}$$

$$J_{F6} = \frac{P_{H_2CO_3}}{2}^{cyt/str} * ([H_2CO_3]_{cyt} * [H_2CO_3]_{str}) \tag{370}$$

$$J_{F7} = \frac{P_{HCO_3^+}}{2}^{cyt/str} * ([HCO_3^+]_{cyt} * [HCO_3^+]_{str}) \tag{371}$$

$$J_{F8} = P_{O_2}^{str/pyr} * ([O_2]_{str} * [O_2]_{pyr}) \tag{372}$$

$$J_{F9} = P_{CO_2}^{str/pyr} * ([CO_2]_{str} * [CO_2]_{pyr}) \tag{373}$$

$$J_{R10} = K_f^{hydration}[CO_2]_{pyr} - K_r^{dehydration}[H_2CO_3]_{pyr} \tag{374}$$

$$J_{R11} = K_f^{deprotonation}[H_2CO_3]_{pyr} - K_r^{protonation}[HCO_3^+]_{pyr}[H^+]_{pyr} \tag{375}$$

$$J_{R12} = \frac{Rubisco^{Vc}[CO_2]_{pyr}}{[CO_2]_{pyr} + (Rubisco_{CO_2}^{Km}(1 + \frac{[O_2]_{pyr}}{Rubisco_{O_2}^{Km}}} \tag{376}$$

$$J_{R13} = \frac{CA^{kcat}[CA]_{cyt}([CO_2]_{cyt} - \frac{([HCO_3^+]_{cyt}[H^+]_{cyt})}{Keq_{cyt}}}{CA^{km} + \frac{[HCO_3^+]_{cyt}CA_{CO_2}^{Km}}{CA_{HCO_3^+}^{Km}} + [CO_2]_{cyt}} \tag{377}$$

$$J_{R15} = \frac{CA^{kcat}[CA]_{pyr}([CO_2]_{pyr} - \frac{([HCO_3^+]_{pyr}[H^+]_{pyr})}{Keq_{pyr}}}{CA^{km} + \frac{[HCO_3^+]_{pyr}CA_{CO_2}^{Km}}{CA_{HCO_3^+}^{Km}} + [CO_2]_{pyr}} \tag{378}$$

$$J_{R16} = K_f^{hydration}[CO_2]_{pla} - K_r^{dehydration}[H_2CO_3]_{pla} \tag{379}$$

$$J_{R17} = K_f^{deprotonation}[H_2CO_3]_{pla} - K_r^{protonation}[HCO_3^+]_{pla}[H^+]_{pla} \tag{380}$$

$$J_{R18} = \frac{[HCO_3^+]_{cyt} * V_{max}^{BicA}}{[HCO_3^+]_{cyt} + Km^{BicA}} \tag{381}$$

$$J_{R19} = \frac{[HCO_3^+]_{pla} * V_{max}^{BicA}}{[HCO_3^+]_{pla} + Km^{BicA}} \tag{382}$$

$$J_{R20} = K_f^{hydration}[CO_2]_{lum} - K_r^{dehydration}[H_2CO_3]_{lum} \tag{383}$$

$$J_{R21} = K_f^{deprotonation}[H_2CO_3]_{lum} - K_r^{protonation}[HCO_3^+]_{lum}[H^+]_{lum} \tag{384}$$

$$J_{R22} = \frac{CA^{kcat}[CA]_{str}([CO_2]_{str} - \frac{([HCO_3^+]_{str}[H^+]_{pyr})}{Keq_{str}}}{CA^{km} + \frac{[HCO_3^+]_{str}CA_{CO_2}^{Km}}{CA_{HCO_3^+}^{Km}} + [CO_2]_{str}} \tag{385}$$

$$J_{R4} = K_f^{hydration}[CO_2]_{cyt} - K_r^{dehydration}[H_2CO_3]_{cyt} \tag{386}$$

$$J_{R5} = K_f^{deprotonation}[H_2CO_3]_{cyt} - K_r^{protonation}[HCO_3^+]_{cyt}[H^+]_{cyt} \tag{387}$$

$$J_{R6} = 0.5J_{R7} \tag{388}$$

$$J_{R7} = \frac{Rubisco^{Vo}[O_2]_{pyr}}{[O_2]_{pyr} + (Rubisco^{Km}_{O_2}(1 + \frac{[CO_2]_{pyr}}{Rubisco^{Km}_{CO_2}}} \tag{389}$$

$$J_{R8} = K_f^{hydration}[CO_2]_{str} - K_r^{dehydration}[H_2CO_3]_{str} \tag{390}$$

$$J_{R9} = K_f^{deprotonation}[H_2CO_3]_{str} - K_r^{protonation}[HCO_3^+]_{str}[H^+]_{str} \tag{391}$$

$$J_{E0} = \frac{([O_2]_{ext} - [O_2]_{pla})}{(Resistance^{O_2}_{boundary} + Resistance^{O_2}_{CW})} * (\frac{6.022e23}{1e21}) \tag{392}$$

$$J_{E1} = \frac{(DIC * [CO_2]_{ext}) - [CO_2]_{pla})}{(Resistance^{CO_2}_{boundary} + Resistance^{CO_2}_{CW})} * (\frac{6.022e23}{1e21}) \tag{393}$$

$$J_{E2} = \frac{(DIC * [H_2CO_3]_{ext}) - [H_2CO_3]_{pla})}{(Resistance^{H_2CO_3}_{boundary} + Resistance^{H_2CO_3}_{CW})} * (\frac{6.022e23}{1e21}) \tag{394}$$

$$J_{E3} = \frac{(DIC * [HCO_3^+]_{ext}) - [HCO_3^+]_{pla})}{(Resistance^{HCO_3^+}_{boundary} + Resistance^{HCO_3^+}_{CW})} * (\frac{6.022e23}{1e21}) \tag{395}$$

3.5 Aggregated Processes in Figure S1

$$A_1 = [R6, F18, F19] \tag{396}$$

$$A_2 = [F17, R12] \tag{397}$$

4 Algae without PCCM Model

4.1 Parameter Derivations

$$Res^{O_2}_{boundary} = (\frac{D_{O_2} * SA_{boundary}}{Thickness_{boundary}})^{-1} \tag{398}$$

$$Res^{O_2}_{CW} = (EffectivePorosity_{algae}(\frac{D_{O_2} * SA_{boundary}}{Thickness_{CW}}))^{-1} \tag{399}$$

$$Res^{CO_2}_{boundary} = (\frac{D_{CO_2} * SA_{boundary}}{Thickness_{boundary}})^{-1} \tag{400}$$

$$Res^{CO_2}_{CW} = (EffectivePorosity_{algae}(\frac{D_{CO_2} * SA_{boundary}}{Thickness_{CW}}))^{-1} \tag{401}$$

$$Res^{HCO_3^+}_{boundary} = (\frac{D_{HCO_3^+} * SA_{boundary}}{Thickness_{boundary}})^{-1} \tag{402}$$

$$Res_{CW}^{HCO_3^+} = (EffectivePorosity_{algae}(\frac{D_{HCO_3^+} * SA_{boundary}}{Thickness_{CW}}))^{-1} \tag{403}$$

$$Res_{boundary}^{H_2CO_3} = (\frac{D_{H_2CO_3} * SA_{boundary}}{Thickness_{boundary}})^{-1} \tag{404}$$

$$Res_{CW}^{H_2CO_3} = (EffectivePorosity_{algae}(\frac{D_{H_2CO_3} * SA_{boundary}}{Thickness_{CW}}))^{-1} \tag{405}$$

$$SA_{chloroplast} = 4\pi r_{chloroplast}^2 \tag{406}$$

$$SA_{cytosol} = 4\pi r_{cytosol}^2 \tag{407}$$

$$Vol_{chloroplast} = \frac{4}{3}\pi r_{chloroplast}^3 \tag{408}$$

$$Vol_{cytosol} = \frac{4}{3}\pi r_{cytosol}^3 \tag{409}$$

$$Vol_{plasmalemmasurface} = \frac{4}{3}\pi r_{plasmalemmasurface}^3 \tag{410}$$

$$[H^+]_{cyt} = 10^{-pH_{cyt}} \tag{411}$$

$$[H^+]_{str} = 10^{-pH_{str}} \tag{412}$$

$$[H^+]_{lum} = 10^{-pH_{lum}} \tag{413}$$

$$D_{plasmalemmasurface}^{O_2} = D_{O_2} \tag{414}$$

$$D_{cyt}^{O_2} = \frac{D^{O_2}}{Visc_{cytosol}} \tag{415}$$

$$D_{str}^{O_2} = \frac{D^{O_2}}{Visc_{stroma}} \tag{416}$$

$$D_{plasmalemmasurface}^{CO_2} = D_{CO_2} \tag{417}$$

$$D_{cyt}^{CO_2} = \frac{D^{CO_2}}{Visc_{cytosol}} \tag{418}$$

$$D_{str}^{CO_2} = \frac{D^{CO_2}}{Visc_{stroma}} \tag{419}$$

$$D_{plasmalemmasurface}^{H_2CO_3} = D_{H_2CO_3} \tag{420}$$

$$D_{cyt}^{H_2CO_3} = \frac{D^{H_2CO_3}}{Visc_{cytosol}} \tag{421}$$

$$D_{str}^{H_2CO_3} = \frac{D^{H_2CO_3}}{Visc_{stroma}} \tag{422}$$

$$D^{HCO_3}_{plasmalemmasurface} = D_{HCO_3} \tag{423}$$

$$D^{HCO_3}_{cyt} = \frac{D^{HCO_3}}{Visc_{cytosol}} \tag{424}$$

$$D^{HCO_3}_{str} = \frac{D^{HCO_3}}{Visc_{stroma}} \tag{425}$$

4.2 Partial Differential Equations for Volume Concentrations

4.2.1 Plasmalemma Surface

$$\frac{\partial[O_2]_{pla}(x)}{\partial t} = D^{O_2}_{pla}\nabla^2[O_2]_{pla} + LumpedJ_{r0} \tag{426}$$

$$\frac{\partial[CO_2]_{pla}(x)}{\partial t} = D^{CO_2}_{pla}\nabla^2[CO_2]_{pla} + LumpedJ_{r1} - J_{r16} \tag{427}$$

$$\frac{\partial[H_2CO_3]_{pla}(x)}{\partial t} = D^{H_2CO_3}_{pla}\nabla^2[H_2CO_3]_{pla} + LumpedJ_{r2} + J_{r16} - J_{r17} \tag{428}$$

$$\frac{\partial[HCO_3^+]_{pla}(x)}{\partial t} = D^{HCO_3^+}_{pla}\nabla^2[HCO_3^+]_{pla} + LumpedJ_{r3} + J_{r17} \tag{429}$$

4.2.2 Cytosol

$$\frac{\partial[O_2]_{cyt}(x)}{\partial t} = D^{O_2}_{cyt}\nabla^2[O_2]_{cyt} \tag{430}$$

$$\frac{\partial[CO_2]_{cyt}(x)}{\partial t} = D^{CO_2}_{cyt}\nabla^2[CO_2]_{cyt} - J_{r4} - J_{r6} \tag{431}$$

$$\frac{\partial[H_2CO_3]_{cyt}(x)}{\partial t} = D^{H_2CO_3}_{cyt}\nabla^2[H_2CO_3]_{cyt} + J_{r4} - J_{r5} \tag{432}$$

$$\frac{\partial[HCO_3^+]_{cyt}(x)}{\partial t} = D^{HCO_3^+}_{cyt}\nabla^2[HCO_3^+]_{cyt} + J_{r5} + J_{r6} \tag{433}$$

4.2.3 Stroma

$$\frac{\partial[O_2]_{str}(x)}{\partial t} = D^{O_2}_{str}\nabla^2[O_2]_{str} + J_{oxy} - J_{r21} \tag{434}$$

$$\frac{\partial[CO_2]_{str}(x)}{\partial t} = D^{CO_2}_{str}\nabla^2[CO_2]_{str} - J_{r8} - J_{oxy} - J_{StromaCA} \tag{435}$$

$$\frac{\partial[H_2CO_3]_{str}(x)}{\partial t} = D^{H_2CO_3}_{str}\nabla^2[H_2CO_3]_{str} + J_{r8} - J_{r9} \tag{436}$$

$$\frac{\partial[HCO_3^+]_{str}(x)}{\partial t} = D^{HCO_3^+}_{str}\nabla^2[HCO_3^+]_{str} + J_{r9} + J_{StromaCA} \tag{437}$$

4.3 Boundary Conditions

4.3.1 Stroma to Cytosol

$$\frac{\partial[O_2]_{cyt}(x)}{\partial t} = J_{r4} \forall x \in \delta\Omega \tag{438}$$

$$\frac{\partial[CO_2]_{cyt}(x)}{\partial t} = J_{r5} + J_{r8} \forall x \in \delta\Omega \tag{439}$$

$$\frac{\partial[H_2CO_3]_{cyt}(x)}{\partial t} = J_{r6} \forall x \in \delta\Omega \tag{440}$$

$$\frac{\partial[HCO_3^+]_{cyt}(x)}{\partial t} = -J_{r7} - J_{r18} \forall x \in \delta\Omega \tag{441}$$

$$-\frac{\partial[O_2]_{str}(x)}{\partial t} = J_{r4} \forall x \in \delta\Omega \tag{442}$$

$$-\frac{\partial[CO_2]_{str}(x)}{\partial t} = J_{r5} + J_{r8} \forall x \in \delta\Omega \tag{443}$$

$$-\frac{\partial[H_2CO_3]_{str}(x)}{\partial t} = J_{r6} \forall x \in \delta\Omega \tag{444}$$

$$-\frac{\partial[HCO_3^+]_{str}(x)}{\partial t} = J_{r7} - J_{r18} \forall x \in \delta\Omega \tag{445}$$

4.3.2 Cytosol to Plasmalemma Surface

$$\frac{\partial[O_2]_{cyt}(x)}{\partial t} = J_{F0} \forall x \in \delta\Omega \tag{446}$$

$$\frac{\partial[CO_2]_{cyt}(x)}{\partial t} = J_{F1} \forall x \in \delta\Omega \tag{447}$$

$$\frac{\partial[H_2CO_3]_{cyt}(x)}{\partial t} = J_{F2} \forall x \in \delta\Omega \tag{448}$$

$$\frac{\partial[HCO_3^+]_{cyt}(x)}{\partial t} = J_{F3} + J_{r19} \forall x \in \delta\Omega \tag{449}$$

$$-\frac{\partial[O_2]_{apo}(x)}{\partial t} = J_{F0} \forall x \in \delta\Omega \tag{450}$$

$$-\frac{\partial[CO_2]_{apo}(x)}{\partial t} = J_{F1} \forall x \in \delta\Omega \tag{451}$$

$$-\frac{\partial[H_2CO_3]_{apo}(x)}{\partial t} = J_{F2} \forall x \in \delta\Omega \tag{452}$$

$$-\frac{\partial[HCO_3^+]_{apo}(x)}{\partial t} = J_{F3} + J_{r19} \forall x \in \delta\Omega \tag{453}$$

4.4 Fluxes

$$J_{F0} = P_{O_2}^{pla/cyt} * ([O_2]_{pla} - [O_2]_{cyt}) \tag{454}$$

$$J_{F1} = P_{CO_2}^{pla/cyt} * ([CO_2]_{pla} - [CO_2]_{cyt}) \tag{455}$$

$$J_{F2} = P_{H_2CO_3}^{pla/cyt} * ([H_2CO_3]_{pla} - [H_2CO_3]_{cyt}) \tag{456}$$

$$J_{F3} = P_{HCO_3^+}^{pla/cyt} * ([HCO_3^+]_{pla} - [HCO_3^+]_{cyt}) \tag{457}$$

$$J_{F4} = \frac{P_{O_2}^{cyt/str}}{2} * ([O_2]_{cyt} - [O_2]_{str}) \tag{458}$$

$$J_{F5} = \frac{P_{CO_2}^{cyt/str}}{2} * ([CO_2]_{cyt} - [CO_2]_{str}) \tag{459}$$

$$J_{F6} = \frac{P_{H_2CO_3}^{cyt/str}}{2} * ([H_2CO_3]_{cyt} - [H_2CO_3]_{str}) \tag{460}$$

$$J_{F7} = \frac{P_{HCO_3^+}^{cyt/str}}{2} * ([HCO_3^+]_{cyt} - [HCO_3^+]_{str}) \tag{461}$$

$$J_{F8} = P_{PhotorespiratoryIntermediate} * [PhotorespiratoryIntermediate] \tag{462}$$

$$J_{R16} = K_f^{hydration}[CO_2]_{pla} - K_r^{dehydration}[H_2CO_3]_{pla} \tag{463}$$

$$J_{R17} = K_f^{deprotonation}[H_2CO_3]_{pla} - K_r^{protonation}[HCO_3^+]_{pla}[H^+]_{pla} \tag{464}$$

$$J_{R18} = \frac{[HCO_3^+]_{cyt} * V_{max}^{BicA}}{[HCO_3^+]_{cyt} + Km^{BicA}} \tag{465}$$

$$J_{R19} = \frac{[HCO_3^+]_{pla} * V_{max}^{BicA}}{[HCO_3^+]_{pla} + Km^{BicA}} \tag{466}$$

$$J_{R21} = J_{r10} \tag{467}$$

$$J_{R4} = K_f^{hydration}[CO_2]_{cyt} - K_r^{dehydration}[H_2CO_3]_{cyt} \tag{468}$$

$$J_{R5} = K_f^{deprotonation}[H_2CO_3]_{cyt} - K_r^{protonation}[HCO_3^+]_{cyt}[H^+]_{cyt} \tag{469}$$

$$J_{R6} = \frac{CA^{kcat}[CA]_{cyt}([CO_2]_{cyt} - \frac{([HCO_3^+]_{cyt}[H^+]_{cyt})}{Keq_{cyt}}}{CA^{km} + \frac{[HCO_3^+]_{cyt}CA_{CO_2}^{Km}}{CA_{HCO_3^+}^{Km}} + [CO_2]_{cyt}} \tag{470}$$

$$J_{R7} = 0.5J_{R21} \tag{471}$$

$$J_{R8} = K_f^{hydration}[CO_2]_{str} - K_r^{dehydration}[H_2CO_3]_{str} \tag{472}$$

$$J_{R9} = K_f^{deprotonation}[H_2CO_3]_{str} - K_r^{dehydration}[HCO_3^+]_{str}[H^+]_{str} \tag{473}$$

$$J_{R10} = \frac{Rubisco^{Vc}[CO_2]_{str}}{[CO_2]_{str} + (Rubisco^{Km}_{CO_2}(1 + \frac{[O_2]_{str}}{Rubisco^{Km}_{O_2}}} \tag{474}$$

$$J_{R11} = \frac{Rubisco^{Vo}[O_2]_{str}}{[O_2]_{str} + (Rubisco^{Km}_{O_2}(1 + \frac{[CO_2]_{str}}{Rubisco^{Km}_{CO_2}}} \tag{475}$$

$$J_{R12} = \frac{CA^{kcat}_{StrCA}[CA]_{str}([CO_2]_{str} - \frac{([HCO_3^+]_{str}[H^+]_{str})}{Keq_{str}}}{CA^{km}_{StrCA} + \frac{[HCO_3^+]_{str}CA^{Km}_{CO_2}}{CA^{Km}_{HCO_3^+}} + [CO_2]_{str}} \tag{476}$$

$$J_{E0} = \frac{([O_2]_{ext} - [O_2]_{pla})}{(Res^{O_2}_{boundary} + Res^{O_2}_{CW})} * (\frac{6.022e23}{1e21}) \tag{477}$$

$$J_{E1} = \frac{((DIC * [CO_2]_{ext}) - [CO_2]_{pla})}{(Res^{CO_2}_{boundary} + Res^{CO_2}_{CW})} * (\frac{6.022e23}{1e21}) \tag{478}$$

$$J_{E2} = \frac{((DIC * [H_2CO_3]_{ext}) - [H_2CO_3]_{pla})}{(Res^{H_2CO_3}_{boundary} + Res^{H_2CO_3}_{CW})} * (\frac{6.022e23}{1e21}) \tag{479}$$

$$J_{E3} = \frac{((DIC * [HCO_3^+]_{ext}) - [HCO_3^+]_{pla})}{(Res^{HCO_3^+}_{boundary} + Res^{HCO_3^+}_{CW})} * (\frac{6.022e23}{1e21}) \tag{480}$$

4.5 Aggregated Processes in Figure S1

$$A_1 = [R17, F8] \tag{481}$$
