# Supplementary material for: Reaction-diffusion modeling provides insights into biophysical carbon-concentrating mechanisms in land plants
Source: Plant Physiol. 2024 Jun 10;196(2):1374–90. doi: 10.1093/plphys/kiae324 (PMC11444298; doi:10.1093/plphys/kiae324)
Supplement: kiae324_Supplementary_Data [file kiae324_supplementary_data.zip › Supplemental Data.pdf]

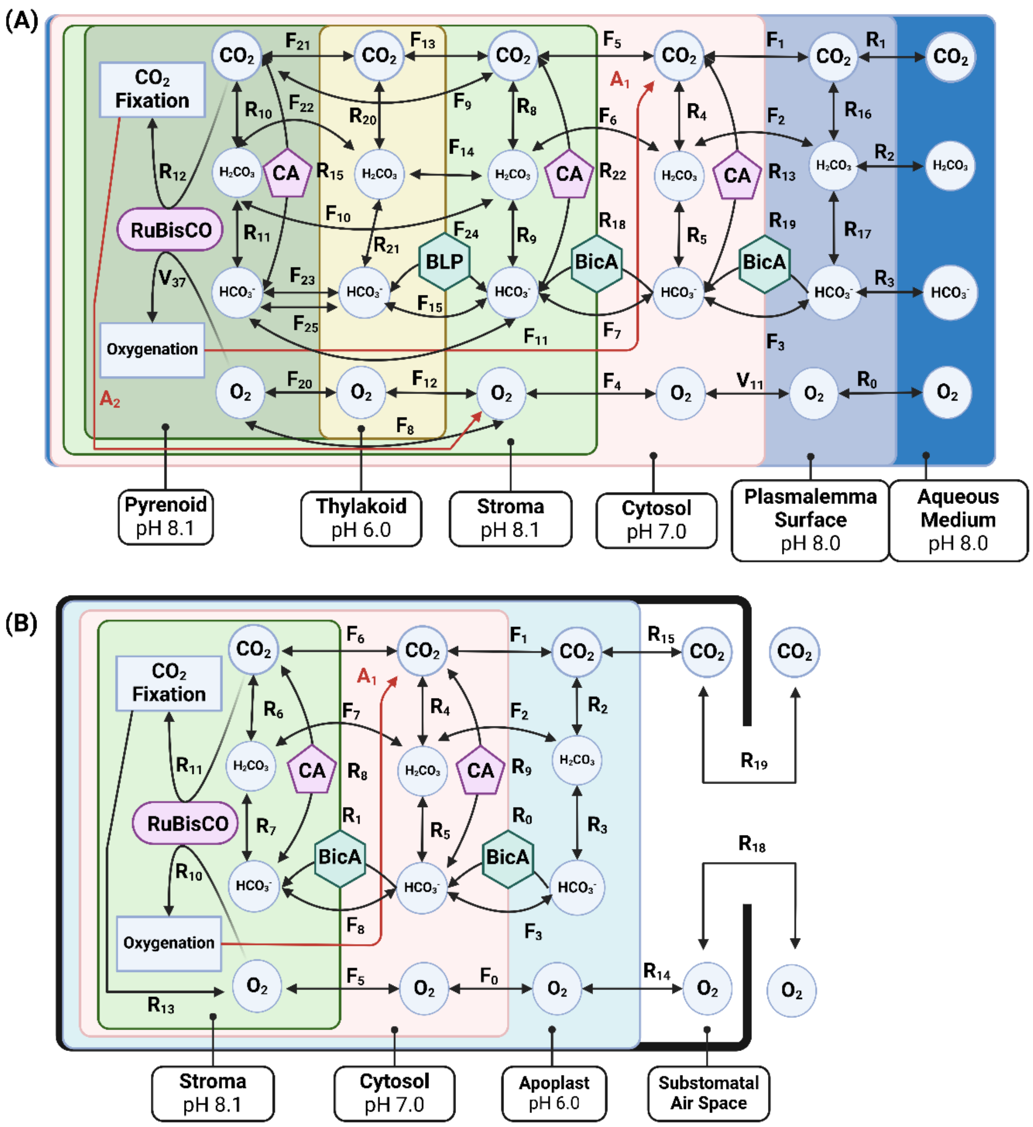

Supplementary Figure S1: Diagrammatic representations of additional models presented in this study. **(A)** a model of photosynthetic carbon assimilation in an algal containing a *C. reinhardtii* style PCCM **(B)** a model of a land plant cell that does not contain a pyrenoid. CA refers to carbonic anhydrase, BLP refers to bestrophin-like proteins that serve as membrane channels for passive bicarbonate transport, and BicA is a cyanobacterial active bicarbonate transporter. "F" is used to denote fluxes between compartments of the model. "R" is used to denote enzyme-catalyzed and spontaneous reactions as well as some processes (e.g. movement of gases into the substomatal space) where multiple processes have been aggregated into a single expression. "A" is used to denote processes that have been abstracted in the model for simplicity and/or numerical tractability and whose component processes are explicitly listed in the **Supplemental Model Equations** file. In the *VCell* implementation of the model, some strongly linked steps are combined for the sake of numerical computability. Exact specifications for all flux equations used can be found in the publicly shared model implementations in *VCell* (see code and data availability statement). Note that BicA is only active in modeling scenarios where its activity is explicitly explored and only ever at the plasmalemma or the chloroplast membrane, not both. Also note that for the sake of

numerical tractability, the carbonic-anhydrase catalyzed interconversion of CO_2 and HCO_3 in the thylakoid in models featuring a CCM (R15) is localized to the pyrenoid but uses the pH value of the thylakoid; in the real biological system, the carbonic-anhydrase is inside the thylakoid tubules that penetrate into the pyrenoid. Equations for all fluxes can be found in the **Supplemental Model Equations** file **(Supplemental Equations 247 – 397 for (A) and Supplemental Equations 157 – 246 for (B))** .

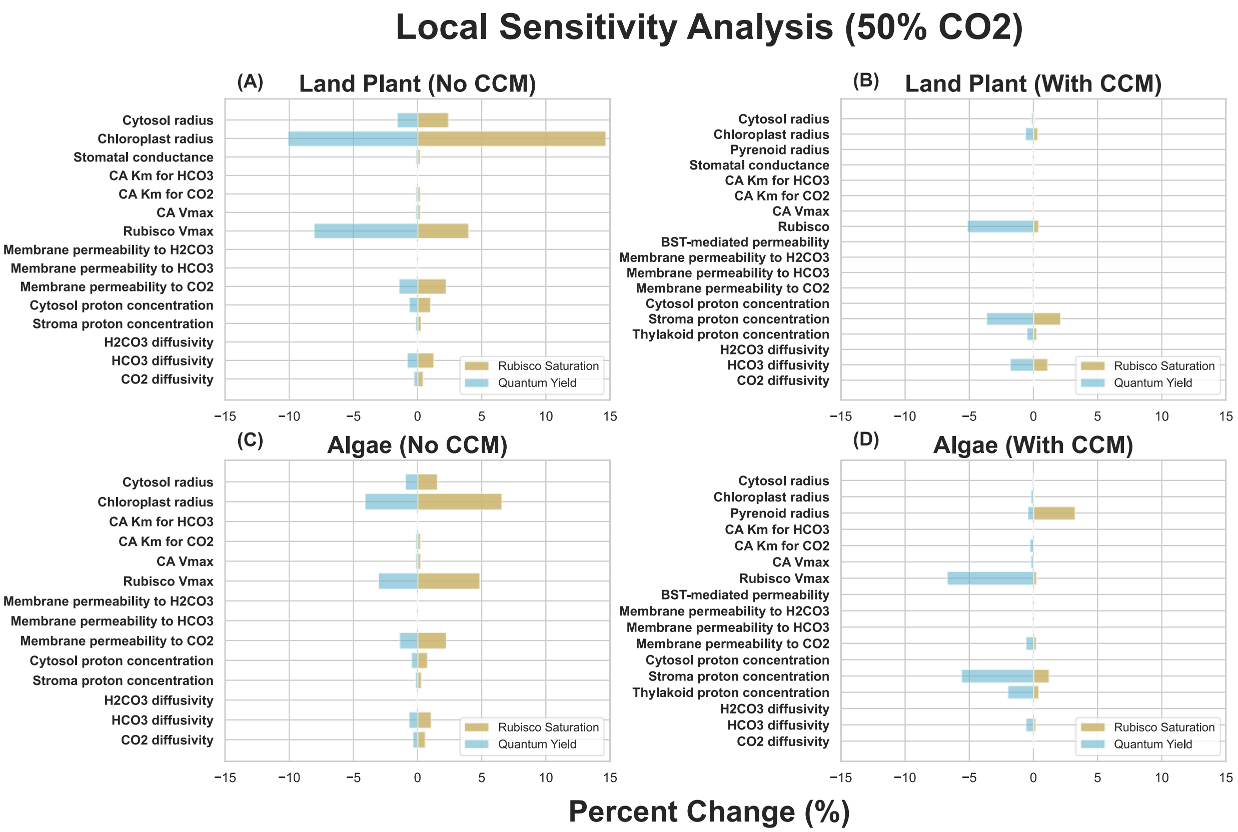

Supplementary Figure S2: Sensitivity analysis results for **(A)** the land plant model lacking a CCM at 50% external CO_2, **(B)** the algal model lacking a CCM at 50% external CO_2, **(C)** the land plant model with a CCM at 50% external CO_2, and **(D)** the algal model with a CCM at 50% external CO_2. Orange bars indicate the absolute % change of quantum yield resulting from a 10% change in the indicated parameter, and blue bars represent the same for rubisco saturation. Values are averages of the absolute % change resulting from a 10% increase and decrease; non-averaged values are shown in **Table S22**. Local sensitivities calculated at an external CO_2 concentration of 412 ppm. For both land plant models, increasing the cytosol radius by 10% resulted in problems with solving the systems numerically, so the cytosol radius was increased by 1% instead and, assuming a linear relationship between the size of radius increase and the change in rubisco saturation and quantum yield, multiplied by 10 to get the values shown in **(A-B)**.

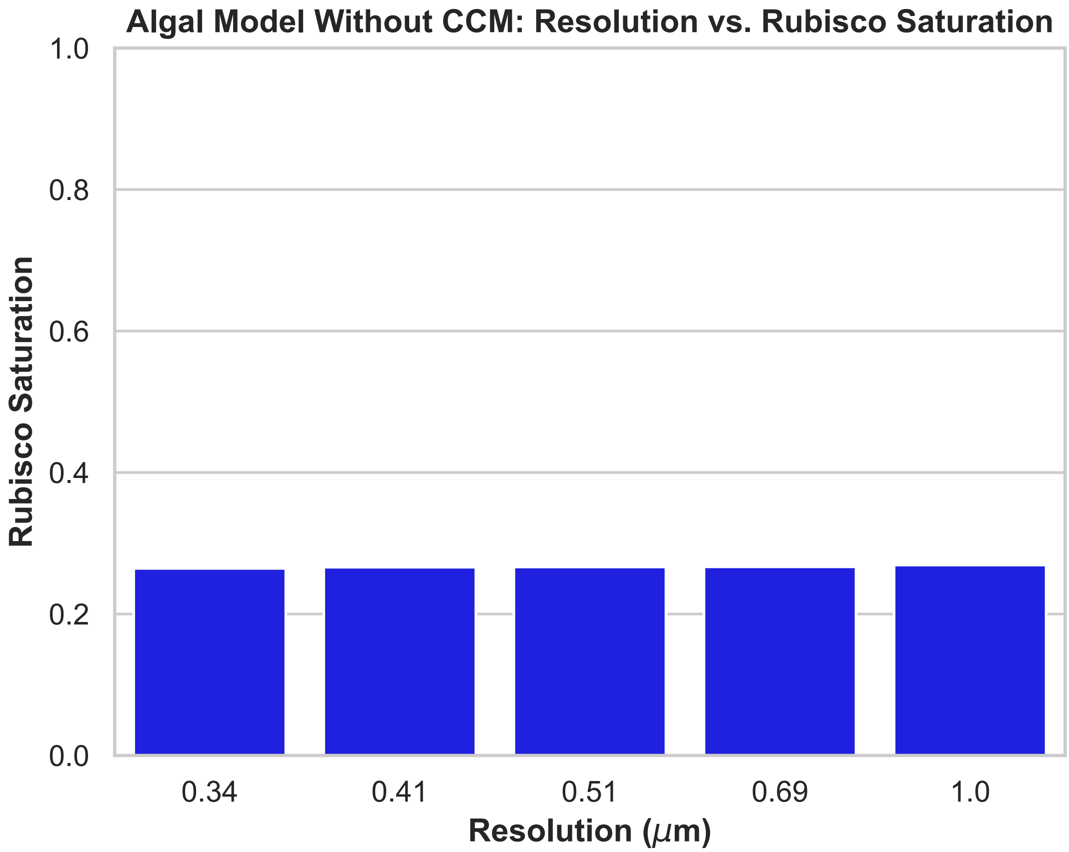

Supplementary Figure S3: Effect of simulation spatial resolution on rubisco saturation in the model of an algal cell without a CCM.

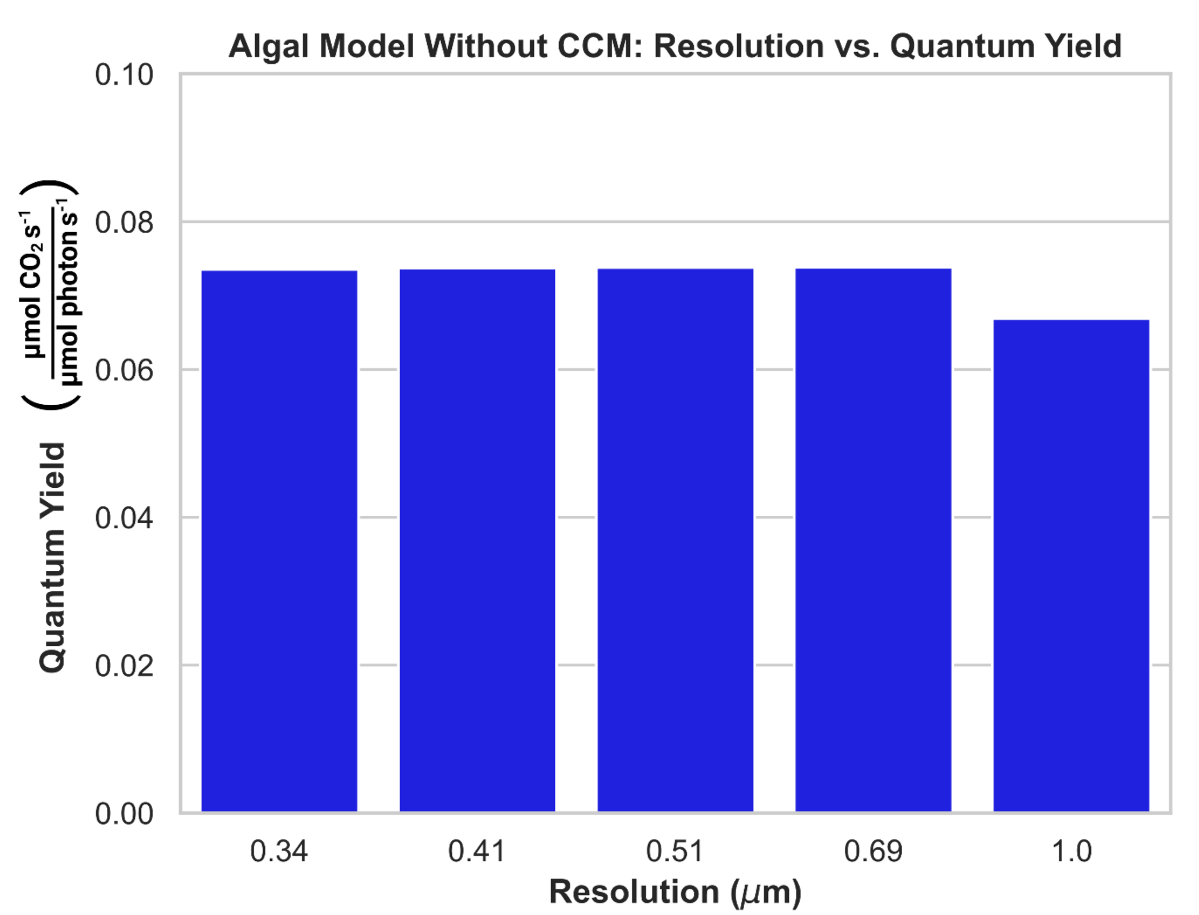

Supplementary Figure S4: Effect of simulation spatial resolution on quantum yield in the model of an algal cell without a CCM.

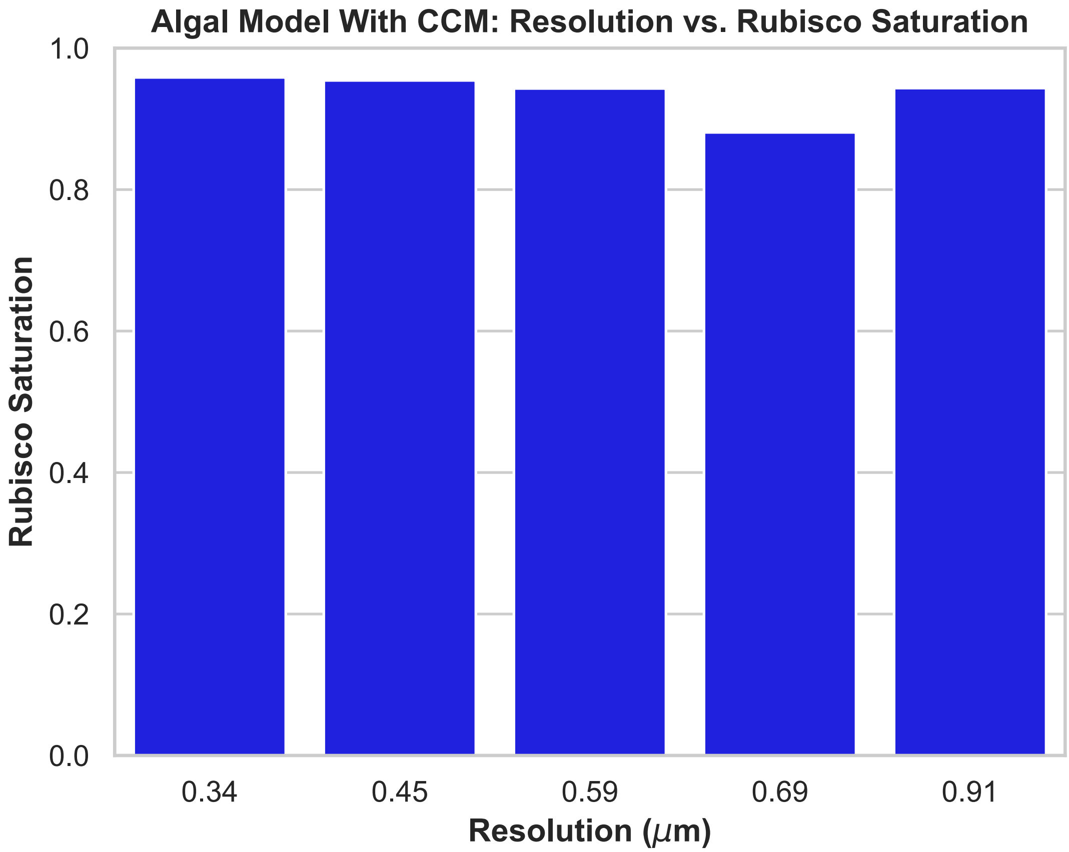

Supplementary Figure S5: Effect of simulation spatial resolution on rubisco saturation in the model of an algal cell with a CCM.

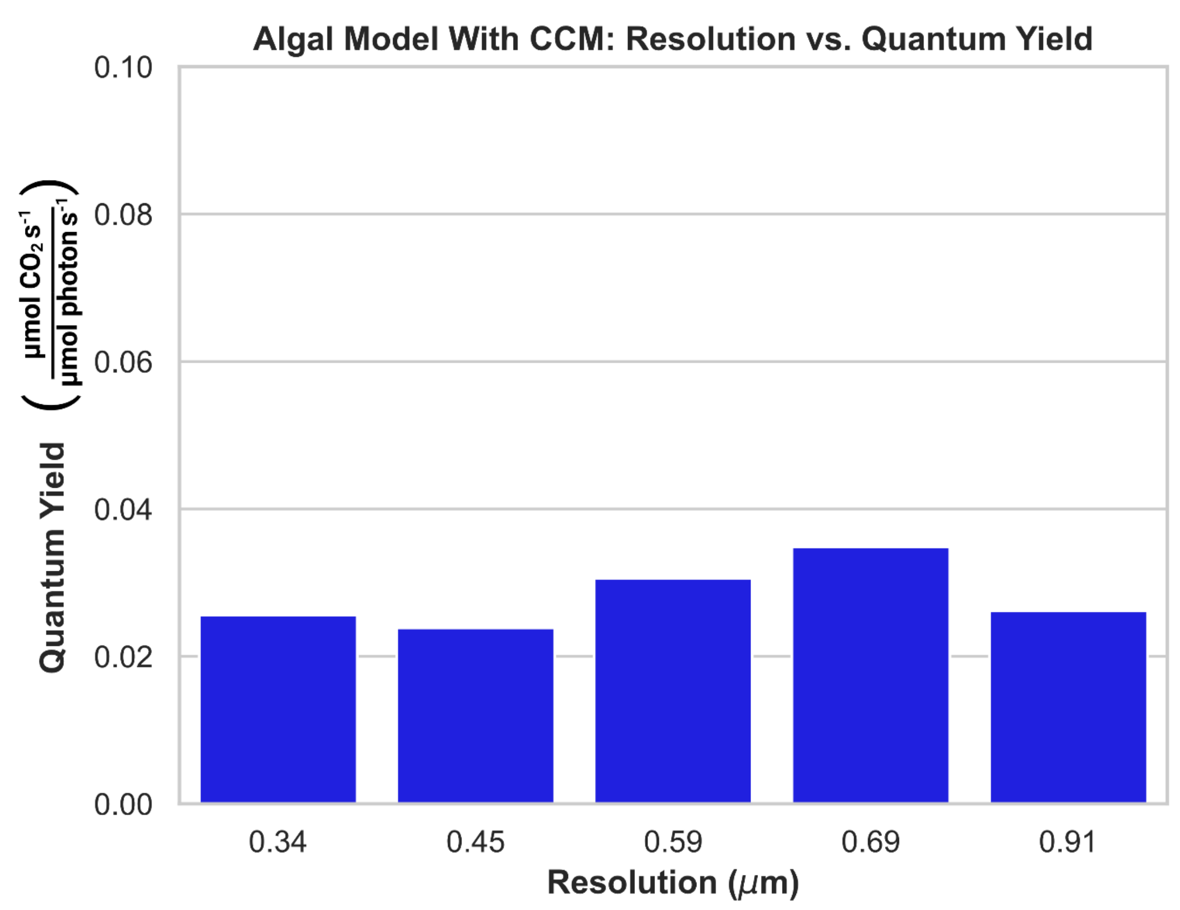

Supplementary Figure S6: Effect of simulation spatial resolution on quantum yield in the model of an algal cell with a CCM.

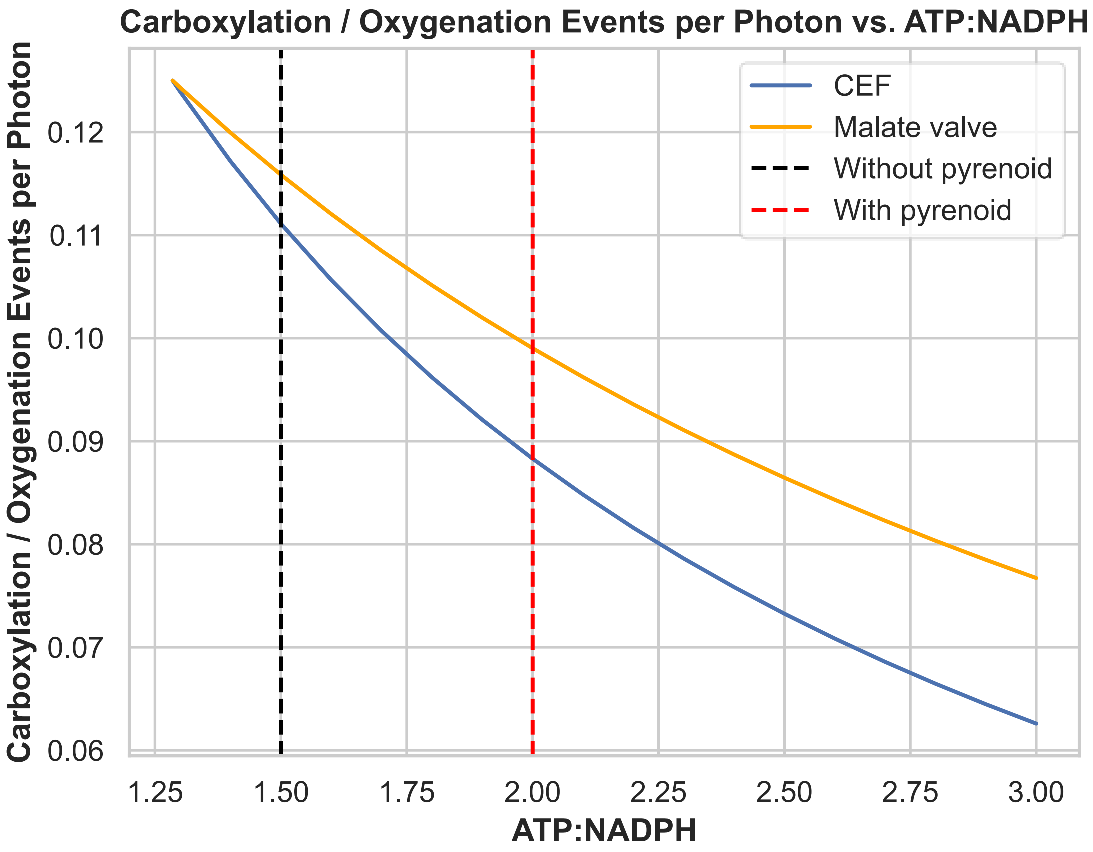

Supplementary Figure S7: Carboxylation / oxygenation events per photon as a function of varying ATP:NADPH ratios. Costs associated with using either Cyclic Electron Flow or the malate valve for increasing ATP:NADPH ratio from the products of the light reactions are taken from (Walker et al., 2020).

Supplemental References

1. B. J. Walker, D. M. Kramer, N. Fisher, X. Fu, Flexibility in the Energy Balancing Network of Photosynthesis Enables Safe Operation under Changing Environmental Conditions. *Plants (Basel, Switzerland)* **9** (2020).
