# Supplementary material for: Reaction-diffusion modeling provides insights into biophysical carbon-concentrating mechanisms in land plants
Source: Plant Physiol. 2024 Jun 10;196(2):1374–90. doi: 10.1093/plphys/kiae324 (PMC11444298; doi:10.1093/plphys/kiae324)
Supplement: kiae324_Supplementary_Data [file pp.00046.2024-s31.pdf › pp.00046.2024-s31.pdf]

Plant Physiology®

Biophysical carbon concentrating mechanisms in land plants: insights from reaction-diffusion modeling

Joshua Kaste, Berkley Walker, and Yair Shachar-Hill

Review Timeline:

Submission Date:	11-Jan-2024
Editorial Decision:	15-Feb-2024
Revision Received:	16-Apr-2024
Editorial Decision:	29-Apr-2024
Revision Received:	10-May-2024
Accepted:	11-May-2024

15-Feb-2024

Prof. Yair Shachar-Hill
Michigan State University
Plant Biology
East Lansing, Michigan 48824-1312

RE: Biophysical carbon concentrating mechanisms in land plants: insights from reaction-diffusion modeling

Dear Dr. Shachar-Hill:

We have received reviews of your manuscript, which you may view at the end of this letter. On the basis of the advice received and my own evaluation, I believe that, pending revision, this article may ultimately be acceptable for publication in Plant Physiology.

You will see below that I got two highly positive reviewer comments on your manuscript. In any case, based on the comments of the reviewers, and my reading, you will see that both reviewers have also raised several excellent points that will require your attention and a bit of work before formal acceptance. Although most of the changes required are textual ones you will see that there are significant clarifications of several issues, including

a) calibration and validation of the presented model;
b) the significance of your main results and the main conclusion, that should be added to your manuscript to provide further support to your own data;
c) several minor comments kindly added on the pdf attached file by reviewer #1 and also raised by reviewer #2.

I will expect you to bring the manuscript up to the Plant Physiology standard considering you can effectively answer the comments raised and provide an explanation for all queries raised.

Please pay careful attention to the excellent and well-considered points and comments of the reviewers when revising your manuscript which also appears to benefit from extra language attention.

When you are ready to submit your revision, click the below link and find the red arrow under your Author Tasks heading for this revision submission.

If you want to visit this version of the manuscript, you may click the link below and then click on the folder that reads "Post Decision Manuscripts".

Link Not Available

When uploading the revised version of this article, please be sure to include as complete an answer as possible to the referees' criticisms during the first round of reviews in the "response to reviewers" section.

Plant Physiology considers a revision for up to 60 days after a manuscript has been provisionally accepted; limited extension of this timeframe is at the discretion of the Monitoring Editor. A reminder notice will be sent to you 2 weeks before the deadline, at which time you may choose to contact the editorial office to request an extension; if so, please provide a justification for your request. After the expiration of this deadline, the article would need to be submitted as a resubmission and would be handled as such.

Thank you very much for giving us an opportunity to review this work. I look forward to receiving the next version.

Sincerely,

Editor, Plant Physiology

Want to add this revision deadline to your calendar? Click below!

-- Reviewer comments:
Reviewer #1 (Comments for the Author):

In this manuscript, the authors develop a spatially resolved reaction-diffusion model for the pyrenoid based CO2-concentrating mechanisms (PCCMs), which goes beyond previous work to include the whole cell and the surrounding environments.

The paper uses existing evidence and measurements to constrain the model parameters, and use the model to probe what may be important for PCCM function. Specifically, the authors highlight the importance of the lipid membrane permeability to CO2, which strongly influence the model outcomes, and the interplay between bicarbonate transport and CO2 leakage. Based on the model, the authors also speculate that the difference in cell-wall permeability may explain why C3 plants generally lack PCCMs while hornworts have evolved PCCM, which is quite interesting.

My major comment is that the authors need to clarify different aspects of the model, which will be detailed below.

1. The authors mentioned in line 122 that the thylakoid tubules are modeled as cylinders extending into the pyrenoid, so I assume that there's still exchange of inorganic carbon and oxygen at the stroma-pyrenoid interface, but there're no such arrows in Fig. 1A. Also, it might be beneficial add a column of mathematical symbols in table 1 to connect the parameter values to the different processes illustrated in Fig. 1.

2. Since the model behaviors will depend on external CO2 levels, the authors should always specify the value of external CO2 concentration when possible (e.g., in Fig. 3, 5, and 7). This is especially important in Fig. 3 since small parameter sensitivity of net CO2 assimilation (or Rubisco saturation) is trivial if the external CO2 is set to be extremely high and the Rubisco is already highly saturated with CO2.

3. The authors also need to clarify how parameters are varied in the model. For example, when the cytosol (or chloroplast) radius parameters are varied, the authors need to clarify whether the volume/area densities of the enzymes/transporters are fixed (and the total numbers are varied), or the total numbers are fixed (and densities are varied). Also, in Figures 4 and 5 where CO2 permeability and bicA Vmax are varied, please clarify whether these variations apply to all the relevant surfaces.

4. In Fig. 6, the authors did cost comparison between models with and without CCM in land plants and algae. However, only two schematic models, i.e., a land plant cell with CCM and an algal cell without CCM are shown in Fig. 1. I would suggest showing the four models altogether in Fig. 1 or at least clarify in the text how the other two scenarios are modeled. Otherwise, it's unclear (1) whether the modeled algal cell with CCM have the exact same architectures as the plant cell with CCM and (2) whether bicA transporters are also functional. The latter question is especially important since bicarbonate transport into the chloroplast is presumably minimal in C. reinhardtii under atmospheric CO2.

5. Overall, to enhance clarity, I think it would be helpful to explicitly list the set of PDEs and boundary conditions the authors used for simulation.

6. In Equation E12, the authors considered the energy cost of lumen CA activity. However, there are other CA reactions that affect the proton gradient across inter-compartment interfaces. Why do the authors ignore those other fluxes? In other words, the fluxes supported by the reaction-diffusion model may not support the assigned constant pH values. I think the authors need to perform a more rigorous analysis of proton generation and depletion in various compartments. In addition, how do the authors account for the energy cost associated with the bicA pumps?

7. The numerical simulations could benefit from improved precision. What is the spatial resolution of the simulations used to generate main figures? In principle, the simulations should be done in the regime where the spatial resolution is small enough and further decreasing the voxel size will not change the numerical solutions or the computed quantities. Based on Fig. S4 and S5 (their titles might be mislabeled), I strongly suggest that the authors should perform simulations at resolutions smaller than 0.45 um. The authors mentioned (e.g., line 117 and line 256) that certain numerical choices/modifications were made for numerical tractability to tackle the "large" number of parameters present in the model. I don't think these justifications are valid given the capabilities of modern-day computational power.

I think all the above comments need to be addressed more carefully in the text. I also have some minor comments/suggestions which can be found in the attached annotated pdf.

Reviewer #2 (Comments for the Author):

Summary of Article contributions:
The authors have developed a model for a pyrenoid-style carbon concentrating mechanism in algae and bacteria. While previous models focused on only the chloroplast this model captures transport of inorganic carbon species in the extra-cellular environment, into the cells, and through the cytosol. Such a model allows the author to interrogate how a pyrenoidal CCM interacts with cellular and external Ci conditions. In particular, they discuss how differences in Ci uptake rates and CO2 vs HCO3- availability may impact how effective a pyrenoidal CCM is in land plants growing in air vs in algae growing in water. They conjecture that these differences may help explain the ubiquity of pCCMs in algae across the evolutionary tree compared to the uniqueness of a pCCM in only hornwart land plants.
Summary of Reviewer impression:
The authors tackle the important and understudied problem of how a CCM works within the cytosol of algae and plants. This is timely, since existing models have developed a framework for modeling PCCMS from the "chloroplast inwards." So this work extends the analysis to how the existing models interact with the cellular and extra cellular environment. Overall, there are several interesting conclusions about the relationship between transport rates, energy costs, and futile cycling which are well framed for a biological audience interested in the evolutionary and plant engineering implications. I think this work could be well-placed in Plant Physiology and would be of interest to the readership. Before it can be published, I request that the authors clarify validation of the model and some of the sensitivity results. I have labeled these as major results because they may require further calculations or comparison with existing literature.
Major Revisions:
1. My main concern about the results is whether the model is sufficiently calibrated and validated to be representative. Several parameter values, including structural values are given assumed values. The authors do a good job of laying all of this out in a table and they clearly state in the text when certain assumptions are made about the geometry and model simplifications. I very much appreciate the clarity with which these assumptions are laid out. However, the authors also have a number of potentially unexpected results and it is not clear from the current manuscript if they unexpected results are due to errors introduced by the simplifying assumptions. Here are some suggestions to clarify without needing to make the model more complex.
a. I would like to see more context (semi-quantitative validation) for the model output in the results section. In the discussion the authors note that the RuBisCO saturation rates are low for some cases. What is the measured/expected saturation rate? What would be measured/expected quantum yields? It would be useful to highlight these regions/thresholds on the result plots. Do the baseline parameters of the model produce reasonable flux and cost values besides the Net CO2 assimilation rates?
b. Connect the sensitivity analysis performed in Fig 3 with the values in Table 1 and the assumptions made to simplify the geometry. The sensitivity analysis in Fig 3 does not appear to be done in terms of the parameters in Table 1-perhaps because these parameters combine to make effective parameters that are varied in the model.
c. For what quantity of interest (output of the model) is the sensitivity analysis calculated? It may be that quantum efficiency is sensitive to different parameters than rubisco saturation, but only one sensitivity is reported. I also was unclear if only changes to an increase in each parameter are studied or if the parameters were also decreased by 10%.
d. I am slightly concerned about the assumption that the carbonic anhydrase conversion between HCO3- and CO2 which happens in the tubules is assumed to be in the pyrenoid. The separation of the Ci pools in the pyrenoid vs tubules was one of the major components of the Fei et al, 2023 model. As can be seen in Fig 1c of their paper, more CO2 is trapped locally in the thylakoids than is in the pyrenoid matrix/stroma. It may be that by not having a separate compartment for the tubules, the CO2 is escaping at a higher rate. I am not sure how to test this with the given model but it may explain some of the current model's strong sensitivity to CO2 permeability.

2. Clarify the equations describing transport between modeling compartments and diffusion within compartments-these equations should be provided for completeness in the main paper. Is standard Fickian diffusion used for transport between compartments? I realize this is in the supplement, but more detail about the rest of the model form besides the reactions should be included. I think you only need to show the transport eqn once, not for each compartment.
a. Permeability estimates-I suggest the authors look at a previous modeling paper in cyanobacteria CCMs (Mangan, Flamholz, Hood, Milo, Savage PNAS 2016 https://www.pnas.org/doi/full/10.1073/pnas.1525145113). Can the authors clarify if the permeability and modeling are consistent with the pH dependent permeabilities derived in the supplement? I think it is, since the authors are modeling CO2, HCO3-, and H2CO3 independently and so the reductions used in the 2016 paper are not needed? Is the pH dependence the same? I understand that this previous work used a CO2 permeability assuming only the membrane without channels, and one of the interesting results is about potentially higher CO2 permeabilities.
Minor comments:
Equation 4 and 9 - check the consistency of HCO3- and HCO3 (without the -)
Figure 2 - would be useful to present the experimental ranges in the table 2 as bars on the plot (if the journal allows values in both tables and plots). If only one is allowed keep the table.
Figure 5 - missing a caption for subplot G
Figure 6 - both x axis are in uM but text mentions ppm for land plants -- would be clearer to relate to plots if both values of uM and ppm were given for land plants. (or if relevant ppm values were translated to uM and shown on the plot.
Figure 7 (and generally) - consistency between PCCM and CCM it is PCCM in figure caption but CCM on plot

Editor comments:

You will see below that I got two highly positive reviewer comments on your manuscript. In any case, based on the comments of the reviewers, and my reading, you will see that both reviewers have also raised several excellent points that will require your attention and a bit of work before formal acceptance. Although most of the changes required are textual ones you will see that there are significant clarifications of several issues, including

a) calibration and validation of the presented model;
b) the significance of your main results and the main conclusion, that should be added to your manuscript to provide further support to your own data;
c) several minor comments kindly added on the pdf attached file by reviewer #1 and also raised by reviewer #2.

I will expect you to bring the manuscript up to the Plant Physiology standard considering you can effectively answer the comments raised and provide an explanation for all queries raised.

Please pay careful attention to the excellent and well-considered points and comments of the reviewers when revising your manuscript which also appears to benefit from extra language attention.

Author response:

We would like to thank you and the reviewers for your careful consideration of our manuscript and the insightful feedback provided. We have provided detailed responses to every comment and suggestion below and have made extensive revisions and additions to the main text and supplemental materials, including additional analyses and context. Below, we have briefly summarized our responses to the main points raised by the reviewers:

1. Both reviewers 1 and 2 requested more explicit descriptions of the model architecture, parameterization, and sensitivity analyses. To address these points, we have added a detailed supplemental document (Supplemental Model Equations) containing the calculations for all partial differential equations, derived geometric parameters, boundary conditions, and intra- and inter-compartmental fluxes in the model. Along with this, we have provided more detailed descriptions of some aspects of the modeling framework we are using in the manuscript itself.
2. Reviewer 2's main concern was that additional validation evidence should be provided in support of the model's prediction accuracy. We have added additional checks to the main text describing how the model's quantum yield and rubisco saturation predictions line up nicely with empirical measurements from the literature.
3. Reviewer 1 was concerned about whether the spatial resolution used for this study was sufficient. We have expanded on our previous, relatively brief treatment of the sensitivity of our model results to spatial resolution by comparing it with the state-of-the-art in this area of modeling, which is the Fei et al. 2022 study in Nature Plants. We show that the local sensitivity of our reported rubisco saturation and quantum yield values is comparable to that achieved by the Fei et al. 2022 study and small in comparison to the model's sensitivity to a number of uncertain parameters, as shown in our general sensitivity analysis results.

Additionally, during the course of going through and generating the Supplemental Model Equations file, we noticed small inconsistencies between our parameter table and some of the simulations we ran, which prompted us to rerun and reanalyze the affected simulations. This resulted in small changes in steady-state fluxes and concentrations in some of the models, but did not affect any of the conclusions of the study. Figures 2-7 and text referencing these figures have all been updated to reflect the slight changes.

We look forward to receiving a response on this updated and improved revision of our manuscript and to publishing this work with *Plant Physiology*.

--- Reviewer comments:

Reviewer #1 (Comments for the Author):

[…] My major comment is that the authors need to clarify different aspects of the model, which will be detailed below.

1. The authors mentioned in line 122 that the thylakoid tubules are modeled as cylinders extending into the pyrenoid, so I assume that there's still exchange of inorganic carbon and oxygen at the stroma-pyrenoid interface, but there're no such arrows in Fig. 1A. Also, it might be beneficial add a column of mathematical symbols in table 1 to connect the parameter values to the different processes illustrated in Fig. 1.

Author Response: First, we would like to thank the reviewer for their thoughtful comments.

1. It is correct that in the current model, inorganic carbon and oxygen do exchange at the stroma-pyrenoid interface as well as through the thylakoids. Arrows representing these processes have been added in **Fig. 1A**.
2. We have added an additional supplemental file (**Supplemental Model Equations**) containing the equations describing every PDE, boundary condition, and flux in the different models analyzed in this study, thus linking the parameter values in **Table 1** to each process shown in **Figure 1**. We have used the same nomenclature in these equations as we do in the parameter table, which we believe helps connect these values with the actual calculations done in this study.

2. Since the model behaviors will depend on external CO2 levels, the authors should always specify the value of external CO2 concentration when possible (e.g., in Fig. 3, 5, and 7). This is especially important in Fig. 3 since small parameter sensitivity of net CO2 assimilation (or Rubisco saturation) is trivial if the external CO2 is set to be extremely high and the Rubisco is already highly saturated with CO2.

Author Response: We have added the external CO_2 concentration used in the captions for **Fig. 3, 5**, and **7**. We also agree that it is useful to consider the local sensitivity results presented in Figure 3 in the context of the CO2 concentration used, so we have added an additional figure **(Figure S3)** that shows the sensitivity at 50% external CO2, rather than 100%.

3. The authors also need to clarify how parameters are varied in the model. For example, when the

cytosol (or chloroplast) radius parameters are varied, the authors need to clarify whether the volume/area densities of the enzymes/transporters are fixed (and the total numbers are varied), or the total numbers are fixed (and densities are varied). Also, in Figures 4 and 5 where CO2 permeability and bicA Vmax are varied, please clarify whether these variations apply to all the relevant surfaces.

Author Response:

1. We have added clarifications to the text **(lines 278-283)** on exactly how geometric parameters are being varied and how these interact with related enzymes/transporters. Our approach when varying radii was to keep the densities of enzymes/transporters fixed. When CO_2 permeability is varied, this applies to all lipid membranes in the model.
2. We have clarified how BicA Vmax is varied in the captions for **Figures 4 and 5**. All exploration of BicA Vmax are done in the context of models where only one surface has an active pump (either the chloroplast surface or plasmalemma surface).

4. In Fig. 6, the authors did cost comparison between models with and without CCM in land plants and algae. However, only two schematic models, i.e., a land plant cell with CCM and an algal cell without CCM are shown in Fig. 1. I would suggest showing the four models altogether in Fig. 1 or at least clarify in the text how the other two scenarios are modeled. Otherwise, it's unclear (1) whether the modeled algal cell with CCM have the exact same architectures as the plant cell with CCM and (2) whether bicA transporters are also functional. The latter question is especially important since bicarbonate transport into the chloroplast is presumably minimal in C. reinhardtii under atmospheric CO2.

Author Response:

1. We have added additional supplemental model diagrams as suggested **(Fig S1)** and now refer to them in the caption of **Fig 1**, which should increase clarity here. We put these figures in the supplement rather than adding them to Figure 1 due to space constraints.
2. We have added additional text to the **Fig 1** caption to clarify that the BicA pumps in our simulations are only “turned on” – i.e., are allowed to carry flux – in the chloroplast membrane pump and plasmalemma membrane pump simulations and are not allowed to do so in any of the other simulations.

5. Overall, to enhance clarity, I think it would be helpful to explicitly list the set of PDEs and boundary conditions the authors used for simulation.

Author Response: We have added a supplemental file **(Supplemental Model Equations)** that lists out all PDEs and boundary conditions for the Virtual Cell models used in this study.

6. In Equation E12, the authors considered the energy cost of lumen CA activity. However, there are other CA reactions that affect the proton gradient across inter-compartment interfaces. Why do the authors ignore those other fluxes? In other words, the fluxes supported by the reaction-diffusion model may not support the assigned constant pH values. I think the authors need to perform a more rigorous analysis of proton generation and depletion in various compartments. In addition, how do the authors account for the energy cost associated with the bicA pumps?

Author Response:

1. We have clarified our reason for ignoring the costs associated with pH balancing in the stroma and cytosol to the manuscript **(lines 262-273)**. As we now explain in the text, our assumed energy costs associated with lumen CA activity assume that the protons depleted by CA activity are compensated for by light-driven pumping of protons into the lumen from the stroma. Taking our algal model with a PCCM at ambient CO2 levels as an example, after accounting for the flux of protons from the stroma into the lumen implied by our energy cost calculations, the model predicts an extremely small inward flux of protons would be necessary to maintain the pH of the stroma. This flux could be achieved for free using proton channels between the stroma and cytosol, and balancing of the cytosolic pH could in turn be achieved either by active pumping of protons from the external environment in the case of an algae living in marine conditions, or by passive transport of protons from the exterior in land plants or algae living in media below a pH of 7. If we assume a similar stoichiometry of pumped protons to ATP for active transport across the plasmalemma, the cost associated with this pumping activity would amount to ~0.07% of the total costs associated with running the PCCM. Given the extremely small contribution of this component of the system to the energetic costs and the assumptions + alternative possibilities that would need to be explored to understand it, we elected to leave this out of our energy calculations.
2. For the energy cost associated with BicA pumps, we followed previous studies in assuming a value of 0.25 ATP per pumped H^+ (i.e. 4 H^+ per ATP) (1).

7. The numerical simulations could benefit from improved precision. What is the spatial resolution of the simulations used to generate main figures? In principle, the simulations should be done in the regime where the spatial resolution is small enough and further decreasing the voxel size will not change the numerical solutions or the computed quantities. Based on Fig. S4 and S5 (their titles might be mislabeled), I strongly suggest that the authors should perform simulations at resolutions smaller than 0.45 um. The authors mentioned (e.g., line 117 and line 256) that certain numerical choices/modifications were made for numerical tractability to tackle the "large" number of parameters present in the model. I don't think these justifications are valid given the capabilities of modern-day computational power.

Author Response: We appreciate this comment and acknowledge the reviewer's concerns, but believe that the sensitivity of our model compares well with the current state-of-the-art in this area and have added some analysis and discussion of this to the manuscript **(lines 346-350)**.

In Fei et al. 2022, Supplemental Figure 31, the authors of that study present their spatial sensitivity results and use them to justify a mesh size of 5000. This "mesh size" does not have a direct analog in our study, since Fei et al. 2022, by assuming perfect radial symmetry, numerically treat the system as one-dimensional. However, from a computational point of view, each additional unit of mesh size essentially corresponds to an additional compartment for which relevant quantities must be computed, so we will treat them as analogous to the voxels in our 3D model. Fei et al. report small changes in normalized CO_2 fixation at a mesh size of 5000 and use this to justify their choice of this value and not a higher one. The value they compute to demonstrate this is % relative change between a mesh size N and mesh size

N+(0.1*N); that is, between a given mesh size and one 10% larger or smaller. We presented a coarser, but similarly broad exploration in **Figs. S4 and S5.**

Focusing on rubisco saturation, since this maps to the normalized CO_2 fixation used in Fei et al. 2022, our results show comparable % relative change. Going from a spatial resolution of 0.91um to 0.61um results in a ~91% increase in mesh size (9,261 voxels to 27,000 voxels). Normalizing the % change in rubisco saturation to a 10% change in voxel number gives a relative % change value of 0.10%, which appears similar to the values reported in Fei et al 2022. Although they did not report sensitivity of any measures of energy efficiency, the relative % change value of quantum yield is 0.12%.

We believe this demonstrates that this study is achieving comparable relative stability between our predictions and the current state-of-the-art, and doing so in a more expansive, and as a consequence, substantially more computationally expensive system. The effect of spatial resolution is very small relative to many of the parameters whose sensitivity is explored in **Fig 3** and far too small to affect any of the trends reported on and explored in this manuscript. Together with the fact that, given our current resources the compute time alone that it would require to rerun the simulations presented in this study below a resolution of 0.45 um would be in excess of the two months we have been allotted to revise the study, we believe the current spatial resolution of the models is sufficient.

I think all the above comments need to be addressed more carefully in the text. I also have some minor comments/suggestions which can be found in the attached annotated pdf.

For the minor comments and suggestions in the annotated PDF:

“Why is pH chosen to be 8.0?”

Author response: We chose a pH of 8.0 to represent a marine environment (pH values in the ocean vary from approximately 8 – 8.25 globally). We have added a more recent reference to Table 1 for this value that reports this range.

“Typo” (in Eq. 9)

Author response: The typo in equation 9 has been corrected.

“Typo? SI figure says ‘0.34 um’”

Author response: Yes, this was a typo. The text has been corrected to read 0.34 µm.

“The caption for panel G is missing.”

Author response: The missing caption has been added.

“Please convert these numbers to the same units for better comparison.”

Author response: The numbers have been converted to the same units to facilitate better comparison.

“I would suggest adding arrows to the plot to indicate which “effective porosity” values correspond to typical C3 plants and hornworts”

Author response: There are not precise measurements of the effective porosity of cell walls for either C3 plants or hornworts, though, as we discuss in the main text, values in the 10^{-1} range have been inferred for C3 plants, and in this study we explore the effects of much lower values for hornworts motivated by the extremely low stomatal conductances that have been reported and qualitative characterizations of hornwort cell wall properties. We think arrows pointing to specific values in this figure may mislead the reader, so we have kept it as is.

Reviewer #2 (Comments for the Author):

Summary of Article contributions:
[…] Before it can be published, I request that the authors clarify validation of the model and some of the sensitivity results. I have labeled these as major results because they may require further calculations or comparison with existing literature.
Major Revisions:
1. My main concern about the results is whether the model is sufficiently calibrated and validated to be representative. Several parameter values, including structural values are given assumed values. The authors do a good job of laying all of this out in a table and they clearly state in the text when certain assumptions are made about the geometry and model simplifications. I very much appreciate the clarity with which these assumptions are laid out. However, the authors also have a number of potentially unexpected results and it is not clear from the current manuscript if they unexpected results are due to errors introduced by the simplifying assumptions. Here are some suggestions to clarify without needing to make the model more complex.
a. I would like to see more context (semi-quantitative validation) for the model output in the results section. In the discussion the authors note that the RuBisCO saturation rates are low for some cases. What is the measured/expected saturation rate? What would be measured/expected quantum yields? It would be useful to highlight these regions/thresholds on the result plots. Do the baseline parameters of the model produce reasonable flux and cost values besides the Net CO2 assimilation rates?

Author Response: We would like to thank the reviewer for their thoughtful comments. To address this request for measured/expected quantum yields and saturation rates, we have added a comparison between our model estimates and literature values in the Results section **(Lines 305-321)**.

To summarize, for land plants – both C3 and C4 – quantum yields of photosynthesis around 0.05 have been reported, which lines up with our model estimates for the land plant case. Similar values have been reported for C4 plants. To assess whether the “land plant with a CCM” model lines up with C4 plants, which use a biochemical and not a biophysical CCM, we compared the predicted quantum yield of a “with CCM” model at an external CO_2 concentration that results in a substomatal CO_2 level similar to that reported in maize. This quantum yield is 0.055, once again matching closely with expectations. For rubisco saturation, we can estimate from A/Ci curve measurements (e.g., from(2)) values around ~0.25, which are very similar to the saturation values predicted by our land plant model without a CCM under atmospheric CO2 conditions (0.2518 at a lipid membrane permeability 1X the Gutknecht value).

b. Connect the sensitivity analysis performed in Fig 3 with the values in Table 1 and the assumptions made to simplify the geometry. The sensitivity analysis in Fig 3 does not appear to be done in terms of

the parameters in Table 1-perhaps because these parameters combine to make effective parameters that are varied in the model.

Author Response: To clarify how the parameters in Table 1 relate to the actual equations used in the models, we have added a Supplemental Model Equations file detailing every PDE, boundary conditions, and flux used in the land plant and algal models with and without PCCMs incorporated. We have also added the symbols used to refer to the parameters in these equations to **Table 1**. These equations also show how the geometric simplifications that were made in the model were implemented.

c. For what quantity of interest (output of the model) is the sensitivity analysis calculated? It may be that quantum efficiency is sensitive to different parameters than rubisco saturation, but only one sensitivity is reported. I also was unclear if only changes to an increase in each parameter are studied or if the parameters were also decreased by 10%.

Author Response: We have added text to the caption that clarifies how sensitivity is calculated **(lines 275-283)**. The sensitivity analysis results presented in **Fig 3** are calculated with respect to both rubisco saturation and quantum yield. In short, we calculated and then plotted out the average of the magnitude of the change resulting from a 10% increase and a 10% decrease.

d. I am slightly concerned about the assumption that the carbonic anhydrase conversion between HCO3- and CO2 which happens in the tubules is assumed to be in the pyrenoid. The separation of the Ci pools in the pyrenoid vs tubules was one of the major components of the Fei et al, 2023 model. As can be seen in Fig 1c of their paper, more CO2 is trapped locally in the thylakoids than is in the pyrenoid matrix/stroma. It may be that by not having a separate compartment for the tubules, the CO2 is escaping at a higher rate. I am not sure how to test this with the given model but it may explain some of the current model's strong sensitivity to CO2 permeability.

Author Response: The explanation of this simplification in the text may have been misleading, so we have corrected the text to make this point clearer **(see Figure 1 caption)**.

Our model does actually separately model the tubule and pyrenoid Ci pools. Because of the difference in scale between this study's model and the Fei et al. 2022 model, it was necessary to have the thylakoid tubules abut the pyrenoid instead of penetrating into it. Based on localization evidence, Fei et al. 2022 assume that lumen CA is found exclusively in the section of thylakoid tubules that are within the radius of the pyrenoid's starch sheath. To replicate this behavior without explicitly modeling the intra-pyrenoid tubule spaces, we made the assumption that there is a CA in the pyrenoid that is interconverting CO2 and HCO3 as if it were exposed to the pH of the thylakoid, as opposed to the pyrenoid (which is assumed to be similar/the same as the stroma, ~pH 8).

Our results do indeed show that the assumed value of lipid membrane permeability to CO_2 has a noteworthy impact on the relative efficiency of incorporating a PCCM vs. not incorporating one, which is what we show in **Figure 6**. However, this effect is driven by changes in the efficiency with which the model without a CCM fixes carbon (for exact numbers, see **Supplemental Tables 7-10**). Given that this key result should be robust against the simplification and the fact that the validation results we present

indicate that our with-CCM models are mirroring empirical observation in a number of key parameters, we feel justified in making the simplification and in presenting the results as-is.

2. Clarify the equations describing transport between modeling compartments and diffusion within compartments-these equations should be provided for completeness in the main paper. Is standard Fickian diffusion used for transport between compartments? I realize this is in the supplement, but more detail about the rest of the model form besides the reactions should be included. I think you only need to show the transport eqn once, not for each compartment.

Author Response: Clarification on this point has been added on **line 202**. Transport between voxels on opposite sides of a membrane is calculated using equation **E8** in the text and numerically implemented in the simulation as a Neumann boundary condition **(E12)**. Diffusion within a compartment is described using standard Fickian diffusion and an equation describing this has been added to the text **(lines 192-194)**.

a. Permeability estimates-I suggest the authors look at a previous modeling paper in cyanobacteria CCMs (Mangan, Flamholz, Hood, Milo, Savage PNAS 2016 https://www.pnas.org/doi/full/10.1073/pnas.1525145113). Can the authors clarify if the permeability and modeling are consistent with the pH dependent permeabilities derived in the supplement? I think it is, since the authors are modeling CO2, HCO3-, and H2CO3 independently and so the reductions used in the 2016 paper are not needed? Is the pH dependence the same? I understand that this previous work used a CO2 permeability assuming only the membrane without channels, and one of the interesting results is about potentially higher CO2 permeabilities.

Author Response: The reviewer is correct in their observation that the derivations of the pH dependence of permeabilities to Ci in Mangan et al. 2016 (specifically pages 8-10 of the supplement) are necessitated by their lumping together of $HCO3^-$ and H_2CO_3, which are treated independently in our study. The baseline permeability values we use are the same as those in that study, but without the pH dependent correction due to our model's different handling of the Ci species.

Minor comments:
Equation 4 and 9 - check the consistency of HCO3- and HCO3 (without the -)

Author response: We have gone through and corrected HCO_3 to HCO_3^- in the equations and manuscript text.

Figure 2 - would be useful to present the experimental ranges in the table 2 as bars on the plot (if the journal allows values in both tables and plots). If only one is allowed keep the table.

Author response: Unfortunately, we were unable to find a way to incorporate the experimental ranges from **Table 2** into **Figure 2** in a way that did not significantly reduce the legibility of the figure, so we have kept the figure and table as separate visual elements.

Figure 5 - missing a caption for subplot G

Author response: We have added the caption for subplot G to **Figure 5**.

Figure 6 - both x axis are in uM but text mentions ppm for land plants -- would be clearer to relate to plots if both values of uM and ppm were given for land plants. (or if relevant ppm values were translated to uM and shown on the plot.

Author response: We have added the converted ppm values for the land plant panel in **Figure 6**.

Figure 7 (and generally) - consistency between PCCM and CCM it is PCCM in figure caption but CCM on plot

Author response: Figure 7 has been corrected and consistency has been checked throughout.

REFERENCES

1. G. D. Price, M. R. Badger, S. von Caemmerer, The Prospect of Using Cyanobacterial Bicarbonate Transporters to Improve Leaf Photosynthesis in C3 Crop Plants. *Plant Physiology* **155**, 20–26 (2010).

2. Y. Xu, *et al.*, Daylength variation affects growth, photosynthesis, leaf metabolism, partitioning, and metabolic fluxes. *Plant Physiology* kiad507 (2023). https://doi.org/10.1093/plphys/kiad507.

29-Apr-2024

Prof. Yair Shachar-Hill
Michigan State University
Plant Biology
East Lansing, Michigan 48824-1312

RE: Biophysical carbon concentrating mechanisms in land plants: insights from reaction-diffusion modeling

Dear Dr. Shachar-Hill:

We have received reviews of your manuscript, which you may view at the end of this letter. Based on the advice received and my own evaluation, I believe that pending a minor revision, this article may ultimately be acceptable for publication in Plant Physiology.

You will see that both previous reviewers have come to similar opinions about your paper.
On balance, I think that your results are sufficiently important that I would like to see this work published in Plant Physiology. However, both reviewers agree that the presentation of the manuscript remains unsatisfactory and has minor additional notes and I agree with. I will give you one final chance to bring the manuscript up to standard and will allow one more resubmission.

I will make the final decision on the resubmitted work myself without further recourse to the reviewers.

I am happy to be able to bring you encouraging news.

When you are ready to submit your revision, click the below link and find the red arrow under your Author Tasks heading for this revision submission.

If you want to visit this version of the manuscript, you may click the link below and then click on the folder that reads "Post Decision Manuscripts".

Link Not Available

When uploading the revised version of this article, please be sure to include as complete an answer as possible to the referees' criticisms during the first round of reviews in the "response to reviewers" section.

Plant Physiology considers a revision for up to 60 days after a manuscript has been provisionally accepted; limited extension of this timeframe is at the discretion of the Monitoring Editor. A reminder notice will be sent to you 2 weeks before the deadline, at which time you may choose to contact the editorial office to request an extension; if so, please provide a justification for your request. After the expiration of this deadline, the article would need to be submitted as a resubmission and would be handled as such.

Thank you very much for giving us an opportunity to review this work. I look forward to receiving the next version.

Sincerely,

Editor, Plant Physiology

Want to add this revision deadline to your calendar? Click below!

--- Reviewer comments:
Reviewer #1 (Comments for the Author):

I'm overall satisfied with the changes the authors made to address my previous comments. So, I recommend publishing the

revised manuscript at Plant Physiology.

I have a few additional notes to the authors regarding the point on numerical accuracy and spatial resolution:
- First of all, the spatial resolution of a numerical model should be chosen such that further decreasing it does not change the values of any computed quantities. The results in Fig. S7 clearly suggests that, at least in that figure, the spatial resolution is not small enough to achieve that.
- Secondly, I disagree with the authors that the numerical simulations in the current manuscript are comparable with the current state of the art. Based on personal correspondence with Fei et al., they used a mesh size of 5000 not because they could not use a finer mesh, but because that it's sufficient to yield an accurate numerical solution for their model. The state-of-the-art solver can solve a PDE problem with 1 million degrees of freedoms (I guess it's equivalent to voxels) in minutes on a single CPU. Furthermore, one can have hundreds of CPU cores on an academic HPC cluster. So I'm not convinced that computational power should be the limiting factor here.
- However, I believe the authors that further decreasing the spatial resolution would not affect any of the trends reported in the manuscript.

Reviewer #2 (Comments for the Author):

Thank you to the authors for careful consideration of the reviewer comments and increased details of the models and simulation. I have two further questions/recommendations but recommend the work for publication.
1. I think I now better understand how the thylakoid tubules are treated in the modeling for the CCM. It seems that this treatment may result in a different surface to volume ratio than is used in the Fai et al paper, which would effect transport rates of inorganic carbon between thylakoid and pyrenoid. Indeed the algal simulation with CCM is (highly?) sensitive to the radius of the thylakoid indicating that this simplification may have an effect. Could the authors clarify if the assumed tubule surface to volume ratios and in particular the surface area which will control the effective transport rate, are within the range of observed physiological values? I believe the geometry of the pyrenoid vary, so the default values assumed in Fei are not necessarily unique. Could the authors include any comments about implications of the sensitivity analysis results?
2. It is becoming more and more standard to make code available with publication of a model. Can the authors make their code or model definition files available upon publication? I encourage them to do so regardless of whether the journal requires it.

Reviewer #1 (Comments for the Author):

I'm overall satisfied with the changes the authors made to address my previous comments. So, I recommend publishing the revised manuscript at Plant Physiology.

Author response: We are pleased that the reviewer now recommends acceptance in Plant Physiology and are grateful for their work and input. We have made some comments below about their additional notes.

I have a few additional notes to the authors regarding the point on numerical accuracy and spatial resolution:

- First of all, the spatial resolution of a numerical model should be chosen such that further decreasing it does not change the values of any computed quantities. The results in Fig. S7 clearly suggests that, at least in that figure, the spatial resolution is not small enough to achieve that.

- Secondly, I disagree with the authors that the numerical simulations in the current manuscript are comparable with the current state of the art. Based on personal correspondence with Fei et al., they used a mesh size of 5000 not because they could not use a finer mesh, but because that it's sufficient to yield an accurate numerical solution for their model. The state-of-the-art solver can solve a PDE problem with 1 million degrees of freedoms (I guess it's equivalent to voxels) in minutes on a single CPU. Furthermore, one can have hundreds of CPU cores on an academic HPC cluster. So I'm not convinced that computational power should be the limiting factor here.

- However, I believe the authors that further decreasing the spatial resolution would not affect any of the trends reported in the manuscript.

Author response: We have added some additional discussion of these limitations to the Discussion section **(lines 624-635).** We agree with the reviewer that, in its current implementation, the spatial resolution of our models has not reached the point where there are no further changes in any computed quantities; however, the changes in these computed quantities are extremely small and, as the reviewer agrees, will not affect any of the reported trends. The software used in this study, *Virtual Cell*, sacrifices the speed of the approach taken by Fei et al. for a greater deal of portability and shareability with the broader community (as shown by our models being publicly viewable and useable through a user-friendly interface). Together with the fact that, unlike other studies we have seen in this area, our manuscript features extensive validation of our model behavior by comparison to key photosynthetic and carbon concentration metrics (i.e. predicted compensation points, quantum yields of photosynthesis, and rubisco saturation), we believe the reported results and the models we are presenting hold up and constitute a valuable contribution to the literature.

Reviewer #2 (Comments for the Author):

Thank you to the authors for careful consideration of the reviewer comments and increased details of the models and simulation. I have two further questions/recommendations but recommend the work for publication.

Author response: We are happy to see that the reviewer now recommends acceptance in Plant Physiology and are grateful for their input and recommendations. We have responded to

their remaining concerns below and noted where we have made additions to the text to address these concerns.

1. I think I now better understand how the thylakoid tubules are treated in the modeling for the CCM. It seems that this treatment may result in a different surface to volume ratio than is used in the Fai et al paper, which would effect transport rates of inorganic carbon between thylakoid and pyrenoid. Indeed the algal simulation with CCM is (highly?) sensitive to the radius of the thylakoid indicating that this simplification may have an effect. Could the authors clarify if the assumed tubule surface to volume ratios and in particular the surface area which will control the effective transport rate, are within the range of observed physiological values? I believe the geometry of the pyrenoid vary, so the default values assumed in Fei are not necessarily unique. Could the authors include any comments about implications of the sensitivity analysis results?

Author response: We have added some discussion on this point in lines **356-363**. Yes, the surface area/volume ratio and surface area of the tubules differs between our models and the Fei et al. model due to the simplified geometry used in this manuscript (noted in the Methods sections on lines **124-127**). As we explain in the manuscript, this change was made for numerical tractability**.** However, the local sensitivity to the thylakoid surface area does not appear to be high relative to other sensitivity values presented in **Fig 3** – increasing all permeabilities between the thylakoid lumen, stroma, and pyrenoid matrix by a factor of 10 to simulate an increased surface area/volume ratio (closer to that used in Fei et al.) results in only a **3.8%** change in rubisco saturation and a **6%** change in quantum yield with atmospheric CO2 and standard (i.e. Gutknecht) lipid membrane permeability. Together with the extensive validation results presented in this study, which suggest that the models are reproducing key photosynthetic and carbon concentration metrics as they are currently set up, we believe this simplification is suitable for the present study.

2. It is becoming more and more standard to make code available with publication of a model. Can the authors make their code or model definition files available upon publication? I encourage them to do so regardless of whether the journal requires it.

Author response: Yes – in addition to the existing instructions on how to access the models through the *Virtual Cell* program in the manuscript (**lines 637-639**) we have now also included the model files (.vcml) as part of the supplemental material **(lines 639-640)**.

11-May-2024
Prof. Yair Shachar-Hill
Michigan State University
Plant Biology
East Lansing, Michigan 48824-1312

MSID: PP2024-RA-00046R2
MS TITLE: Biophysical carbon concentrating mechanisms in land plants: insights from reaction-diffusion modeling

Dear Dr. Yair Shachar-Hill:

I am pleased to inform you that your revised manuscript "Biophysical carbon concentrating mechanisms in land plants: insights from reaction-diffusion modeling" has been accepted for publication in Plant Physiology. Your manuscript will be checked for consistency with journal formatting and image standards by one of our Science Editors, who will contact you shortly. They may have a number of queries and suggestions for you. Please keep in mind that they work to increase the online visibility of your article and its attraction for readers from the widest of backgrounds. You will have the opportunity to review any revisions, and I hope you will work constructively with them to get the most out of your research. You will also have the opportunity to review all changes when you receive the author proofs for your article.

Once validated for publication, your article will be sent to a Science Editor for a final review of your manuscript and source files. After your final files are uploaded, your article will appear in the next available issue and the online Preview version of your article will be posted after your signed Copyright and Checklist forms and final source files are received.

Please be sure to include the following in your manuscript file:
Harvard style citations
List of all author contributions and funding information at the beginning of manuscript after the titles and author list.
List all supplemental materials associated with the article, with titles, at the end of the ms, before the references and acknowledgments section.
Complete Figure Legends for all figures appearing in the article listed before the Literature Cited.

Thank you and congratulations on your Plant Physiology paper!

Sincerely,
Editor, Plant Physiology

=====
FIRST AUTHOR PROFILES
=====

We recognize the hard work that goes into being First Author of a Plant Physiology paper by publishing a "First Author Feature" for each First Author alongside your paper.

If the first author(s) would like to submit information for a feature on Facebook and Twitter, fill out the form by clicking on the link below:

Plant Physiology First Author Feature Form: https://forms.office.com/r/ZBCGZfhm8F
If you cannot access the form through the link above, please contact Rachel at rbelsky@aspb.org.

To upload an image of yourself to accompany your profile, please put your photo in the correct journal folder at the link below. You must label your image: Author First Name Last Name Article Title.

First Author Profile Image Dropbox: https://www.dropbox.com/scl/fo/xz029p7qjr2jml2rp2kmq/h?dl=0&rlkey=6fkxcaqmxx35ap152b2osy1fz

=====
IMPORTANT REMINDER: PEER REVIEW REPORTS

=====

If you opted to publish a peer review report along with your article during the original submission process, it will be prepared by the editorial staff and publicly posted with your manuscript, inside the zip file that contains any other supplemental material. As a reminder, the peer review report is a public record of all comments from editors and reviewers, as well as your prior responses, as you received them in the decision letters for each draft of your manuscript. If you agreed to publish this report and have changed your mind, or are not sure if you selected this option, please contact the editorial office as soon as possible before signing the license agreement from our publisher.

-- Reviewer comments:
